# Spaceflight Analogue Culture Enhances the Host-Pathogen Interaction Between *Salmonella* and a 3-D Biomimetic Intestinal Co-Culture Model

**DOI:** 10.3389/fcimb.2022.705647

**Published:** 2022-05-31

**Authors:** Jennifer Barrila, Jiseon Yang, Karla P. Franco Meléndez, Shanshan Yang, Kristina Buss, Trenton J. Davis, Bruce J. Aronow, Heather D. Bean, Richard R. Davis, Rebecca J. Forsyth, C. Mark Ott, Sandhya Gangaraju, Bianca Y. Kang, Brian Hanratty, Seth D. Nydam, Eric A. Nauman, Wei Kong, Jason Steel, Cheryl A. Nickerson

**Affiliations:** ^1^ Biodesign Center for Fundamental and Applied Microbiomics, Arizona State University, Tempe, AZ, United States; ^2^ School of Life Sciences, Arizona State University, Tempe, AZ, United States; ^3^ Genomics and Bioinformatics Research Unit, Agricultural Research Service, U.S. Department of Agriculture, Gainesville, FL, United States; ^4^ Bioinformatics Core Facility, Bioscience, Knowledge Enterprise, Arizona State University, Tempe, AZ, United States; ^5^ Division of Biomedical Informatics, Cincinnati Children’s Hospital Medical Center, Cincinnati, OH, United States; ^6^ Biomedical Research and Environmental Sciences Division, NASA Johnson Space Center, Houston, TX, United States; ^7^ Department of Animal Care & Technologies, Arizona State University, Tempe, AZ, United States; ^8^ School of Mechanical Engineering, Weldon School of Biomedical Engineering and Department of Basic Medical Sciences, Purdue University, West Lafayette, IN, United States; ^9^ Biodesign Center for Immunotherapy, Vaccines and Virotherapy, Arizona State University, Tempe, AZ, United States

**Keywords:** mechanobiology, fluid shear, Rotating Wall Vessel, spaceflight, colon, Typhimurium, Hfq, gene expression

## Abstract

Physical forces associated with spaceflight and spaceflight analogue culture regulate a wide range of physiological responses by both bacterial and mammalian cells that can impact infection. However, our mechanistic understanding of how these environments regulate host-pathogen interactions in humans is poorly understood. Using a spaceflight analogue low fluid shear culture system, we investigated the effect of Low Shear Modeled Microgravity (LSMMG) culture on the colonization of *Salmonella* Typhimurium in a 3-D biomimetic model of human colonic epithelium containing macrophages. RNA-seq profiling of stationary phase wild type and Δ*hfq* mutant bacteria alone indicated that LSMMG culture induced global changes in gene expression in both strains and that the RNA binding protein Hfq played a significant role in regulating the transcriptional response of the pathogen to LSMMG culture. However, a core set of genes important for adhesion, invasion, and motility were commonly induced in both strains. LSMMG culture enhanced the colonization (adherence, invasion and intracellular survival) of *Salmonella* in this advanced model of intestinal epithelium using a mechanism that was independent of Hfq. Although *S*. Typhimurium Δ*hfq* mutants are normally defective for invasion when grown as conventional shaking cultures, LSMMG conditions unexpectedly enabled high levels of colonization by an isogenic Δ*hfq* mutant. In response to infection with either the wild type or mutant, host cells upregulated transcripts involved in inflammation, tissue remodeling, and wound healing during intracellular survival. Interestingly, infection by the Δ*hfq* mutant led to fewer transcriptional differences between LSMMG- and control-infected host cells relative to infection with the wild type strain. This is the first study to investigate the effect of LSMMG culture on the interaction between *S*. Typhimurium and a 3-D model of human intestinal tissue. These findings advance our understanding of how physical forces can impact the early stages of human enteric salmonellosis.

## Introduction

Understanding infectious disease risks during spaceflight is critical for the design of strategies to maintain astronaut health and mission success. The microgravity environment of spaceflight induces widespread physiological changes in both humans and microorganisms that may increase risk for infection, including alterations in human immune responses (Crucian et al., 2015), gastrointestinal function (Thornton et al., 1987; Harm et al., 2002; Mcphee and Charles, 2010), microbiome composition (Voorhies and Lorenzi, 2016; Garrett-Bakelman et al., 2019; Voorhies et al., 2019) and increased bacterial virulence (Wilson et al., 2007; Wilson et al., 2008; Gilbert et al., 2020). However, there remains much to be understood regarding how this environment regulates host-pathogen interactions in humans at the phenotypic and molecular levels. Accordingly, the use of advanced *in vitro* models of human tissue that are more highly predictive of *in vivo* responses will facilitate an improved understanding of how biomechanical signals associated with spaceflight and spaceflight analogue environments are integrated at the cellular level. These findings hold potential to reveal novel infection mechanisms that cannot be obtained using conventional experimental approaches.

**Figure 1:**
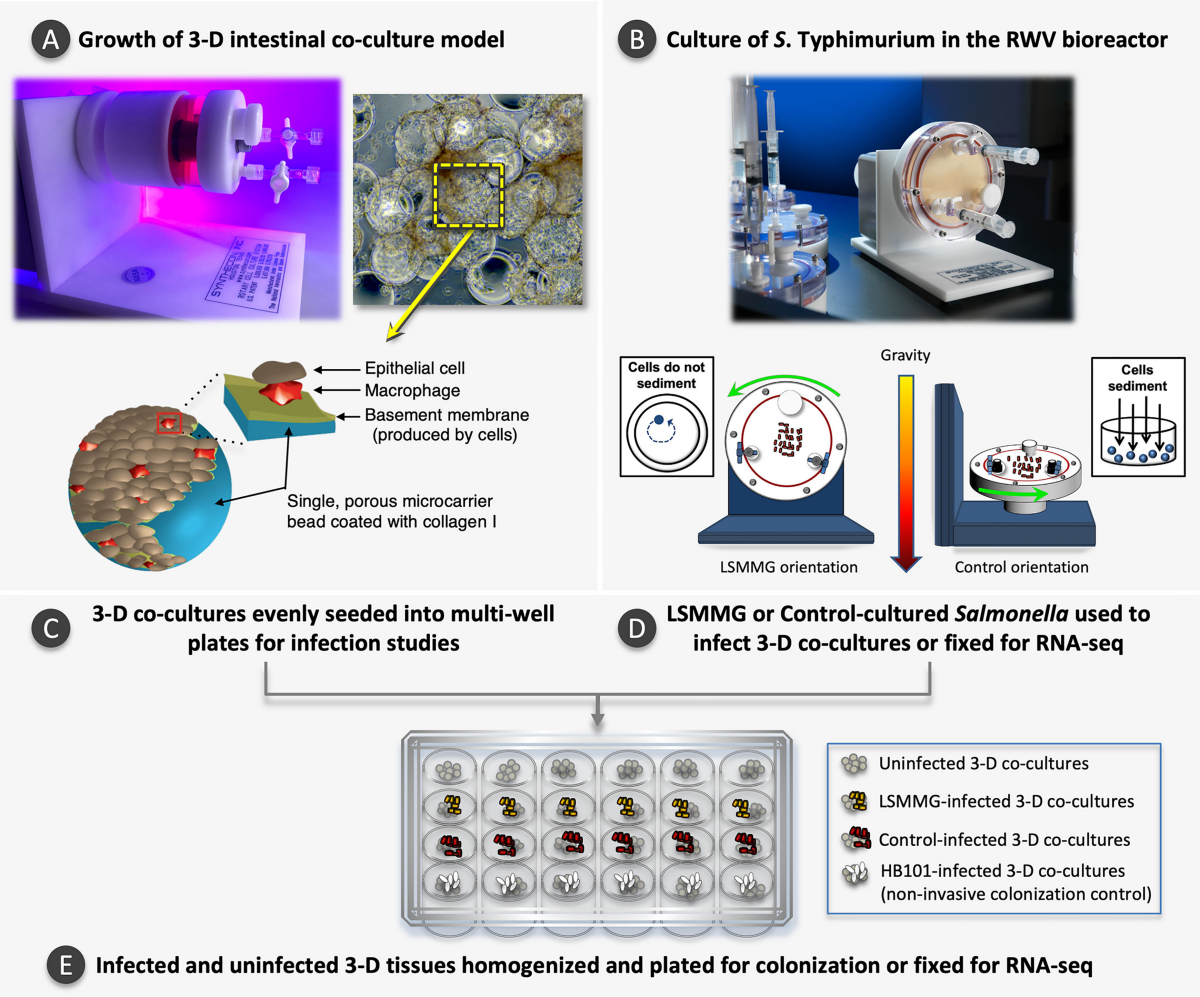
Experimental overview. **(A)** Photo of a slow turning lateral vessel (STLV) bioreactor used to grow the 3-D intestinal co-culture model. During model growth, cells attach to porous, collagen I-coated microcarrier beads. The graphic inset depicts a single microcarrier bead coated with collagen I, macrophages and epithelial cells. As the model matures, individual cell-bead complexes aggregate to form larger 3-D structures, as shown in the light micrograph (magnification 200X). The yellow box highlights a single microcarrier bead within a larger 3-D aggregate. STLV image reproduced with permission: ^©^ American Society for Microbiology, Infect Immun 86:e00282-18, 2018. Light micrograph and bead schematic reproduced with permission from Barrila and Yang et al., 2017 with slight modification of the labels and addition of the (Barrila et al., 2017) under CC by 4.0 (https://creativecommons.org/licenses/by/4.0/legalcode). **(B)** Photo of a High Aspect Ratio Vessel (HARV) bioreactor used to culture *S.* Typhimurium and a schematic depicting the two bioreactor orientations used in this study. In the LSMMG orientation, the solid body rotation of the media maintains the bacteria in suspension under low fluid shear conditions. In the control orientation, the bacteria sediment to the bottom of the vessel during rotation, experiencing increased fluid and frictional shear forces. Images reprinted by permission from Springer Nature, Barrila et al., 2016. **(C)** Once the 3-D models have fully differentiated, they are evenly seeded into multi-well plates. **(D, E)** *S*. Typhimurium is cultured to stationary phase under LSMMG or control conditions and used to infect a subset of the 3-D co-cultures for the gentamicin protection assay or fixed for RNA-seq. Controls included both time-matched uninfected host cells and a non-invasive HB101 strain. At specified time points, samples in individual wells are lysed, serially diluted, and plated to obtain colony counts or are fixed for RNA-seq analysis.

Over the past two decades, we have successfully used the Rotating Wall Vessel (RWV) bioreactor as a spaceflight culture analogue to predict responses of *Salmonella enterica* serovar Typhimurium (*S*. Typhimurium) to true spaceflight (Nickerson et al., 2000; Wilson et al., 2002a; Wilson et al., 2002b; Wilson et al., 2007; Wilson et al., 2008; Barrila et al., 2021). In particular, the low fluid shear suspension culture environment generated under LSMMG conditions (**Figure 1B**) has been highly predictive of virulence trends for *Salmonella* following spaceflight (Nickerson et al., 2000; Wilson et al., 2002a; Wilson et al., 2002b; Wilson et al., 2007; Wilson et al., 2008) and is also relevant to fluid shear forces encountered by enteric pathogens in the intestinal tract (Nauman et al., 2007). LSMMG culture of *S*. Typhimurium to late log phase significantly increased the virulence and globally altered the stress resistance and gene expression profiles of the pathogen relative to reoriented control cultures (Nickerson et al., 2000; Wilson et al., 2002a; Wilson et al., 2002b). Interestingly, the transcriptomic profiles at this phase of growth indicated that, despite the enhanced virulence phenotype, many known virulence factors were downregulated or unchanged in expression (Wilson et al., 2002b).

Our spaceflight experiments conducted aboard Space Shuttle missions STS-115 and STS-123 confirmed our ground-based RWV predictions that true microgravity culture increases the virulence of *S*. Typhimurium (Wilson et al., 2007; Wilson et al., 2008). While a few classic virulence genes were induced during these spaceflight studies, most were either downregulated or unchanged in their expression, suggesting potential involvement of undiscovered mechanisms (Wilson et al., 2007). These transcriptional data and subsequent use of isogenic mutant strains identified the RNA-binding chaperone Hfq as playing a key role in regulating multiple stress responses of *S*. Typhimurium under these conditions (Wilson et al., 2007). Hfq stabilizes interactions between small non-coding RNAs and mRNAs to globally mediate gene expression, environmental stress responses, and/or virulence in a variety of microbes (Majdalani et al., 2005; Gottesman et al., 2006; Guisbert et al., 2007; Sittka et al., 2007; Sittka et al., 2009). Hfq has also been implicated in the response of other bacteria to RWV culture and/or spaceflight (Crabbe et al., 2010; Castro et al., 2011; Crabbe et al., 2011; Grant et al., 2014).

Our subsequent study aboard STS-131 (STL-IMMUNE) provided a first look into how the microgravity environment regulates the interaction of *S*. Typhimurium with human tissue, using a 3-D model of human colonic epithelial cells (HT-29) cultured in hollow fiber bioreactors (Barrila et al., 2021). Spaceflight altered the global transcriptomic and proteomic profiles of both the uninfected and infected host cells and indicated the potential for a heightened spaceflight infection, which aligns with the spaceflight-induced increase in virulence that we previously observed in mice. Due to in-flight technical limitations, we were unable to perform a gentamicin protection assay, which is an important *in vitro* assessment to quantify the ability of a pathogen to adhere, invade and survive within host cells. Given this challenge, ground-based studies using the RWV bioreactor could serve as a valuable tool to model whether the spaceflight environment could alter the colonization abilities of this pathogen in human tissue.

To this end, the RWV is also used for establishing 3-D models of human tissue that are more physiologically relevant predictive testing platforms than monolayers (Barrila et al., 2010; Nickerson et al., 2016; Barrila et al., 2018). RWV-derived 3-D intestinal models mimic key characteristics of the differentiated structure and function observed *in vivo*, including spontaneous differentiation into multiple epithelial cell types, polarization and well-formed tight junctions (Nickerson et al., 2001; Carvalho et al., 2005; Honer zu Bentrup et al., 2006; Alcantara Warren et al., 2008; Radtke et al., 2010; Salerno-Goncalves et al., 2011; De Weirdt et al., 2012; Barrila et al., 2017). Using this technology, we previously developed a 3-D co-culture model of human colonic epithelium containing epithelial cells and macrophages (Barrila et al., 2017). This co-culture model was an advancement of our previous 3-D colon model derived solely from epithelial cells (Honer zu Bentrup et al., 2006; Radtke et al., 2010; De Weirdt et al., 2012), which differentiated into multiple epithelial cell types (enterocytes, goblet cells, M/M-like cells, Paneth cells) and exhibited infection phenotypes consistent with an *in vivo* enteric *Salmonella* infection not observed using conventional monolayers (Honer zu Bentrup et al., 2006; Radtke et al., 2010). The inclusion of functional macrophages was an important advancement given the critical importance of these immune cells in human enteric salmonellosis (Haraga et al., 2008; Xu et al., 2009). Both the 3-D co-culture model (HT-29 and U937) and the monotypic model (HT-29) were applied to study different *Salmonella* pathovars grown conventionally (i.e., shaking flasks) (Barrila et al., 2017). Colonization of all *Salmonella* strains was decreased in the co-culture model relative to the epithelial model, indicating antimicrobial function of macrophages. Pathovar-specific differences in colonization profiles and bacterial intracellular co-localization patterns with the different host cell types were also observed.

While the responses of *S.* Typhimurium to LSMMG culture are the best characterized of any microbial pathogen, none of these studies have been performed using 3-D models of human intestinal tissue, nor have they examined the global molecular responses of the host to LSMMG-cultured *S*. Typhimurium during infection. Thus, in the current study we profiled the colonization of LSMMG-cultured *S*. Typhimurium (wild type and Δ*hfq*) in our 3-D colonic co-culture model containing macrophages and applied a transcriptomic approach to obtain a global view of the dynamics of infection at a molecular level.

## Materials and Methods

### Human Cell Lines, Bacterial Strains, and Culture Media

The human colonic epithelial cell line, HT-29 (ATCC HTB-38, RRID : CVCL_0320), and the human monocytic cell line U937 (ATCC CRL-1593.2, RRID : CVCL_0007) (Sundstrom and Nilsson, 1976) were cultured at 37°C and 10% CO_2_ in GTSF-2 medium (Hyclone) supplemented with 10% heat-inactivated FBS (Fisher Scientific, 10-082-147), 2.5 mg/L ITS (Sigma-Aldrich, I1884) and 2.25 g/L sodium bicarbonate (Fisher Scientific, S233-500). Wild type *S*. Typhimurium χ3339, a mouse-passaged derivative of SL1344 (Gulig and Curtiss, 1987), and an isogenic Δ*hfq* mutant derivative of χ3339 were used in this study. *Escherichia coli* (*E. coli*) HB101 was used as a non-invasive control for infection studies. Bacteria were cultured in Lennox Broth (LB; Fisher Scientific, BP1427-2) for all experiments.

### Strain Construction

**Figure 2:**
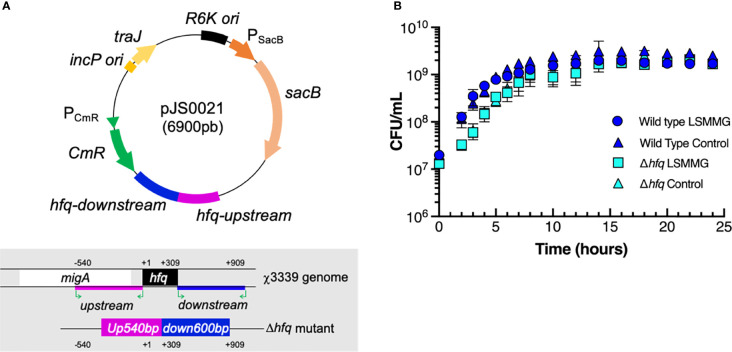
Δ*hfq* mutant construction and bacterial growth curves. **(A)** Plasmid map of pJS0021 used to construct the Δ*hfq* mutant and schematic overview showing the deletion of the *hfq* gene in χ3339. Pink and blue regions represent the PCR-amplified 540 base pair (bp) upstream and 600 bp downstream regions of the gene that were ligated to suicide vector pRE112 to construct pJS0021, as described in the materials and methods. **(B)** Growth curves of wild type *S*. Typhimurium and the Δ*hfq* mutant in RWV bioreactors positioned in the LSMMG or control orientations. CFU/mL = colony forming units per mL. Data points and error bars represent the mean value and standard deviation, respectively, from at least three independent biological replicates each plated in technical triplicate.

The *hfq* gene (309 base pairs/bp) was deleted from *S*. Typhimurium strain χ3339 with suicide vector pJS0021 using conjugation and allelic exchange (**Figure 2A**). A 504 bp region upstream of *hfq* was PCR-amplified using primers: 5′-CGCAAAGACTTTCCCG-3′ and 5′-TGCATCAGCAACTTCAGGAGATAG-3′. A 600 bp region downstream of *hfq* was also PCR-amplified using primers: 5′- AAAAGAGAGTTCACGCGCTGTTTATCCATG-3′ and 5′-GCGACTGAATCTGCACAATGCGAT-3′. The PCR-amplified upstream and downstream regions in χ3339 were ligated to suicide vector pRE112, generating pJS0021 (**Figure 2A**). Following sequence verification of pJS0021, plasmids were transformed into *E. coli* χ7213 (MGN617) (Roland et al., 1999) and then conjugated with the parent strain χ3339. The conjugants of χ3339::pJS0021 were selected on LB with 30 µg/ml chloramphenicol (Cm). Second crossovers were induced and selected with 5% sucrose. The Δ*hfq* mutant was confirmed to be Cm-sensitive and the deletion verified by PCR and sequencing.

### RWV-Derived 3-D Co-Culture Model

The 3-D co-culture model of human colonic epithelium containing macrophages was grown as previously described (Barrila et al., 2017). Briefly, 1 x 10^7^ U937 cells were combined with 0.25 g porous Cytodex-3 microcarrier beads (pre-swollen and sterilized; Sigma-Aldrich, C3275) and treated with 10 nM phorbol-12-myristate-13-acetate (PMA; Sigma-Aldrich, P8139) for 48 h to induce characteristics of terminally differentiated macrophages (Rovera et al., 1979). PMA-differentiated U937 cells bound to microcarrier beads were gently rinsed in triplicate with GTSF-2 media, combined with 2 x 10^6^ HT-29 cells and loaded into Slow Turning Lateral Vessels (STLV; **Figure 1A**). STLVs were then filled completely with culture media, the bubbles were removed, and the bioreactors incubated statically for 15-30 min prior to initiating rotation at 20 rpm. Cultures were monitored daily and bubbles removed if observed. GTSF-2 was replenished after the first 5 days and then every 24 h thereafter until harvest at 13-14 days for use in assays.

### RWV Culture of *S*. Typhimurium

Wild type and mutant *S*. Typhimurium were separately cultured in LSMMG and control RWV bioreactors using previously described procedures (Nickerson et al., 2000; Wilson et al., 2002a; Wilson et al., 2002b; Wilson et al., 2007; Wilson et al., 2008). For each strain the following procedures were performed: bacterial colonies grown on LB agar were inoculated into 5 mL sterile LB and grown for 15-16 h overnight with aeration (250 rpm) at 37°C. Overnight cultures were diluted 1:200 into fresh LB, loaded into two separate RWV bioreactors positioned in the LSMMG and control orientations (**Figure 1B**) and rotated at 25 rpm. Cultures used for infection assays were grown to stationary phase for 24 h at 37°C. Growth curves confirmed that RWV cultures were at the same phase of growth for all studies (**Figure 2B**). Growth curves were performed by inoculating RWVs as described above and at designated time points, the bioreactors were sampled, serially diluted and plated for viable CFU/mL on LB agar (Fisher Scientific BP1427-2 and 214530). LSMMG cultures were sampled without stopping rotation of the bioreactor, but since bacteria grown in the control reactors sediment to the bottom of the vessels, cultures in this orientation were briefly stopped and mixed by inverting the reactor prior to sampling at each growth curve time point. Data points and error bars represent the mean value and standard deviation, respectively, from at least three independent biological replicates each plated in technical triplicate.

### Infection Studies

3-D co-culture models were harvested from the STLV, counted and seeded into multi-well plates at a final density of ~1 x 10^6^ cells/well. These models were previously shown to maintain their 3-D architecture and differentiated function following their removal from the STLV and seeding for infection studies (Barrila et al., 2017). Bacterial colonization was assessed using gentamicin protection assays as previously described (Honer zu Bentrup et al., 2006; Radtke et al., 2010; Barrila et al., 2017). RWV-cultured bacteria were removed from the bioreactors and 10 µl of each culture was added to 3-D models (multiplicity of infection ranged from ~15-30 bacteria-to-host cell). For gentamicin protection assays, samples were plated for viable CFU/mL at 30 min (adherence), 3 h (invasion) and 24 h (intracellular survival). At each time point, host cells were washed in triplicate with HBSS and lysed with 0.1% sodium deoxycholate (Sigma-Aldrich, D-6750) that had been prepared in Dulbecco’s phosphate-buffered saline (Fisher Scientific, 14190144) and filter sterilized (0.22 µm). Lysates were serially diluted and plated on LB agar to assess CFU/mL. Gentamicin was added to select wells after the 30 min (50 µg/mL) and 3 h (10 µg/mL) time points to eliminate remaining extracellular bacteria. Time-matched controls included non-invasive *E. coli* HB101. Percent adherence, invasion and intracellular survival were calculated by normalizing the CFU/mL obtained at each time point to the initial bacterial inoculum for each strain and condition. Graphing and statistical comparisons were performed using Graphpad Prism 9. Results are expressed as mean ± standard deviation. Data were assessed for normality using the Shapiro-Wilk test and were subsequently analyzed using Kruskal−Wallis non−parametric ANOVA with Dunn’s multiple comparisons using an alpha of 0.05.

### RNA-Seq Analysis of 3-D Co-Cultures

At the specified time points, infected and time-matched uninfected 3-D co-cultures were fixed in 1 mL Qiazol (Qiagen, 79306), vortexed and stored at -80°C until processing. Host RNA-seq analyses were performed at each infection time point in biological and technical duplicate (two separate biological replicates, each using two independent wells of a multi-well plate, resulting in a total of 4 RNA-seq samples analyzed per culture condition). To extract total RNA, samples were thawed, incubated for 5 min at room temperature with intermittent vortexing, and added to a prepared High Density MaXtract tube (Qiagen, 129056). To each sample, 0.2 mL chloroform (Sigma-Aldrich, C2432) was added, and tubes were shaken for 15 s. Following a 3 min incubation at room temperature, samples were centrifuged at 12,000 x *g* for 15 min at 4°C. The aqueous phase was then transferred to an RNase-free microfuge tube and 1.5 volumes of 200-proof molecular biology grade ethanol (Sigma-Aldrich, E7023) was added and tubes were inverted to mix. Samples were applied to miRNeasy columns (Qiagen, 217004) and purified according to manufacturer’s instructions with a 20 min on-column DNase I treatment (Qiagen, 79254). Eluted samples were quantified using a Nanodrop spectrophotometer (ThermoFisher Scientific) and DNase-treated a second time using the TURBO DNA-free kit (Thermo Fisher Scientific, AM1907). RNA integrity was confirmed on an Agilent Bioanalyzer using the Agilent RNA 6000 Nano kit (5067-1511) and all samples had an RNA Integrity Number (RIN) > 9.

cDNA was prepared from total RNA using the Ovation RNA-Seq System V2 *via* single primer isothermal amplification (NuGEN, 7102-A01) and automated on the Apollo 324 liquid handler (Wafergen). cDNA was quantified on the Nanodrop (Thermo Fisher Scientific) and then sheared to approximately 300 bp fragments using the Covaris M220 ultrasonicator. Libraries were generated using the Kapa Biosystem library preparation kit (KK8201). Fragments were end-repaired and A-tailed as described in the Kapa protocol. Individual indexes and adapters (Bioo, 520999) were ligated onto each separate sample. Adapter-ligated molecules were cleaned using AMPure beads (Agencourt Bioscience/Beckman Coulter, A63883) and amplified with Kapa HIFI enzyme (KK2502). Each library was then analyzed for fragment size on an Agilent Tapestation and quantified by qPCR (KAPA Library Quantification Kit, KK4835) on Quantstudio 5 (ThermoFisher Scientific) before multiplex pooling and sequencing on a 1x75 flow cell on the NextSeq platform (Illumina) at the Arizona State University Genomics Core facility. RNA-seq reads for each sample were quality checked using FastQC v0.10.1 and aligned to the human reference genome GRCh38.p7 primary assembly using STAR v2.5.1b. A series of quality control metrics were generated on the STAR outputs. Stringtie-1.3.4c was used to report FPKM values (Fragments Per Kilobase of transcript per Million mapped reads), read counts and TPM (Transcripts Per Million). Differential expression (DE) analysis was performed with EdgeR package from Bioconductor v3.2 in R 3.2.3. Multi-dimensional scaling (MSD) plots were drawn by plotMDS, in which distances correspond to leading log-fold-changes between samples. For each pairwise comparison, genes with false discovery rate (FDR) < 0.05 were considered significant and log_2_-fold (logFC) changes of expression between conditions reported. Sequencing depth and mapped reads are available in **Supplementary Dataset 1**.

### RNA-Seq Analysis of Bacterial Cultures

Wild type and Δ*hfq* mutant *S*. Typhimurium were cultured to stationary phase in the RWV bioreactors as described above (**Figure 1B**, **Figure 2B**), fixed in RNAlater (Thermo Fisher Scientific, AM7021) at a 2:1 ratio (fixative:sample) and stored at -80°C until processing. The wild type and mutant bacterial RNA-seq assays were performed in biological triplicate (three independent cultures for each culture condition). Bacteria were pelleted and resuspended in 200 µL RNase-free TE buffer (ThermoFisher Scientific, T11493) containing 2 mg/mL lysozyme (Sigma-Aldrich, L6876). Samples were incubated for 10 min at room temperature with intermittent vortexing to facilitate lysis. Qiazol was added to each sample (700 µL) and incubated at room temperature for 5 min with intermittent vortexing. Samples were transferred into prepared MaXtract tubes (Qiagen, 129056), 0.2 µL chloroform added, and shaken for 15 sec to mix. Following a 3 min incubation at room temperature, samples were centrifuged at 12,000 x *g* for 15 min at 4°C. The aqueous phase was then transferred to an RNase-free microfuge tube and 1.5 volumes of 100% molecular biology grade ethanol (Sigma-Aldrich, E7023) was added to each sample and inverted to mix. Samples were applied to a miRNeasy column (Qiagen, 217004) and purified with on-column DNase I treatment (Qiagen, 79254). Eluted samples were quantified using a Nanodrop spectrophotometer and RNA integrity confirmed using an Agilent Bioanalyzer. RNA-seq library preparation was performed as described above. Sequencing reads were aligned to the *S*. Typhimurium LT2 genome from the NCBI database (https://www.ncbi.nlm.nih.gov/assembly/GCF_000006945.2) using the STAR/Stringtie pipeline for detection and quantification of transcripts.

### GO Biological Process and KEGG Pathway Enrichment Analysis

DAVID 6.8 (Huang et al., 2008) was used to perform enrichment analysis of RNA-seq data. Data were corrected using the Benjamini-Hochberg procedure, had threshold count of 2 and EASE score of 0.05.

## Results

### Transcriptional Analysis of RWV-Cultured Wild Type *S*. Typhimurium

**Figure 3:**
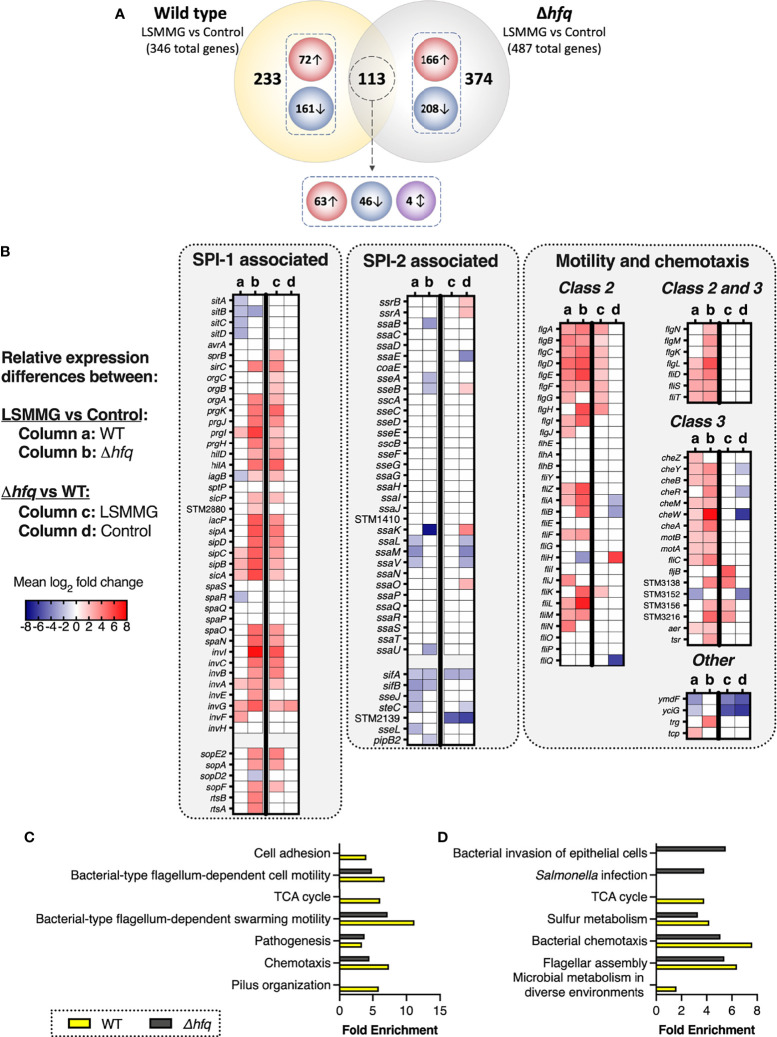
LSMMG-regulated genes in wild type and Δ*hfq S*. Typhimurium. Bacteria were cultured in RWVs positioned in the LSMMG or control orientations and analyzed using RNA-seq. Experiments were performed in biological triplicate for each strain (N = 3). Transcripts used in these analyses displayed a log_2_ fold change (logFC) of at least ± 1 and a false-discovery rate (FDR) of < 0.05. **(A)** Venn diagram depicting the numbers of distinct and common differentially expressed genes between WT and the Δ*hfq* mutant in response to LSMMG culture relative to their respective controls. Red circles indicate numbers of upregulated genes, blue circles indicate downregulated genes and the purple circle indicates genes oppositely regulated between wild type and mutant in response to LSMMG culture. **(B)** Heat maps highlighting differentially expressed genes associated with SPI-1, SPI-2 and motility/chemotaxis. Columns **(a)** and **(b)** indicate logFC in LSMMG cultures relative to controls for the wild type or mutant, respectively. Columns **(c)** and **(d)** indicate logFC in the mutant relative to wild type for either only the LSMMG cultures or only the control cultures, respectively. Mean logFC values are blue to indicate downregulation and red to indicate upregulation. White indicates no change for the indicated comparison. **(C‐D)** Enrichment analysis of WT (yellow bars) and Δ*hfq* (gray bars) transcriptomic data between LSMMG and control cultures. Biological processes are shown in panel **(C)** and Kegg pathways in panel **(D)** Differentially regulated transcripts were analyzed using DAVID 6.8 (Huang et al., 2008) using a threshold count of 2, an EASE score of 0.05 and Benjamini-Hochberg correction (< 0.05).

As we had not previously performed transcriptomic analysis of RWV-cultured *S*. Typhimurium at the phase of growth used in this study (stationary phase), we performed RNA-seq analysis of LSMMG and control cultures (**Figure 1B**, **Figure 2B**) to obtain a comprehensive picture of the global transcriptional profile of the pathogen just prior to infection. Growth curves confirmed that all cultures were in stationary phase of growth at the time point they were harvested for gene expression and infection studies (24 hours, **Figure 2B**). For the wild type, 346 genes were differentially expressed in response to LSMMG culture (**Figure 3A**, **Supplementary Table 1**; ≥ 2-fold threshold and false-discovery rate/FDR < 0.05). Gene ontology (GO) analysis revealed enrichment of transcripts associated with several biological processes, including motility and chemotaxis, pathogenesis, TCA cycle, cell adhesion and pilus organization (**Figure 3C** and **Supplementary Table 2**). KEGG pathway analysis revealed enrichment in pathways associated with flagellar assembly and chemotaxis, sulfur metabolism, TCA cycle and microbial metabolism in diverse environments (**Figure 3D**, **Supplementary Table 2**). Differential expression of transcriptional regulators, transporters and metabolic genes (e.g., redox homeostasis) was also observed.

Several genes associated with adherence and invasion were upregulated in LSMMG culture, including a subset encoded within *Salmonella* Pathogenicity Island (SPI)-1 (*prgI, sipC, sipB, sicA, invA, invG, invF*), SPI-5 (*sigE, sopB*), fimbrial genes (*fimA, fimC, fimZ*) and 31 transcripts associated with motility and chemotaxis (**Figure 3B**, **Supplementary Table 1**). Flagellar genes were either class 2 (middle assembly/hook-basal body) or class 3 (late assembly). The gene *stm0551*, which encodes a repressor for type 1 fimbriae and negatively regulates *fimA* (major fimbrial subunit) and *fimZ* (transcriptional regulator) (Wang et al., 2012), was downregulated; a trend which correlated with the upregulation of these fimbrial genes in LSMMG cultures. The small non-coding RNA, tmRNA (*stm2693;* encoded by *ssrA*), dually functions as both a tRNA and mRNA to regulate protein degradation and *Salmonella* pathogenesis (Julio et al., 2000; Ansong et al., 2009) was also upregulated in response to LSMMG.

Transcripts associated with intracellular survival and host transmission were downregulated in LSMMG culture (i.e., upregulated in the control culture), including: *sitABCD* (SPI-1-encoded manganese/iron transporter), several SPI-2-associated genes (*ssaL, ssaM, ssaV, sifA, sifB, sseJ, steC, sseL*), and several plasmid-encoded transcripts (e.g., *spvD, spvB, spvR*). Gifsy-1 prophage-encoded *sarA* (*Salmonella* anti-inflammatory response activator; *stm2585*) was also downregulated in response to LSMMG. SarA has been shown to promote intracellular replication/survival of the pathogen through STAT3-dependent induction of IL-10 (Jaslow et al., 2018). While SarA can be translocated through both the SPI-1 and SPI-2 type three secretion systems (T3SS), it is predominantly secreted by SPI-2 (Jaslow et al., 2018). Two anti-virulence genes in *S*. Typhimurium, *zirTS* (*stm1668* and *stm1669*, respectively), were also downregulated in response to LSMMG. ZirT (zinc regulated transporter) is an outer membrane protein that translocates ZirS (zinc regulated secreted protein) into the extracellular milieu (Gal-Mor et al., 2008). The inactivation of ZirTS has been shown to correlate with a hypervirulence phenotype in an oral infection in mice (Gal-Mor et al., 2008). ZirT was expressed at high levels within the murine intestine and during bacterial shedding in fecal pellets and at low levels during systemic infection (Gal-Mor et al., 2008). Collectively, these data suggest that LSMMG culture may better prime the wild type pathogen for attachment and invasion in the early stages of infection while in the long term, it is possible that the control cultures may ultimately be better adapted for intracellular survival and/or host-to-host transmission.

As some of the genes identified have known ties to the major stationary phase stress response regulator, RpoS, we compared our RNA-seq data obtained from wild type LSMMG and control cultures (**Supplementary Table 1**) to published genes belonging to the RpoS regulon under conventional shaking culture conditions. Approximately 20% (68 out of 346) of the genes are known to be regulated by RpoS (**Supplementary Table 3**), suggesting the possibility that the alternative sigma factor may also play a role in regulating responses of stationary phase *S*. Typhimurium to LSMMG conditions.

### Transcriptional Analysis of RWV-Cultured Δ*hfq*

Deletion of *hfq* significantly altered the relative gene expression profiles between LSMMG and control cultures as compared to the wild type (**Figure 3A**, **Supplementary Table 4**). Of the 487 genes differentially regulated by LSMMG culture, 374 (76.8%) were unique to the Δ*hfq* mutant, while 113 (23.2%) were also differentially regulated in the wild type. Most of these commonly shared transcripts were regulated in the same direction between the two strains and were associated with adherence, invasion and motility/chemotaxis (**Figure 3**, **Supplementary Tables 1** and **4**). Four of the shared genes were oppositely regulated in the mutant relative to the wild type in response to LSMMG culture, including *tmRNA* (*stm2693*) and d-galactonate transport protein, *dgoT*, which were each upregulated in LSMMG for the wild type but downregulated in LSMMG for the mutant (relative to control cultures). The SPI-1 gene *iagB* and predicted redox gene, *stm1666*, were both downregulated in LSMMG culture relative to the control for the wild type but upregulated in response to LSMMG for the mutant.

Similar to the wild type, LSMMG culture of the Δ*hfq* mutant was associated with differential regulation of select transcripts associated with SPI-1 (30 genes), SPI-4 (*siiA, siiB, siiC, siiD, siiE*), SPI-5 (*stm1089, copS, sigE, sopB*) and motility/chemotaxis (40 genes) (**Figure 3**, **Supplementary Table 4**). GO analysis indicated enrichment of biological process terms associated with pathogenesis, motility and chemotaxis (**Figure 3C**, **Supplementary Table 2**). Likewise, KEGG pathway analysis indicated enrichment in pathways associated with flagellar assembly, bacterial chemotaxis, sulfur metabolism, *Salmonella* infection and invasion of epithelial cells (**Figure 3D**, **Supplementary Table 2**). Like the wild type, class 2 and class 3 flagellar genes were mostly upregulated. Several SPI-2 associated genes/effectors were also downregulated in response to LSMMG culture (i.e., up in the control culture): *ssaB, ssaK, sseA, sseB, ssaU, pipB2, sifA, sifB*. Numerous plasmid-encoded genes were also downregulated. Also similar to the wild type, the anti-inflammatory response factor, *sarA*, and the secreted anti-virulence factor *zirS* were both downregulated in response to LSMMG culture; however, no difference in expression was observed for *zirT*.

### Identification of Distinct Hfq Regulons in LSMMG Versus Control Cultures

We compared the transcriptional profiles between wild type and mutant for either the LSMMG or control bacterial cultures to identify genes regulated by Hfq in each condition. LSMMG cultures of the Δ*hfq* mutant exhibited significant differences in 384 transcripts relative to wild type (**Supplementary Table 5**, **Supplementary Figure 1**). For the control cultures, 500 transcripts were differentially regulated between mutant and wild type (**Supplementary Figure 1**; **Supplementary Table 6**). Although a fraction of the Hfq-regulated genes identified for the two culture conditions did overlap, the composition of these regulons was profoundly impacted by the culture condition (**Supplementary Figure 1**).

Relative to wild type LSMMG cultures, Δ*hfq* LSMMG cultures were enriched for biological processes associated with pathogenesis and arginine biosynthesis and KEGG pathways associated with bacterial invasion (**Supplementary Table 2**). Surprisingly, the mutant exhibited enhanced levels in both the expression level and total number of genes associated with SPI-1, SPI-4, SPI-5 and motility (**Figure 3B**, **Supplementary Table 5**). These trends were not observed when the same comparison was made between the wild type and mutant control cultures (**Figure 3B**; **Supplementary Table 6**). These findings indicate that, while the Hfq protein does play a significant role in regulating the transcriptional response of *S*. Typhimurium to LSMMG culture, there are a core set of genes important for adhesion, invasion, and motility that are commonly induced by both strains, which may explain their enhanced colonization phenotype under these conditions.

### Enhanced Colonization of 3-D Co-Cultures by LSMMG-Cultured *S*. Typhimurium

**Figure 4:**
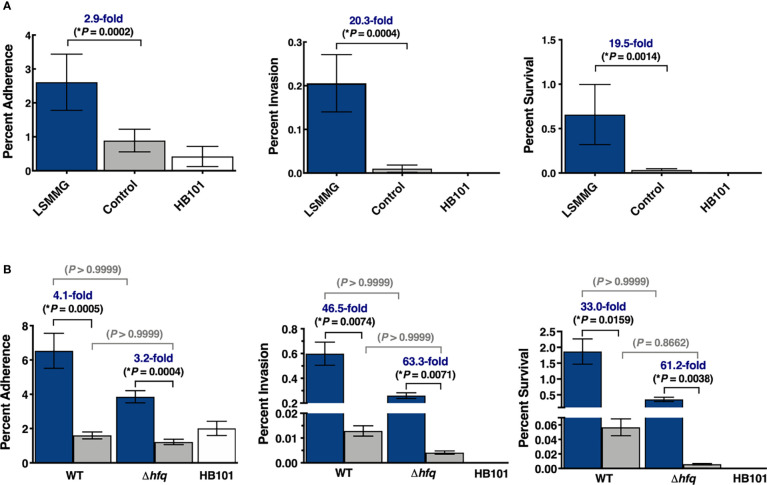
Enhanced colonization of 3-D co-culture model with LSMMG-cultured *S*. Typhimurium. **(A)** Wild type *S*. Typhimurium was cultured under LSMMG (blue bars) or as re-oriented controls (gray bars). Error bars represent standard deviation from two biological replicates, each in technical triplicate (N = 6). **(B)** Wild type *S*. Typhimurium and an isogenic Δ*hfq* mutant were independently cultured under LSMMG (blue bars) or as sedimented controls (gray bars). Error bars represent standard deviation from two biological replicates, each in technical duplicate (N = 4). Multiplicity of infection was ~15-30. *E. coli* HB101 (white bars), was also included as a non-invasive control for all experiments. At each time point, serial dilutions of the host cell lysate were plated to assess colony forming units (CFU/mL). All data were normalized to the initial bacterial inoculum for each strain/condition. Data were assessed for normality using the Shapiro-Wilk test and were subsequently analyzed using Kruskal-Wallis non-parametric ANOVA with Dunn’s multiple comparisons. (*) indicates *P* < 0.05 and is only shown for the *Salmonella* comparisons.

RWV cultures of *S*. Typhimurium were grown to stationary phase in the LSMMG and control orientations and used to infect our previously validated 3-D co-culture model of human colonic epithelium comprised of epithelial cells and macrophages (Barrila et al., 2017) (**Figure 1**). The LSMMG-cultured bacteria adhered, invaded and survived at significantly higher levels relative to the control bacterial cultures, with the largest differences occurring during invasion and survival (**Figure 4A**). We also evaluated the colonization of an isogenic Δ*hfq* mutant, since we previously determined that deletion of *hfq* disrupted LSMMG-dependent pathogenesis stress responses of *S*. Typhimurium, including responses to acidic pH and macrophage survival (Wilson et al., 2007). Interestingly, in our current study the *hfq* mutation did not significantly impact the colonization trends observed between the LSMMG and control in the 3-D co-culture model (**Figure 4B**).

### Host Response to Infection With Wild Type *Salmonella*

**Figure 5:**
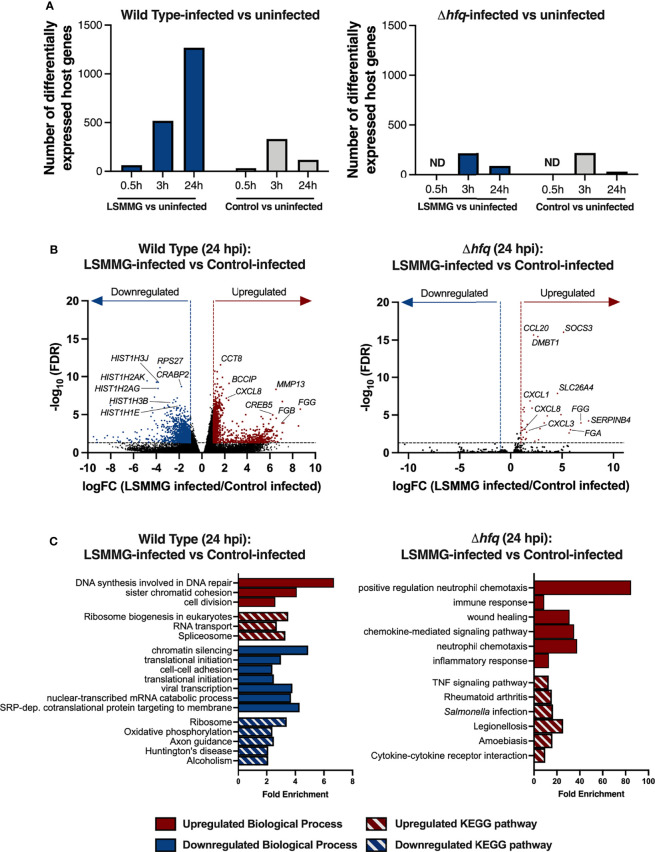
Response of 3-D co-culture model to infection with LSMMG- or control-cultured *S*. Typhimurium. **(A)** Number of differentially regulated transcripts between infected and uninfected host cells. Differentially expressed genes were determined by log_2_ fold change (logFC) cut-off of at least ± 1 and FDR < 0.05. Blue columns indicate differentially regulated transcripts in LSMMG-infected host cells relative to uninfected cells and gray columns indicate control-infected host cells relative to uninfected cells. ND indicates not determined. **(B)** Volcano plots of host transcriptional changes at 24 hours post-infection. Differentially expressed genes between LSMMG-infected versus control-uninfected host cells for the wild type (left panel) or *hfq* mutant (right panel) are shown. Red: significantly upregulated genes during LSMMG infection. Blue: significantly downregulated genes during LSMMG infection. Significance determined according to a logFC in expression of at least ± 1 and FDR <0.05. **(C)** Enriched GO Biological Processes and KEGG pathways for LSMMG-infected versus control-infected host cells at 24 hpi. Analysis was performed in DAVID 6.8 using upregulated (red) or downregulated (blue) transcripts using an EASE score of 0.05 and Benjamini-Hochberg correction to determine significantly enriched terms and pathways (adjusted *P < *0.05). Red bars indicate upregulated biological processes (solid) or KEGG pathway (hatch). Blue bars indicate downregulated biological processes (solid) or KEGG pathway (hatch).

RNA-seq profiling of the host following infection with the wild type revealed differences between infected (LSMMG or control bacteria) and uninfected cells at all time points (**Figure 5A**; **Supplementary Datasets 2**–**4** **)**. However, significant differences in the direct comparison between LSMMG-infected and control-infected cells were not observed until 24 hours post-infection (hpi) (**Supplementary Dataset 4**). While the LSMMG-infected cells displayed a progressive increase in the number of transcriptional differences relative to uninfected over time, cells infected with the control-cultured *Salmonella* peaked at 3 hpi and decreased at 24 hpi (**Figure 5A**). Consistent with a *Salmonella* infection of host cells, we observed rapid upregulation of mRNAs encoding *CXCL1* (GROα), *CXCL2* (macrophage inflammatory protein 2-alpha) and *CXCL8* (IL-8) for both LSMMG-infected and control-infected host cells by 30 minutes post-infection (mpi) relative to the uninfected cells (**Supplementary Dataset 2**). mRNAs for these chemokines were further upregulated at the later time points (**Supplementary Datasets 3** and **4**). By 3 hpi, infection with either the LSMMG or control cultures was linked with the enrichment of genes associated with several biological processes, including translation, chromatin silencing, inflammatory responses, response to lipopolysaccharide and cell-cell adhesion (**Supplementary Dataset 3**, **Supplementary Table 7**). Other genes known to be associated with *Salmonella* infection were also upregulated, including a variety of chemokines, *RELA, ARPC4* and *MAPK3*.

Direct comparison between LSMMG-infected and control-infected host cells at 24 hpi showed the upregulation of 996 transcripts and downregulation of 1,277 transcripts for LSMMG-infected cultures (**Figure 5A** and **Figure 5B**; **Supplementary Dataset 4**). Transcripts associated with inflammation, tissue remodeling, and wound healing were highly upregulated in LSMMG-infected cells compared to control-infected cells, including *CXCL1, CXCL8, IL-1β*, *IL1R1*, *IFIT1*, *FGG*, *FGB*, *MMP13*, *SPP1*, *NMU*, *CPM*, *C9* and *IBTK*, among others. Twenty-six transcripts encoding histone proteins were differentially regulated in LSMMG-infected cells, with 21 downregulated in expression relative to the infected control (**Figure 5B**; **Supplementary Dataset 4**). Several genes involved in retinoic acid metabolism (*CRABP2*, *STRA6, LIPE*, *DHRS3*, *MDK*, and various aldehyde dehydrogenases) were also downregulated. KEGG pathway analysis of the upregulated genes in LSMMG-infected cells indicated enrichment in pathways associated with cell division and DNA repair (**Figure 5C**; **Supplementary Table 8**). In addition to the upregulated genes associated with DNA damage, repair and replication that were observed (e.g., *BRCA2, RAD17, RAD51AP1, RAD51D, XRCC4*), transcripts encoding caspases 3 and 6 (*CASP3, CASP6*) were both upregulated in the LSMMG-infected cultures relative to the control-infected cells, suggesting increased apoptosis in LSMMG-infected cells. Along these lines, several cyclins and cyclin-dependent kinases were also upregulated in expression in the LSMMG-infected cultures, including *CCNC* (cyclin C), *CCNJ* (cyclin J) and *CDK4* (cyclin dependent kinase 4). In response to oxidative stress, the transcription factor cyclin C leaves the nucleus and induces mitochondrial hyperfission and apoptosis (Wang et al., 2015).

### Host Responses to Infection With the Δ*hfq* Mutant

We also evaluated the host response to infection with the Δ*hfq* mutant cultured in the LSMMG or control orientation. Given the small number of transcriptional changes observed for the wild type at the adherence time point, we focused on profiling only the responses during invasion and survival for the mutant. Infection with either LSMMG- or control-cultured Δ*hfq* led to a higher number of transcriptional differences in the host during invasion than at the survival time point; a transcriptional pattern similar to what was observed for cells infected with the wild type control cultures (**Figure 5A**). Like the wild type, infection with the mutant cultured in either the LSMMG or control condition elicited robust upregulation of genes associated with inflammation, response to LPS, cell chemotaxis, NF-κB and TNF signaling relative to the time-matched uninfected culture (**Supplementary Dataset 3**, **Supplementary Table 7**). Many of the genes belonging to these categories were also upregulated in response to infection with the wild type.

Although the LSMMG-cultured mutant survived at high levels in the model (**Figure 4B**), it induced a small number of transcriptional changes in host cells at 24 hpi when compared to those observed for the wild type (**Figure 5A**). The transcriptional profiles of both the LSMMG-infected and control-infected cultures were dominated by inflammatory responses associated with LPS exposure and neutrophil chemotaxis. Direct comparison of the LSMMG-infected and control-infected Δ*hfq* cultures indicated a modest difference in the host response between the two conditions at 24 hpi, with 30 genes all upregulated in LSMMG-infected cells compared to the control-infected host (**Supplementary Dataset 4**). These upregulated genes were largely associated with inflammation, wound healing and neutrophil chemotaxis (**Supplementary Table 9**). Consistent with the higher levels of colonization, mRNAs for several cytokines and cytokine receptors were upregulated in the LSMMG-infected cells, including *CXCL1, CXCL3, CXCL8, TGFB3, IL17C, IL19, CCL20* and *IL1R1*.

Although the relative host transcriptional profiles of the wild type and mutant largely differed from each other when each LSMMG-infected culture was compared to their respective control, there were a small number of genes that were commonly induced in response to infection with LSMMG-cultured *Salmonella*, including *CXCL1, CXCL8, IL1R1, FGG, SPP1, DMBT1, SERPINB3, NNMT*, and *SOCS3*. The enhanced expression of these inflammatory genes for both strains correlated with the enhanced colonization by both wild type and mutant LSMMG cultures in the infected host cells relative to their respective controls.

## Discussion

Understanding the dynamic interplay between physical forces, pathogen behavior and molecular responses during infection has the potential to unveil new targets for anti-virulence strategies to combat infectious disease. Infection prevention and mitigation are important to consider as astronauts return to the moon and travel onward to Mars. Comprehensive investigations into how infection processes may change during spaceflight are also crucial given the rapid expansion of the commercial sector, which will soon enable civilians more routine access to low Earth orbit. Although there have been several studies evaluating interactions between either bacteria or purified LPS with astronaut immune cells collected from peripheral blood (Kaur et al., 2005; Kaur et al., 2008; Crucian et al., 2011; Crucian et al., 2015), there is little known regarding how the human intestinal tract specifically responds to infection with pathogens like *Salmonella* cultured in the microgravity or simulated microgravity environment.

In this study, we evaluated how spaceflight analogue culture impacted the earliest stages of *Salmonella* infection in a 3-D co-culture model of human colonic tissue. LSMMG culture enhanced the colonization of wild type *S*. Typhimurium in the 3-D model. This finding aligns with our previous studies for this bacterial strain, which demonstrated increased virulence in mice following both LSMMG (Nickerson et al., 2000) and spaceflight culture (Wilson et al., 2007; Wilson et al., 2008), as well as a heightened response to infection by human 3-D colonic epithelial cells during spaceflight (Barrila et al., 2021). Increased invasion of *S*. Typhimurium 14028 was also previously observed in murine macrophages and human ovarian epithelial cells, as well as increased virulence using an anti-orthostatic tail suspension mouse model (a spaceflight analogue) (Chopra et al., 2006).

LSMMG cultures exhibited increased expression of bacterial genes important for *Salmonella* adherence and invasion, as well as many genes involved in motility and chemotaxis. Motility and chemotaxis are important for *Salmonella* pathogenesis, facilitating the movement of the pathogen within different microenvironmental niches of the host; though the extent of this dependence on the infection process can vary by strain, host or other factors (Carsiotis et al., 1984; Lockman and Curtiss, 1990; Stecher et al., 2004; Radtke et al., 2010; Yang et al., 2015). We and others have observed changes in the expression of motility and/or chemotaxis genes in response to spaceflight and LSMMG culture and it has been proposed that flagella could mediate responses of motile microbes to these conditions (Wilson et al., 2002b; Benoit and Klaus, 2007; Tucker et al., 2007; Wilson et al., 2007; Wilson et al., 2008; Crabbe et al., 2010; Crabbe et al., 2011; Kim et al., 2013; Acres et al., 2021). For example, flagella-mediated motility was shown to be essential in the formation of a unique column-and-canopy biofilm architecture by *Pseudomonas aeruginosa* during spaceflight (Kim et al., 2013). In addition, LSMMG culture of an *Aliivibrio fischeri* (*Vibrio fischeri*) *Δhfq* mutant showed upregulated expression of transcripts related to flagellar structure and assembly (Duscher et al., 2018). Mechanistic studies into the regulatory role of the flagellar system in the LSMMG responses of *S*. Typhimurium are ongoing in our laboratory.

We also observed several commonalities between host cell responses to infection with *Salmonella* grown under spaceflight analogue conditions and those observed when infections took place in true spaceflight during our STL-IMMUNE experiment (Barrila et al., 2021). Infection of our 3-D HT-29 co-culture model with wild type *S.* Typhimurium LSMMG cultures induced host transcriptional pathways associated with inflammation, tissue remodeling, and wound healing relative to control-infected cultures by 24 hpi. During our previous STL-IMMUNE study, we also found that spaceflight infection of HT-29 cells with this same strain of *S*. Typhimurium upregulated host genes belonging to these processes relative to ground infections (Barrila et al., 2021). For example, both LSMMG and spaceflight infections upregulated inflammatory genes *CXCL8* (IL-8) and *PTGS2* (COX2) as well as *IFIT1*, a positive regulator of type I interferon signaling involved in limiting LPS-associated inflammation in human macrophages (John et al., 2018). *HDAC2*, which encodes a protein that complexes with IFIT1, was also upregulated in LSMMG-infected host cells. Similarly, astronaut immune cells challenged post-flight with purified *E. coli* LPS induced IL-8 expression (Kaur et al., 2008; Crucian et al., 2015), though there have been select missions where IL-8 levels decreased following a similar challenge (Crucian et al., 2011). Increased expression of IL-8 also occurred when human endothelial cells were exposed to purified *E. coli* LPS during spaceflight, although host genes encoding proteins involved in early LPS uptake were suppressed (Chakraborty et al., 2014). We did not observe the latter trend in our data. The type of pathogen, use of a live pathogen, exposure time and/or the type of host cell used could be responsible for study-specific differences.

Both our STL-IMMUNE and LSMMG infections also upregulated putrescine biosynthesis genes (*ODC1*, *GLS*) and downregulated *AOC1*, which encodes an enzyme that catalyzes polyamine degradation (including putrescine). Polyamines are ubiquitous polycationic compounds that bind to and regulate the activities of nucleic acids, proteins and lipids to modulate a wide range of cellular processes, including cell growth and proliferation, metabolism, immune responses, apoptosis and redox activity (Munoz-Esparza et al., 2019). Spaceflight and LSMMG infections also commonly suppressed genes involved in retinoid metabolism (*STRA6, LIPE*, *DHRS3*, *MDK*, and aldehyde dehydrogenases). During our STL-IMMUNE study, spaceflight also downregulated retinoic acid-responsive genes in uninfected HT-29 cells, including those encoding mechanosensitive small proline-rich repeat proteins (SPRR) and those involved in type I interferon signaling/antiviral defense. Retinoic acid, an active metabolite of Vitamin A, regulates cell growth and differentiation and can modulate immune responses in either a protective or pathogenic capacity depending on its concentration (Grizotte-Lake et al., 2018). During infection, retinoids facilitate epithelial barrier repair and are important immunomodulators in the intestine (Derebe et al., 2014). Vitamin A deficiency tends to increase susceptibility to gastrointestinal infection and alternatively, infection can lead to its depletion (Derebe et al., 2014; Guo et al., 2015). The administration of retinoic acid has been shown to decrease the severity of *S.* Typhimurium-mediated gastroenteritis in mice (Sinha et al., 2016).

Multiple genes associated with cell death, cell cycle and DNA damage response were upregulated in host cells during infection with wild type LSMMG cultures, including caspases 3 and 6. *S.* Typhimurium infections are often associated with cell cycle arrest and increased levels of apoptosis in intestinal epithelial cells (Monack et al., 1996; Schwan et al., 2000; Fink and Cookson, 2007; Na et al., 2015). In macrophages, *Salmonella* infection is associated with pyroptosis, a pro-inflammatory, caspase-1 mediated programmed cell death (Fink and Cookson, 2007). In a previous study where we used this same 3-D co-culture model infected with *S.* Typhimurium grown as shaking cultures (Barrila et al., 2017), our microscopic data revealed a lack of macrophages following infection in the model by 24 hpi for strain SL1344 (the parental strain of the wild type χ3339 isolate used in this study). Although our global gene expression data in our present study did not indicate engagement of the caspase-1 pathway, we did observe a slight upward trend (not statistically significant) in the expression of caspase-1 and gasdermin E transcripts in LSMMG-infected cells relative to either the control-infected cultures (*CASP1*: logFC 1.69, FDR 0.066; *DFNA5*: logFC 3.74, FDR 0.16) or the uninfected controls (*CASP1*: logFC 1.78, FDR 0.067 and *DFNA5*: logFC 2.91, FDR 0.50). While no trends were observed for the well-studied pyroptosis executor gasdermin D, gasdermin E belongs to the same family of pore-forming proteins and has been shown in chemotherapeutic studies to be activated by caspase-3, which is then proposed to switch the cell death program from apoptosis to pyroptosis (Kovacs and Miao, 2017; Wang et al., 2017). These trends indicate pyroptosis may be occurring in a subpopulation of cells within the model or in the process of switching to this program. It is important to note that transcriptional results were likely dominated by the numerous epithelial cells over the macrophages in the model. Future studies using single cell RNA-seq could enable the dissection of the distinct and overlapping transcriptional programs elicited in different host cell types in the model following infection.

Many genes encoding histone proteins were also downregulated in LSMMG-infected cells relative to control-infected cultures. Histone gene expression is tightly coordinated with DNA replication (Christopher et al., 2016) and DNA damage is one factor that has been shown to induce the downregulation of these genes (Su et al., 2004). Additionally, pathogens are known to strategically target host gene expression using a number of mechanisms that include epigenetic regulation of the host, including histone modifications and host chromatin remodeling (Hamon and Cossart, 2008; Al Akeel, 2013; Silmon de Monerri and Kim, 2014; Pereira et al., 2016).

In addition to these changes, we also observed other transcriptional differences in host cells infected with wild type LSMMG cultures relative to control cultures that aligned with the enhanced colonization phenotype. For example, fibrinogen gamma chain (*FGG*) and fibrinogen beta chain (FGB) were among the most highly upregulated transcripts in LSMMG-infected cells versus control-infected cells. Fibrinogen is expressed by a variety of cell types, including epithelial cells (Molmenti et al., 1993; Simpson-Haidaris et al., 1998). Following tissue damage and inflammation, fibrinogen can either serve as an antimicrobial mediator to restrict pathogen growth and dissemination using either physical entrapment or recruitment of immune cells to facilitate pathogen killing, or conversely, promote infection by enabling pathogen adherence to host tissues (van Hemert et al., 2006; Adams et al., 2007; Ko and Flick, 2016). The *MMP13* transcript and its regulator, *SPP1* (osteopontin) were also upregulated. MMP13 mediates LPS-induced intestinal permeability through tight junction destabilization and mucus depletion, leading to systemic inflammation (Vandenbroucke et al., 2013; Li et al., 2016). LSMMG-infected cells also displayed upregulation of *NMU*, which encodes the multifunctional neuropeptide Neuromedin U, which is associated with the gut-brain axis, intestinal motility and stress responses (Martinez and O'Driscoll, 2015). This peptide is expressed in enteroendocrine cells (Sinagoga et al., 2018) and is upregulated in macrophages following challenge with LPS, acting as an inflammatory mediator to promote endotoxin shock (Moriyama et al., 2006). Interestingly, *NMB*, which encodes a closely related neuropeptide, was downregulated in LSMMG-infected cells.

Our colonization studies revealed that LSMMG conditions enabled high levels of colonization by an isogenic Δ*hfq* mutant, with no significant differences relative to wild type (though there was a slight downward trend at all time points). These findings were unexpected given the critical role of Hfq in *Salmonella* virulence in mice and invasion into monolayers when grown in flasks (Sittka et al., 2007) and our previous discovery that Hfq regulates the LSMMG responses of this strain to acid stress and macrophage survival in monolayers (Wilson et al., 2007), and thus reveals heterogeneity in the regulatory role of this gene in the LSMMG responses of *S*. Typhimurium. This complexity is further highlighted by a separate study that used a different *S.* Typhimurium background (14028s) and showed that Hfq did not impact the LSMMG acid stress response phenotype (Pacello et al., 2012), though there were a number of experimental differences between this work and our previous study (e.g., strain, culture time, pH) (Wilson et al., 2007). One of the known major consequences of the *hfq* mutation is chronic envelope stress that likely weakens the pathogen (Sittka et al., 2007). Since many of the previous studies performed with the *hfq* mutant showing differences in colonization involved subjecting the bacteria to high fluid shear forces in shaking flasks prior to exposure to a specific stressor or infection, the low fluid shear force environment within the RWV bioreactor may be “gentler” on the already weakened microbe. This would likely be true for both the LSMMG and control bioreactor conditions, but especially the LSMMG condition, where the bacteria are fully in suspension within the culture media without agitation.

RNA-seq analyses confirmed that, although the relative differences between *hfq* mutant LSMMG and control cultures were largely distinct from those of the wild type, a core set of genes involved in adherence, invasion and motility/chemotaxis were commonly upregulated under LSMMG conditions for both strains, in alignment with their enhanced colonization trends. The number of genes belonging to these categories and extent of their upregulation was greater in the mutant than wild type LSMMG cultures. This finding was intriguing since under conventional culture (non-RWV) conditions, Sittka et al. found that deletion of *hfq* severely impacted the invasion of *S*. Typhimurium in monolayers (Sittka et al., 2007) and downregulated motility and chemotaxis genes, as well as genes belonging to SPI 1-5 (Sittka et al., 2009). Interestingly, the authors observed that the effect of the mutation was less severe under SPI-1 inducing conditions (i.e., low oxygen, high salt) and that ectopic expression of the major SPI-1 regulator HilD enabled the bacterium to partially overcome invasion defects (Sittka et al., 2009). Under the conditions of our study, the LSMMG environment was highly permissive for SPI-1 gene expression in the *hfq* mutant, increasing the expression of both *hilD* and *hilA* relative to the wild type (~8- and ~30-fold, respectively). One limitation in our study was the average fragment size of our RNA-seq libraries (~200 nt), which precluded our ability to use these data to reliably evaluate global changes in small non-coding RNA expression in the wild type and *hfq* mutant in response to LSMMG culture. Targeting smaller library sizes in the future will enable deeper insight into the effect of modeled microgravity culture on the expression of these key regulators. This will be important given the differential expression of several small non-coding RNAs previously reported during spaceflight culture of the same wild type strain (Wilson et al., 2007).

When we evaluated the host response to infection with the *hfq* mutant, we found the number of transcriptional differences between LSMMG-infected and control-infected cells were much smaller than those observed during wild type infections. Like the wild type, differentially expressed genes were largely involved in inflammation, wound healing, and neutrophil chemotaxis. In alignment with these findings, previous *in vivo* data has shown that an *S*. Typhimurium *hfq* mutant grown under conventional conditions can still efficiently stimulate wild type-like immune responses against LPS and outer membrane proteins (Allam et al., 2011). In the current study, the transcriptional kinetics of the host in response to infection with the *hfq* mutant relative to time-matched uninfected host cells peaked at 3 hpi, with very few changes in gene expression observed for either LSMMG or control-infected cultures by 24 hpi, which may be reflective of the beginning stages of a successful resolution of infection by the host.

Since the model used in our current study is designed to evaluate the initial stages of infection in the host and has a limited repertoire of immune cells compared to the *in vivo* scenario, future studies should explore whether hierarchical incorporation of additional host cell types and microenvironmental factors in the model or just prior to infection (e.g., low pH, low oxygen, microbiota, antimicrobial peptides) would modulate these trends. For example, the incorporation of astronaut immune cells collected during or just after spaceflight into the model may change the colonization or gene expression patterns. This is an important consideration given the changes known to occur in monocytes in humans during spaceflight, including reductions in their ability to phagocytose *E. coli*, elicit oxidative bursts and degranulate (Kaur et al., 2005). In addition, given the known importance of RpoS during stationary phase for *Salmonella* under conventional shaking culture conditions, and the number of RpoS-regulated genes that were differentially expressed between LSMMG and control cultures, we have also investigated the role of this sigma factor in the LSMMG response of this strain during stationary phase (Franco Meléndez et al., manuscript submitted). We previously demonstrated that the pathogenesis-related responses of *S*. Typhimurium in the RWV at late exponential/early stationary phase were independent of RpoS (Wilson et al., 2002a). In a separate study, LSMMG responses for *E. coli* were also found to be RpoS-independent during exponential phase, but a dependence on RpoS was observed during stationary phase for several pathogenesis-related stressors (Lynch et al., 2004).

In summary, our findings demonstrate that LSMMG culture enhanced the host-pathogen interaction between *S*. Typhimurium and a human 3-D co-culture model of intestinal epithelium during the earliest stages of infection in an Hfq-independent manner. Collectively, these findings add to the growing body of literature demonstrating the importance of incorporating physical force considerations and advanced biomimetic 3-D tissue culture models into *in vitro* infectious disease studies. These findings further reinforce the critical role of mechanotransductive forces as microenvironmental signals in reprogramming *S.* Typhimurium responses at both the molecular and phenotypic levels to regulate key infection phenotypes that are relevant to both astronaut health and the general public.

## Data Availability Statement

The gene expression datasets presented in this study can be found in online repositories. The names of the repository/repositories and accession number(s) can be found below: https://www.ncbi.nlm.nih.gov/geo/query/acc.cgi?acc=GSE146347, GSE146347 and https://genelab-data.ndc.nasa.gov/genelab/accession/GLDS-277/, GLDS-277.

## Author Contributions

Designed research: CN, JB, CMO, BA, JY, WK, JS, HB, and TD. Performed research: JB, JY, KFM, TD, RD, RF, SG, BK, SN, CN, and EN. Analyzed data: JB, SY, KFM, BA, CN, RD, SG, BK, BH, HB, CMO, EN, and KB. Wrote the paper: JB. Edited paper: JB, CN, CMO, KFM, SN, BK, JS, SY, HB, JY, and KB. All authors contributed to the article and approved the submitted version.

## Funding

Funding for this study was supported by NASA grants NNX13AM01G (CN, JB, CMO, and BA), NNX15AL06G (CN, JB, and CMO) and 80NSSC18K1478 (CN, JB, CMO, and HB; includes NASA PECASE funding to JB).

## Conflict of Interest

The authors declare that the research was conducted in the absence of any commercial or financial relationships that could be construed as a potential conflict of interest.

## Publisher’s Note

All claims expressed in this article are solely those of the authors and do not necessarily represent those of their affiliated organizations, or those of the publisher, the editors and the reviewers. Any product that may be evaluated in this article, or claim that may be made by its manufacturer, is not guaranteed or endorsed by the publisher.
