# Supplementary figures and images for: Spaceflight Analogue Culture Enhances the Host-Pathogen Interaction Between Salmonella and a 3-D Biomimetic Intestinal Co-Culture Model
Source: Front Cell Infect Microbiol. 2022 May 31;12:705647. doi: 10.3389/fcimb.2022.705647 (PMC9195300; doi:10.3389/fcimb.2022.705647)

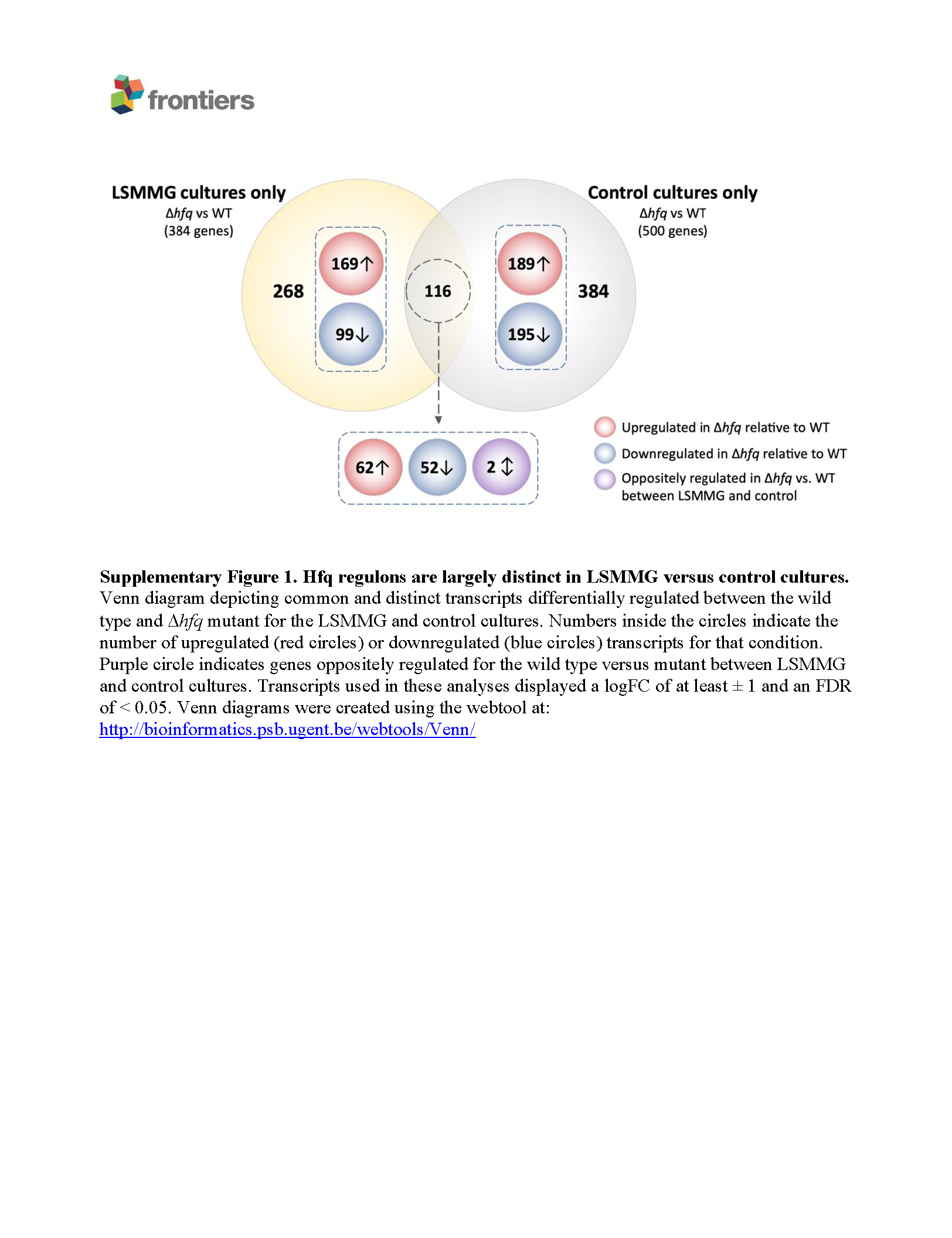

Supplement: Supplementary file 5 [file Image_1.tiff]
