# Supplementary material for: Spaceflight Analogue Culture Enhances the Host-Pathogen Interaction Between Salmonella and a 3-D Biomimetic Intestinal Co-Culture Model
Source: Front Cell Infect Microbiol. 2022 May 31;12:705647. doi: 10.3389/fcimb.2022.705647 (PMC9195300; doi:10.3389/fcimb.2022.705647)
Supplement: Supplementary file 6 [file Table_1.pdf]

Supplementary Table 1. LSMMG-regulated genes for wild type *S.* Typhimurium relative to control cultures*

Gene	Fold Change	Description
**SPI-1**		
prgI	4.508	EscF/YscF/HrpA family type III secretion system needle major subunit
sipC	3.776	Pathogenicity island 1 effector protein
sipB	5.502	Pathogenicity island 1 effector protein
sicA	4.306	CesD/SycD/LcrH family type III secretion system chaperone
invA	3.701	EscV/YscV/HrcV family type III secretion system export apparatus protein
invG	6.662	EscC/YscC/HrcC family type III secretion system outer membrane ring protein
invF	7.976	Invasion protein
sitA	-3.745	Iron ABC transporter substrate-binding protein
sitB	-4.450	Manganese/iron transporter ATP-binding protein
sitC	-4.971	Iron ABC transporter permease
sitD	-6.206	Iron ABC transporter permease
iagB	-3.796	Invasion protein IagB
spaR	-4.689	EscT/YscT/HrcT family type III secretion system export apparatus
**SPI-2**		
sifA	-3.947	Effector protein SifA
sifB	-9.792	Effector protein SifB
ssaL	-4.582	secretion system apparatus protein
ssaM	-11.888	type III secretion system protein SsaM
ssaV	-4.448	EscV/YscV/HrcV family type III secretion system export apparatus protein
sseJ	-5.512	Pathogenicity island 2 effector SseJ
STM1698	-5.016	Secreted effector kinase SteC
sseL	-5.239	Effector protein SseL; Deubiquitinase for macrophage killing and virulence
**SPI-4**		
STM4262	-9.845	Antibiotic ABC transporter ATP-binding protein, SiiF
**SPI-5**		
sigE	5.650	Class IA chaperone involved in stability, secretion, and translocation of SopB
sopB	4.965	Inositol phosphatase
**Plasmid**		
parB	3.110	Plasmid partition protein B
PSLT034	-4.650	Putative adhesin
spvD	-14.285	putative transposase
spvB	-8.355	Salmonella plasmid virulence: hydrophilic protein
spvR	-8.004	*Salmonella* plasmid virulence: regulation of spv operon, lysR family
tlpA	-6.472	Alpha-helical coiled coil protein
PSLT049	-7.424	Putative DNA polymerase III epsilon subunit (3'-5' exonuclease)
PSLT050.1N	-12.599	
PSLT050.2N	-8.624	
traJ	-6.796	Conjugative transfer: regulation
traD	-5.250	Conjugative transfer: DNA transport

Gene	Fold Change	Description
**Motility and chemotaxis**		
flgA	7.531	Flagella basal body P-ring formation protein FlgA
flgB	15.541	Flagellar basal body rod protein FlgB
flgC	10.086	Flagellar basal body rod protein FlgC
flgD	25.596	Flagellar basal body rod modification protein
flgE	16.166	Flagellar hook protein FlgE
flgF	8.133	Flagellar biosynthesis protein FlgF
flgG	5.626	Flagellar basal-body rod protein FlgG
flgI	5.608	Flagellar biosynthesis protein FlgA
flgJ	5.518	Flagellar rod assembly protein/muramidase FlgJ
flgL	3.908	Flagellar hook-filament junction protein FlgL
motB	3.393	Flagellar motor protein MotB
motA	3.556	Flagellar motor stator protein MotA
fliZ	6.106	Flagellar regulatory protein FliZ
fliA	5.428	RNA polymerase sigma factor FliA
fliC	4.098	Flagellin
fliD	6.044	Flagellar filament-capping protein FliD
fliS	6.622	Flagellar export chaperone FliS
fliT	5.373	Flagellar biosynthesis protein FliT
fliF	5.605	Flagellar M-ring protein FliF
fliJ	12.874	Flagellar biosynthesis chaperone FliJ
fliL	7.342	Flagellar basal body-associated protein FliL
fliM	7.345	Flagellar motor switch protein FliM
fliN	18.490	Flagellar motor switch protein FliN
cheZ	4.474	Protein phosphatase CheZ
cheY	4.428	Two-component system response regulator
cheB	4.425	Chemotaxis response regulator protein-glutamate methylesterase
cheM	3.932	Methyl-accepting chemotaxis protein II
cheW	2.881	Chemotaxis protein CheW
cheA	3.605	Sensory histidine protein kinase
tcp	5.305	Methyl-accepting chemotaxis protein II
aer	2.800	Aerotaxis receptor
STM3152	-8.083	McpB; putative methyl-accepting chemotaxis protein
ymdF	-9.904	Involved in flagella-dependent motility
yciG	-3.600	Involved in flagella-dependent motility
**Transport**		
gltL	3.584	Arginine transporter ATP-binding subunit
nagE	2.680	PTS N-acetyl glucosamine transporter subunit IIABC
cydC	2.672	Cysteine/glutathione ABC transporter ATP-binding protein/permease CydC
STM1613	2.973	PTS sugar transporter subunit IIB

Fimbrial proteins/Adhesins		
fimA	6.086	Type-1 fimbrial protein subunit A
fimC	5.006	Fimbrial chaperone protein FimC
fimZ	3.142	DNA-binding response regulator
bcfA	-3.734	Fimbrial protein
stiB	-26.512	Long polar fimbrial chaperone LpfB
safB	-3.780	Putative fimbriae assembly chaperone
lpfA	-19.085	Long polar fimbria
stjB	-3.677	Fimbrial assembly protein
stjC	-5.093	Fimbrial chaperone protein
STM4593	-12.124	Putative fimbrial usher protein
STM4594	-14.483	Fimbrial assembly chaperone SthB
STM4595	-11.698	Fimbrial protein SthA
STM0551	-11.770	Diguanylate cyclase
stcD	-6.945	Adhesin
stcB	-12.995	Putative fimbrial chaperone protein
**Transcriptional regulators**		
araC	3.169	DNA-binding transcriptional regulator AraC
STM2361	3.083	Sigma-54-dependent Fis family transcriptional regulator
STM3124	2.857	Helix-turn-helix transcriptional regulator
STM3678	3.522	AraC family transcriptional regulator
melR	3.162	Transcriptional regulator MelR
STM4315	8.265	AraC family transcriptional regulator
STM0029	-83.659	Transcriptional regulator
sinR	-3.205	Transcriptional regulator
marR	-3.775	Transcriptional regulator
rcsA	-4.100	Helix-turn-helix transcriptional regulator
ygaE	-5.758	Transcriptional regulator
STM2797	-6.135	Transcriptional regulator
STM3098	-3.628	Putative transcriptional regulator
envR	-42.880	acrEF/envCD operon transcriptional regulator
rmbA	-33.603	Transcriptional regulator
**Metabolism**		
sdhC	3.522	Succinate dehydrogenase, cytochrome b556
sdhD	3.563	Succinate dehydrogenase, hydrophobic membrane anchor protein
sdhA	5.231	Succinate dehydrogenase flavoprotein subunit
sdhB	4.949	Succinate dehydrogenase, Fe-S protein
sucA	4.243	2-oxoglutarate dehydrogenase E1 component
sucB	3.706	Dihydrolipoamide succinyltransferase
sucD	3.059	Succinate--CoA ligase subunit alpha
cydB	3.490	Cytochrome d ubiquinol oxidase subunit II
prpR	3.667	Propionate catabolism operon regulatory protein PrpR
ushA	2.925	Bifunctional UDP-sugar hydrolase/5'-nucleotidase
cstA	3.498	Carbon starvation protein A

Transport (continued)		
mglB	2.879	Methyl-galactoside ABC transporter substrate-binding protein
glpT	3.882	Glycerol-3-phosphate transporter
cysA	10.840	Sulfate ABC transporter ATP-binding protein
cysW	8.936	Sulfate ABC transporter permease subunit CysW
cysP	7.737	Thiosulfate transporter subunit
STM3134	2.780	MFS transporter
STM3169	4.314	C4-dicarboxylate ABC transporter substrate-binding protein
STM3170	3.518	C4-dicarboxylate ABC transporter permease
nanT	3.966	MFS transporter
dgoT	7.721	MFS transporter
rbsC	3.139	ribose ABC transporter permease
STM4065	11.036	MFS transporter
putA	4.491	Trifunctional transcriptional regulator/proline dehydrogenase
putP	2.979	Sodium/proline symporter
glpF	3.280	Aquaporin
STM0520	-15.981	MFS transporter
allP	-81.265	Putative NCS1 family, allantoin transport protein
ybbY	-4.277	Uracil/xanthine transporter
STM0718	-22.290	Transport protein
STM0722	-16.337	ABC transporter permease
STM0723	-4.223	Sugar ABC transporter ATP-binding protein
STM0765	-15.373	Putative cation transporter
ybiR	-4.716	Anion transporter
ulaA	-8.690	PTS system ascorbate-specific transporter subunit IIC
macA	-2.858	Macrolide transporter subunit MacA
STM1128	-3.315	Acetylneuraminate ABC transporter
potC	-3.645	Spermidine/putrescine ABC transporter permease PotC
STM1256	-4.197	Peptide ABC transporter permease
ydiM	-5.436	MFS transporter
STM1634	-3.491	Amino acid ABC transporter ATP-binding protein
STM1668	-8.566	ZirS protein, secreted by zirT
STM1669	-8.730	ZirT, outer membrane protein, secretes zirS; homology to invasin C of Yersinia
narK	-8.501	Nitrate/nitrite transporter
pagO	-20.129	EamA family transporter
znuB	-2.870	Zinc ABC transporter permease
tyrP	-7.342	Tyrosine transporter TyrP
yedA	-3.994	Drug/metabolite exporter YedA
setB	-13.870	Sugar efflux transporter SetB
yejE	-4.099	Microcin ABC transporter permease
yejF	-2.967	Microcin C ABC transporter ATP-binding protein YejF
yojI	-3.619	Multidrug ABC transporter permease/ATP-binding protein
STM2274	-8.369	MFS transporter
xapB	-6.945	MFS transporter
sinH	-8.029	Intimin-like inverse autotransporter protein
STM2574	-10.500	MFS transporter

Metabolism (continued)		
rihA	2.598	Pyrimidine-specific ribonucleoside hydrolase RihA
hutH	2.770	Histidine ammonia-lyase
bioC	3.482	Malonyl-ACP O-methyltransferase BioC
dmsA	4.412	Dimethylsulfoxide reductase subunit A
dmsB	5.643	Dimethylsulfoxide reductase, chain B
dmsC	6.421	Dimethyl sulfoxide reductase
hpaE	3.643	5-carboxymethyl-2-hydroxymuconate semialdehyde dehydrogenase
hpaD	3.305	3,4-dihydroxyphenylacetate 2,3-dioxygenase
hpaF	2.744	5-carboxymethyl-2-hydroxymuconate delta-isomerase
selD	3.168	Selenophosphate synthase
nemA	2.711	N-ethylmaleimide reductase
fumA	3.819	Fumarate hydratase
rspB	3.575	Zn-dependent oxidoreductase
pykA	3.114	Pyruvate kinase
phsA	3.298	Thiosulfate reductase PhsA
yeiA	3.309	Dihydropyrimidine dehydrogenase subunit B
glpQ	3.504	Glycerophosphodiester phosphodiesterase
glpA	3.516	sn-glycerol-3-phosphate dehydrogenase subunit A
glpB	4.781	Anaerobic glycerol-3-phosphate dehydrogenase subunit B
glpC	3.625	sn-glycerol-3-phosphate dehydrogenase subunit C
fabB	2.929	Beta-ketoacyl-[acyl-carrier-protein] synthase I
cysK	3.215	Cysteine synthase A
hypB	3.448	Hydrogenase accessory protein HypB
STM2914	8.327	Putative nucleoside-diphosphate-sugar epimerase
cysN	10.282	Sulfate adenylyltransferase subunit CysN
cysD	10.187	Sulfate adenylyltransferase small subunit
cysH	6.869	Phosphoadenosine phosphosulfate reductase
cysI	4.905	Sulfite reductase subunit beta
STM3082	5.627	Galactonate oxidoreductase
STM3136	5.367	Fructuronate reductase
STM3137	4.627	Glucuronate isomerase
nanK	4.152	N-acetylmannosamine kinase
STM3697	3.906	Mandelate racemase/muconate lactonizing protein
dgoA	10.879	2-oxo-3-deoxygalactonate 6-phosphate aldolase
dgoK	5.456	2-oxo-3-deoxygalactonate kinase
glnA	3.310	Type I glutamate--ammonia ligase
fdoH	2.859	Formate dehydrogenase subunit beta
fdoG	3.071	Formate dehydrogenase
STM4066	13.452	Aminoimidazole riboside kinase
STM4067	8.502	Putative ADP-ribosylglycohydrolase
glpK	3.232	Glycerol kinase
aceB	5.166	Malate synthase A
aceA	6.004	Isocitrate lyase
aceK	4.136	Bifunctional isocitrate dehydrogenase kinase/phosphatase
yjhP	2.926	Putative SAM-dependent methyltransferase
araD	-11.692	L-ribulose-5-phosphate 4-epimerase
STM0018	-8.786	Chitinase
STM0019	-3.682	Chitinase

Transport (continued)		
STM2752	-13.745	PTS sorbitol transporter subunit IIB
nixA	-6.989	High-affinity nickel-transport protein, NixA
STM3022	-5.009	Transporter
STM3771	-6.221	PTS sugar transporter subunit IIB
pstS	-6.189	Phosphate ABC transporter substrate-binding protein PstS
yigM	-5.799	EamA family transporter
ompL	-13.351	Outer membrane porin L
yabF	-56.706	glutathione-regulated potassium-efflux system ancillary protein KefF
STM4206	-7.260	translocase
yjeM	-7.303	Glutamate/gamma-aminobutyrate family transporter YjeM
yifZ	-3.884	EamA family transporter
**Hypothetical, unknown function and pseudogenes**		
STM0989	3.616	Hypothetical protein
ygbK	3.962	Hypothetical protein
STM3604	4.505	Hypothetical protein
STM04675	16.686	
yegU	3.263	Hypothetical protein
hutU	2.846	Pseudogene
ygbJ	3.704	3-hydroxyisobutyrate dehydrogenase
fucP	3.283	Pseudogene
STM3828.1N	6.636	
STM4305.S	3.040	
STM0020	-5.359	Hypothetical protein
STM0034	-59.511	Hypothetical protein
STM0305	-10.623	Hypothetical protein
STM0348	-14.671	Hypothetical protein
STM0373	-3.008	yaiU; Similar to 3rd module of ATP-binding components of transporters
ylaC	-3.750	Hypothetical protein
STM0777	-5.300	Hypothetical protein
STM0719	-151.463	putative UDP-galactopyranose mutase
STM0726	-37.734	Putative glycosyl transferase
STM0759	-4.617	Uncharacterized protein ybgS
ybhM	-38.365	Hypothetical protein
STM0839	-5.720	putative inner membrane protein
STM0860	-7.203	Hypothetical protein
ycdF	-10.756	pseudogene
STM1131	-8.775	Hypothetical protein
ydhI	-5.845	Hypothetical protein
ynfM	-5.384	Hypothetical protein
STM1379	-3.576	Putative amino acid permease
STM1380	-4.504	Putative hydrolase or acyltransferase
STM1552	-3.395	putative cytoplasmic protein
STM1553	-8.088	pseudogene
STM1637	-4.423	Hypothetical protein
STM1665	-6.556	Hypothetical protein
STM1666	-5.941	pseudo
STM1671	-9.440	putative bacterial regulatory helix-turn-helix protein, araC family
STM1863	-4.692	putative inner membrane protein

**Metabolism (continued)**		
STM0330	-10.818	3-isopropylmalate dehydratase small subunit
STM0361	-4.488	Cytochrome BD2 subunit II
STM0855	-3.253	Putative electron transfer flavoprotein beta subunit
STM0856	-10.576	Electron transfer flavoprotein subunit alpha
STM1002	-5.423	Diaminopropionate ammonia-lyase
STM1269	-8.294	Putative chorismate mutase
celF	-8.047	6-phospho-beta-glucosidase
STM1620	-6.441	lactate oxidase
udg	-5.249	UDP-glucose 6-dehydrogenase
wcaF	-53.368	Colanic acid biosynthesis acetyltransferase WcaF
STM05445	-18.670	Purine nucleoside phosphorylase
eutE	-5.686	Aldehyde dehydrogenase EutE
eutQ	-25.510	Ethanolamine utilization protein EutQ
yfiN	-4.165	Diguanylate cyclase
STM3123	-4.760	Anaerobic sulfatase maturase
STM3532	-4.183	Dihydrodipicolinate synthase family protein
yibD	-66.592	Putative glycosyltransferase
STM4205	-3.610	Glycosyltransferase
STM4433	-4.250	Inositol 2-dehydrogenase
STM4467	-8.310	Putative arginine deiminase
**Other functions**		
yfiA	3.528	Ribosomal subunit interface protein
STM2693	3.489	tmRNA, 10Sa RNA, ssrA
STM0084	-9.981	AslA sulfatase
polB	-3.152	DNA polymerase II
STM0291	-3.817	Putative RHS-family protein
STM0294	-14.389	Phosphotriesterase
STM0295	-18.732	Putative cytoplasmic protein
STM0721	-16.973	Glycosyltransferase family 1 protein
STM0724	-24.169	Glycosyl transferase
STM0725	-13.694	Glycosyl transferase
STM0854	-7.482	CoA ester lyase
ybeV	-9.211	Molecular chaperone DnaJ
pagC	-5.866	Virulence membrane protein PagC
STM1267	-3.849	Histidine kinase
marB	-5.510	Multiple antibiotic resistance regulatory periplasmic protein MarB
STM1530	-3.655	phosphoporin PhoE
STM1836	-3.714	Peptidoglycan synthase, penicillin-binding protein 3
pphA	-3.742	Phosphoprotein phosphatase
umuD	-4.279	DNA polymerase V subunit UmuD
engA	-4.930	Ribosome biogenesis GTPase Der
pphB	-17.053	Serine/threonine protein phosphatase
rpsU	-26.663	30S ribosomal protein S21
hopD	-3.725	Prepilin peptidase
yrfD	-22.854	DNA utilization protein HofM
dinF	-3.303	DNA-damage-inducible protein F

**Hypothetical, unknown function and pseudogenes (continued)**		
STM1866	-4.565	pseudo
STM1896	-8.206	putative cytoplasmic protein
STM1999	-7.247	Hypothetical protein
STM2008	-3.013	Hypothetical protein
STM2137	-5.298	putative cytoplasmic protein
STM2156A	-4.139	Hypothetical protein
STM2208	-10.759	Hypothetical protein
STM2245	-7.368	putative outer membrane protein
STM2508	-21.030	putative cytoplasmic protein
STM2509	-7.155	Hypothetical protein
yfgJ	-5.463	Hypothetical protein
STM2534	-4.594	DUF5066 domain-containing protein
STM2906	-7.104	putative cytoplasmic protein
STM2908	-18.953	Hypothetical protein
STM2954.1n	-3.379	Hypothetical protein
STM3026	-13.558	Hypothetical protein
STM3035	-43.269	Hypothetical protein
yghW	-4.934	Hypothetical protein
STM3153	-3.546	Hypothetical protein
STM3166.S	-25.720	putative cation transporter
yhbE	-3.296	pseudogene
STM3906	-4.579	Hypothetical protein
STM3907	-3.685	Hypothetical protein
STM3941	-127.454	Hypothetical protein
STM3942	-7.917	Hypothetical protein
STM4013.S	-4.456	
STM4014	-39.694	Putative periplasmic protein
STM4015	-4.020	Hypothetical protein
STM4039	-5.528	Hypothetical protein
yiiG	-6.952	putative cytoplasmic protein
STM4197	-17.380	putative inner membrane protein
STM4219.S	-3.984	
yjcB	-6.270	Hypothetical protein
yjeJ	-4.361	Hypothetical protein
STM4472	-5.140	Hypothetical protein
STM4504	-8.830	Hypothetical protein
STM4575	-7.204	Hypothetical protein
**Phage/Prophage proteins**		
STM0908	-6.070	Fels-1 prophage protein
mig-3	-6.254	Phage tail protein
STM2585	-16.099	SarA/PagJ, *Salmonella*-anti-inflammatory response activator; Gifsy-1 prophage protein
STM2585A	-56.599	Gifsy-1 prophage protein, PagK2
STM2586	-35.924	Gifsy-1 prophage protein
STM2587	-72.380	Gifsy-1 prophage protein
STM2608	-3.747	Gifsy-1 prophage protein
STM2620	-4.292	Gifsy-1 prophage protein
STM2702	-40.059	Fels-2 prophage protein
STM2722	-21.560	Fels-2 prophage protein

* Significant differences between the LSMMG and control cultures were determined according to an FDR < 0.05 and a minimum logFC of 1 or -1 (corresponding to a 2-fold increase or decrease in expression, respectively). LogFC values were converted to fold change. Red shading indicates upregulation in the LSMMG culture, blue shading downregulation in the LSMMG culture.
