# Supplementary material for: Spaceflight Analogue Culture Enhances the Host-Pathogen Interaction Between Salmonella and a 3-D Biomimetic Intestinal Co-Culture Model
Source: Front Cell Infect Microbiol. 2022 May 31;12:705647. doi: 10.3389/fcimb.2022.705647 (PMC9195300; doi:10.3389/fcimb.2022.705647)
Supplement: Supplementary file 7 [file Table_2.pdf]

Supplementary Table 2. Bacterial GO Biological Process and KEGG pathway enrichment analysis[1]

Category	Term	Count	Percent	P-value	Fold Enrichment	Benjamini
**Wild Type: LSMMG versus control**						
GO Biological Process						
GOTERM_BP_DIRECT	Chemotaxis	15	4.5	8.20E-10	7.4	1.40E-07
GOTERM_BP_DIRECT	Pathogenesis	21	6.2	1.40E-06	3.4	1.20E-04
GOTERM_BP_DIRECT	Bacterial-type flagellum-dependent swarming motility	7	2.1	5.10E-06	11.2	1.70E-04
GOTERM_BP_DIRECT	Tricarboxylic acid (TCA) cycle	11	3.3	3.10E-06	6.1	1.70E-04
GOTERM_BP_DIRECT	Bacterial-type flagellum-dependent cell motility	10	3	4.10E-06	6.7	1.70E-04
GOTERM_BP_DIRECT	Cell adhesion	10	3	5.10E-04	4	1.40E-02
GOTERM_BP_DIRECT	Pilus organization	6	1.8	2.10E-03	5.9	4.80E-02
KEGG Pathway						
KEGG_PATHWAY	Flagellar assembly	19	5.6	2.80E-11	6.4	1.80E-09
KEGG_PATHWAY	Bacterial chemotaxis	14	4.2	1.80E-09	7.6	5.60E-08
KEGG_PATHWAY	Sulfur metabolism	13	3.9	1.90E-05	4.2	3.90E-04
KEGG_PATHWAY	TCA cycle	8	2.4	3.20E-03	3.8	4.00E-02
KEGG_PATHWAY	Microbial metabolism in diverse environments	32	9.5	3.10E-03	1.6	4.80E-02
**Δ*hfq:* LSMMG versus control**						
GO Biological Process						
GOTERM_BP_DIRECT	Pathogenesis	37	7.9	6.80E-14	3.8	1.60E-11
GOTERM_BP_DIRECT	Chemotaxis	14	3	2.10E-06	4.5	2.60E-04
GOTERM_BP_DIRECT	Bacterial-type flagellum-dependent cell motility	11	2.3	1.90E-05	4.8	1.50E-03
GOTERM_BP_DIRECT	Bacterial-type flagellum-dependent swarming motility	7	1.5	6.60E-05	7.2	3.90E-03
KEGG Pathway						
KEGG_PATHWAY	Flagellar assembly	24	5.1	2.60E-13	5.4	2.20E-11
KEGG_PATHWAY	Bacterial chemotaxis	14	3	2.70E-07	5.1	1.20E-05
KEGG_PATHWAY	Sulfur metabolism	15	3.2	5.60E-05	3.3	1.60E-03
KEGG_PATHWAY	*Salmonella* infection	12	2.6	8.40E-05	3.8	1.80E-03
KEGG_PATHWAY	Bacterial invasion of epithelial cells	6	1.3	2.00E-03	5.5	3.30E-02
**LSMMG cultures only: Δ*hfq* versus Wild type**						
GO Biological Process						
GOTERM_BP_DIRECT	Pathogenesis	28	7.4	3.80E-10	3.8	8.20E-08
GOTERM_BP_DIRECT	Arginine biosynthetic process	7	1.8	1.70E-04	7	1.80E-02
KEGG Pathway						
KEGG_PATHWAY	Bacterial invasion of epithelial cells	6	1.6	7.30E-04	6.8	5.40E-02
**Control cultures only: Δ*hfq* versus Wild type**						
GO Biological Process						
GOTERM_BP_DIRECT	TCA cycle	13	2.6	6.40E-07	5.4	1.50E-04
KEGG Pathway						
KEGG_PATHWAY	TCA cycle	10	2	4.60E-04	3.9	3.80E-02

[*] GO Biological process and KEGG pathway enrichment analyses were performed using DAVID 6.8 using a threshold count of 2 and an EASE score of 0.05. Only terms and pathways with Benjamini-Hochberg values less than 0.05 are shown.
