# Supplementary material for: Spaceflight Analogue Culture Enhances the Host-Pathogen Interaction Between Salmonella and a 3-D Biomimetic Intestinal Co-Culture Model
Source: Front Cell Infect Microbiol. 2022 May 31;12:705647. doi: 10.3389/fcimb.2022.705647 (PMC9195300; doi:10.3389/fcimb.2022.705647)
Supplement: Supplementary file 8 [file Table_3.pdf]

Supplementary Table 3. LSMMG-regulated genes previously identified as regulated by RpoS*

Functional Category	Gene	Description	Fold Change WT (LSMMG/control)	Fold change Δ*hfq* (LSMMG/control)
**Metabolism**	*sdhA*	Succinate dehydrogenase flavoprotein subunit	5.2	4.3
	sdhB	Succinate dehydrogenase, Fe-S protein	4.9	3.4
	sdhC	Succinate dehydrogenase, cytochrome b556	3.5	5.0
	sdhD	Succinate dehydrogenase, hydrophobic membrane anchor protein	3.5	
	sucB	Dihydrolipoamide succinyltransferase	3.7	3.6
	sucD	Succinate—CoA ligase subunit alpha	3.0	2.7
	dgoA	2-oxo-3-deoxygalactonate 6-phosphate aldolase	10.8	
	dgoK	2-oxo-3-deoxygalactonate kinase	5.4	
	cysK	Cysteine synthase A	3.2	
	cysD	Sulfate adenylyl transferase small subunit	10.1	6.3
	cysN	Sulfate adenylyl transferase subunit CysN	10.2	4.9
	fumA	Fumarate hydratase	3.8	
	fdoH	Formate dehydrogenase subunit beta	2.8	
	fdoG	Formate dehydrogenase	3.0	
	hutH	Histidine ammonia-lyase	2.7	
	fumA	Fumarate hydratase	3.8	
	fdoH	Formate dehydrogenase subunit beta	2.8	
	fdoG	Formate dehydrogenase	3.0	
	hutH	Histidine ammonia-lyase	2.7	6.4
	hpaD	3,4-dihydroxyphenylacetate 2,3-dioxygenase	3.3	
	hpaF	5-carboxymethyl-2-hydroxymuconate delta-isomerase	2.7	
	hpaE	5-carboxymethyl-2-hydroxymuconate semialdehyde dehydrogenase	3.6	
	glnA	Type I glutamate—ammonia ligase	3.3	
	phsA	Thiosulfate reductase PhsA	3.2	
	nanK	N-acetylmannosamine kinase	4.1	
	rihA	Pyrimidine-specific ribonucleoside hydrolase RihA	2.5	
	ushA	Bifunctional UDP-sugar hydrolase/5'-nucleotidase	2.9	
	yeiA	Dihydropyrimidine dehydrogenase subunit B	3.3	
**SPI-1**	*invA*	EscV/YscV/HrcV family type III secretion system export apparatus protein	3.7	8.3
	invG	EscC/YscC/HrcC family type III secretion system outer membrane ring protein	6.6	33.6
	invF	Invasion protein	7.9	
	prgI	EscF/YscF/HrpA family type III secretion system needle major subunit	4.5	64.2
	sicA	CesD/SycD/LcrH family type III secretion system chaperone	4.3	
	sipB	Pathogenicity island 1 effector protein	5.5	40.5
	sipC	Pathogenicity island 1 effector protein	3.7	33.8
	spaR	EscT/YscT/HrcT family type III secretion system export apparatus protein	-4.6	
	iagB	Invasion protein IagB	-3.7	3.1
	sitA	Iron ABC transporter substrate-binding protein	-3.7	
	sitB	Manganese/iron transporter ATP-binding protein	-4.4	-7.8
	sitC	Iron ABC transporter permease	-4.9	
	sitD	Iron ABC transporter permease	-6.2	
**SPI-2**	*ssaM*	type III secretion system protein SsaM	-11.8	

	ssaV	EscV/YscV/HrcV family type III secretion system export apparatus protein	-4.4	
	sifB	Effector protein SifB	-9.7	-5.8
**Plasmid**	*spvB*	*Salmonella* plasmid virulence: hydrophilic protein	-8.3	
	spvD	putative transposase	-14.2	-14.3
	spvR	*Salmonella* plasmid virulence: regulation of spv operon, lysR family	-8.0	
**Motility and Chemotaxis**	*fliC*	Flagellin	4.0	16.4
	motA	Flagellar motor stator protein MotA	3.5	4.3
	cheM	Methyl-accepting chemotaxis protein II	3.9	5.3
	tcp	Methyl-accepting chemotaxis protein II	5.3	
	cheA	Sensory histidine protein kinase	3.6	21.1
	yciG	Involved in flagella-dependent motility	-3.6	
	ymdF	Involved in flagella-dependent motility	-9.9	
**Fimbriae**	*fimA*	Type-1 fimbrial protein subunit A	6.0	4.5
	fimC	Fimbrial chaperone protein FimC	5.0	
	bcfA	Fimbrial protein	-3.7	
	stiB	Long polar fimbrial chaperone LpfB	-26.5	
**Transport**	*cydC*	Cysteine/glutathione ABC transporter ATP-binding protein/permease CydC	2.6	
	nanT	MFS transporter	3.9	
	tyrP	Tyrosine transporter TyrP	-7.3	-6.4
	znuB	Zinc ABC transporter permease	-2.8	
	yabF	glutathione-regulated potassium-efflux system ancillary protein KefF	-56.7	

* Significant differences between the LSMMG and control cultures were determined according to an FDR < 0.05 and a minimum logFC of 1 or -1 (corresponding to a 2-fold increase or decrease in expression, respectively). LogFC values were converted to fold change. Differentially expressed genes were searched against published literature to determine whether they were previously identified as RpoS-regulated.
