# Supplementary material for: Spaceflight Analogue Culture Enhances the Host-Pathogen Interaction Between Salmonella and a 3-D Biomimetic Intestinal Co-Culture Model
Source: Front Cell Infect Microbiol. 2022 May 31;12:705647. doi: 10.3389/fcimb.2022.705647 (PMC9195300; doi:10.3389/fcimb.2022.705647)
Supplement: Supplementary file 9 [file Table_4.pdf]

Supplementary Table 4. LSMMG-regulated genes for Δ*hfq* *S*. Typhimurium relative to control cultures*

Gene	Fold Change	Description
**SPI-1 associated genes, effectors and regulators**		
sirC	15.508	Transcriptional regulator SirC (HilC)
orgA	14.361	Invasion protein OrgA
prgK	20.202	EscJ/YscJ/HrcJ family type III secretion inner membrane ring protein
prgJ	20.744	Type III secretion system protein PrgJ
prgI	64.243	EscF/YscF/HrpA family type III secretion system needle major subunit
prgH	17.648	Type III secretion system protein PrgH
hilD	6.240	AraC family transcriptional regulator
hilA	21.485	Transcriptional regulator
iagB	3.142	Invasion protein IagB
sicP	5.082	Chaperone protein SicP
STM2880	3.552	Putative cytoplasmic protein
iacP	37.730	Putative acyl carrier protein
sipA	42.336	Pathogenicity island 1 effector protein SipA
sipD	27.467	Cell invasion protein SipD
sipC	33.849	Pathogenicity island 1 effector protein SipC
sipB	40.538	Pathogenicity island 1 effector protein SipB
sicA	53.027	CesD/SycD/LcrH family type III secretion system chaperone
spaO	25.536	Type III secretion system protein SpaO
spaN	30.346	
invI	179.644	Type III secretion system protein SpaM
invC	22.704	EscN/YscN/HrcN family type III secretion system ATPase
invB	9.888	Type III secretion system chaperone SpaK
invA	8.325	EscV/YscV/HrcV family type III secretion system export apparatus protein
invE	9.980	SepL/TyeA/HrpJ family type III secretion system gatekeeper
invG	33.581	EscC/YscC/HrcC family type III secretion system outer membrane ring protein
sopE2	12.178	Type III secretion protein SopE2
sopA	9.372	Similar to secreted effector protein of Salmonella dublin
STM1239	10.578	Effector protein SopF
rtsB	16.456	Regulatory protein, LuxR family, RtsB
rtsA	14.905	Regulatory protein, AraC family, RtsA
sitB	-7.836	Manganese/iron transporter ATP-binding protein
STM0972	-3.720	Pathogenicity island 1 protein SopD2
**SPI-2 associated genes and effectors**		
ssaB	-9.762	Pathogenicity island chaperone protein SpiC
sseA	-5.093	3-mercaptopyruvate sulfurtransferase
sseB	-4.363	Enhances serine sensitivity
ssaK	-172.804	Type III secretion system protein SsaK

Gene	Fold Change	Description
**Motility and chemotaxis**		
flgN	4.572	Flagellar biosynthesis protein FlgN
flgM	9.984	Anti-sigma-28 factor FlgM
flgA	17.345	Flagella basal body P-ring formation protein FlgA
flgB	9.426	Flagellar basal body rod protein FlgB
flgC	26.457	Flagellar basal body rod protein FlgC
flgD	42.056	Flagellar basal body rod modification protein
flgE	57.453	Flagellar hook protein FlgE
flgF	8.121	Flagellar biosynthesis protein FlgF
flgG	587.442	Flagellar basal-body rod protein FlgG
flgH	46.693	Flagellar basal body L-ring protein
flgI	30.859	Flagellar biosynthesis protein FlgA
flgK	6.019	Flagellar hook-associated protein FlgK
flgL	44.485	Flagellar hook-filament junction protein FlgL
motB	4.556	Flagellar motor protein MotB
motA	4.329	Flagellar motor stator protein MotA
fliZ	45.991	Flagellar regulatory protein FliZ
fliA	35.282	RNA polymerase sigma factor FliA
fliB	22.809	Lysine-N-methylase
fliC	16.368	Flagellin
fliD	16.614	Flagellar filament-capping protein FliD
fliS	7.675	Flagellar export chaperone FliS
fliT	8.448	Flagellar biosynthesis protein FliT
fliF	9.090	Flagellar M-ring protein FliF
fliK	30.814	Flagellar hook-length control protein FliK
fliL	135.286	Flagellar basal body-associated protein FliL
fliM	12.544	Flagellar motor switch protein FliM
fljB	11.695	Flagellin
cheY	11.463	Two-component system response regulator
cheB	6.875	Chemotaxis response regulator protein-glutamate methylesterase
cheR	12.998	Chemotaxis protein-glutamate O-methyltransferase
cheM	5.477	Methyl-accepting chemotaxis protein II
cheW	185.948	Chemotaxis protein CheW
cheA	21.097	Sensory histitine protein kinase
trg	17.168	Methyl-accepting chemotaxis protein
STM3138	13.976	Chemotaxis protein
STM3216	26.863	Putative methyl-accepting chemotaxis protein
aer	3.696	Aerotaxis receptor
tsr	7.639	Methyl-accepting chemotaxis protein II

**SPI-2 associated genes and effectors (continued)**		
ssaU	-8.045	EscU/YscU/HrcU family type III secretion system export apparatus switch protein
pipB2	-3.464	Effector protein PipB2
sifA	-4.726	Effector protein SifA
sifB	-5.782	Effector protein SifB
**SPI-4**		
STM4257	18.542	SiiA protein
STM4258	15.550	SiiB protein
STM4259	11.106	ABC transporter, SiiC
STM4260	16.486	Cation transporter, SiiD
STM4261	8.437	SiiE protein
**SPI-5**		
STM1089	185.859	Pathogenicity island encoded protein: SPI5
copS	21.667	Two-component sensor histidine kinase
sigE	40.469	Class IA chaperone involved in stability, secretion, and translocation of SopB
sopB	33.714	Inositol phosphatase
pipA	-5.355	Virulence protein
**Plasmid**		
orf7	-17.867	Putative bacterial regulatory proteins, luxR family
pefI	-16.280	Plasmid-encoded fimbriae; regulatory
orf6	-3.704	Putative outer membrane protein
pefB	-28.354	Plasmid-encoded fimbriae; regulation
PSLT020	-18.842	hypothetical protein
PSLT034	-5.593	Putative adhesin
spvD	-14.295	Putative transposase
spvC	-17.546	*Salmonella* plasmid virulence: hydrophilic protein
spvB	-3.462	*Salmonella* plasmid virulence: hydrophilic protein
spvA	-8.209	Salmonella plasmid virulence: outer membrane protein
tlpA	-4.031	alpha-helical coiled coil protein
PSLT049	-5.798	putative DNA polymerase III epsilon subunit (3'-5' exonuclease)
PSLT050.1N	-23.600	
samB	-4.421	mutagenesis by UV and mutagens; related to umuDC operon
PSLT072	-8.900	putative transglycosylase
traN	-12.823	Conjugative transfer: aggregate stability
traF	-10.593	Conjugative transfer: assembly
traQ	-27.418	Conjugative transfer: fimbrial synthesis
trbB	-10.160	Conjugative transfer
trbH	-4.371	Conjugative transfer
PSLT106	-5.188	Homologue of mvpA, Shigella flexneri
traI	-18.693	Conjugative transfer: oriT nicking-unwinding
**Fimbrial proteins/Adhesins**		
fimA	4.535	Type-1 fimbrial protein subunit A

**Motility and chemotaxis (continued)**		
STM3156	6.036	Hypothetical protein, putative class 2 motility gene
STM3604	5.217	Hypothetical protein, putative class 2 motility gene
fliH	-9.909	Flagellar assembly protein FliH
**Transport**		
modB	84.783	Molybdate ABC transporter permease
putA	4.316	Trifunctional transcriptional regulator, proline dehydrogenase/L-glutamate gamma-semialdehyde dehydrogenase
STM1132	6.712	MFS transporter
ydjN	4.047	L-cystine transporter
oppB	3.643	Oligopeptide transporter permease
cysA	27.377	Sulfate ABC transporter ATP-binding protein
cysU	10.561	Sulfate ABC transporter permease subunit CysT
cysP	8.233	Thiosulfate transporter subunit
kgtP	10.803	Alpha-ketoglutarate transporter
srlE	14.246	PTS glucitol/sorbitol transporter subunit IIB
sdaC	3.707	HAAAP family serine/threonine permease
yheS	10.748	ABC transporter ATP-binding protein
mtlA	5.694	PTS mannitol transporter subunit IICBA
lldP	21.309	L-lactate permease
STM3782	4.592	PTS galactitol transporter subunit IIC
STM3792	14.926	L-fucose:H+ symporter permease
yidC	4.540	Membrane protein insertase YidC
trkD	6.921	Potassium transporter Kup
rbsC	3.633	Ribose ABC transporter permease
sbp	27.335	Sulfate transporter subunit
idnT	48.230	Gluconate permease
ybbY	-16.550	Uracil/xanthine transporter
STM0577	-10.421	PTS mannose transporter subunit IIAB
tbpA	-11.868	Thiamine ABC transporter substrate binding subunit
fhuA	-8.345	Ferrichrome porin FhuA
ybbM	-6.649	Iron export ABC transporter permease subunit FetB
fepA	-23.575	Outer membrane receptor protein
fepC	-66.456	Iron-enterobactin transporter ATP-binding protein
macA	-4.666	Macrolide transporter subunit MacA
yceL	-6.529	MFS transporter
potC	-3.281	Spermidine/putrescine ABC transporter permease PotC
potB	-3.946	Spermidine/putrescine ABC transporter permease PotB
STM1259	-50.542	Peptide ABC transporter ATP-binding protein
STM1260	-7.197	Mechanosensitive ion channel protein MscS
ydgF	-4.001	multidrug transporter subunit MdtJ
STM1491	-4.291	Glycine/betaine ABC transporter ATP-binding protein, OsmV

**Fimbrial proteins/Adhesins (continued)**		
fimI	9.670	Fimbrial protein FimI
fimD	4.180	Outer membrane usher protein
stdC	-73.692	Fimbrial chaperone protein StdC
lpfD	-4.538	Long polar fimbrial protein LpfD
STM4595	-10.280	Fimbrial protein SthA
STM0306	-3.682	Adhesin
**Transcriptional and translational regulators**		
yafC	5.226	Putative LysR family transcriptional regulator
phoB	5.676	Phosphate regulon transcriptional regulatory protein PhoB
STM0652	6.986	Fis family transcriptional regulator
STM0763.s	3.934	LysR family transcriptional regulator
STM1541	3.217	Putative gntR family regulatory protein
yebK	4.837	Transcriptional regulator HexR
STM2361	2.895	Sigma-54-dependent Fis family transcriptional regulator
STM3533	2.782	IclR family transcriptional regulator
STM3860	5.864	SgrR family transcriptional regulator
melR	3.189	Transcriptional regulator MelR
STM4308	59.783	Putative component of anaerobic dehydrogenases
STM0347	-7.195	Transcriptional regulator
ybdM	-6.778	Transcriptional regulator related to Sp0J
csgD	-80.518	Transcriptional regulator CsgD
marA	-4.642	AraC/XylS family transcriptional activator of defense systems
marR	-7.350	Transcriptional regulator
yciH	-3.599	Translation initiation factor
cysB	-5.522	Transcriptional regulator CysB
rcsA	-4.558	Helix-turn-helix transcriptional regulator
agaR	-11.012	DeoR family transcriptional regulator
STM3736	-9.740	Putative LysR family transcriptional regulator
STM3794	-11.808	DeoR family transcriptional regulator
ilvY	-7.190	Transcriptional regulator IlvY
**Metabolism**		
carB	5.356	Carbamoyl phosphate synthase large subunit
pdxA	5.335	4-hydroxythreonine-4-phosphate dehydrogenase PdxA
hemL	10.814	Glutamate-1-semialdehyde-2,1-aminomutase
prpR	12.734	Propionate catabolism operon regulatory protein PrpR
apbA	4.227	2-dehydropantoate 2-reductase
lnt	5.768	Apolipoprotein N-acyltransferase
pgm	3.184	Phosphoglucomutase, alpha-D-glucose phosphate-specific
sdhC	5.021	Succinate dehydrogenase, cytochrome b556
sdhA	4.293	Succinate dehydrogenase flavoprotein subunit
sdhB	3.414	Succinate dehydrogenase, Fe-S protein
sucB	3.636	Dihydrolipoamide succinyltransferase
sucD	2.717	Succinate--CoA ligase subunit alpha
cydB	2.615	Cytochrome d ubiquinol oxidase subunit II
hutH	6.413	Histidine ammonia-lyase
poxB	9.124	Pyruvate oxidase

**Transport (continued)**		
STM1492	-10.676	Choline ABC transporter permease, OsmW
STM1493	-5.449	Glycine/betaine ABC transporter substrate-binding protein, OsmX
STM1633	-6.061	D-alanine transporter, DalS
STM1669	-5.249	ZirS; homology to invasin C of Yersinia
chaA	-4.738	Sodium-potassium/proton antiporter ChaA
STM1843	-4.143	MFS transporter
pagO	-17.091	EamA family transporter
tyrP	-6.435	Tyrosine transporter TyrP
wza	-48.866	Polysaccharide exporter Wza
yegT	-3.617	MFS transporter
yehW	-4.527	ABC transporter permease
cirA	-8.685	Catecholate siderophore receptor CirA
yejE	-13.837	Microcin ABC transporter permease
yejF	-4.555	Microcin C ABC transporter ATP-binding protein YejF
ompC	-8.794	Porin OmpC
nupC	-9.846	Nucleoside permease
cysZ	-5.378	Sulfate transporter CysZ
nixA	-7.062	High-affinity nickel-transport protein, NixA
gabP	-4.467	GABA permease
yohM	-22.955	Nickel/cobalt efflux protein RcnA
acrE	-16.856	Multidrug exporter AcrE
bigA	-4.809	Putative surface-exposed virulence protein
yhgG	-70.870	Ferrous iron transporter C
yiaN	-16.300	L-dehydroascorbate transporter large permease subunit
emrD	-4.880	Multidrug transporter EmrD
dgoT	-6.624	MFS transporter
ompL	-6.551	Outer membrane porin L
yihO	-7.844	MFS transporter
malE	-3.536	Maltose transport protein
lamB	-5.828	Maltoporin
STM4267	-12.091	Putative glutathione S-transferase
STM4464	-10.699	Ion transporter superfamily protein
**Other functions**		
cysS	3.910	Cysteine tRNA synthetase
ybiB	4.669	DNA-binding protein YbiB
srfB	3.733	Virulence factor SrfB
srfC	9.482	Virulence factor SrfC
ynaF	4.396	Universal stress protein F
yciB	3.903	Septation protein A
asmA	5.091	Outer membrane assembly protein AsmA
yegD	11.935	Hsp70 chaperone family protein
rtn	3.942	Phage resistance protein
narQ	2.993	Two-component system sensor histidine kinase NarQ
ygiF	10.966	CYTH domain-containing protein
yhcM	6.575	Cell division protein ZapE
degS	15.323	Stress sensor serine endopeptidase DegS

Metabolism (continued)		
trxB	3.049	Thioredoxin-disulfide reductase
dmsA	6.712	Dimethylsulfoxide reductase subunit A
dmsC	5.146	Dimethyl sulfoxide reductase
lpxK	75.443	Tetraacyldisaccharide 4'-kinase
yeaD	4.774	D-hexose-6-phosphate mutarotase
sufB	-4.503	Fe-S cluster assembly protein SufB
sufC	-7.320	Fe-S cluster assembly ATPase SufC
sufD	-10.627	FeS cluster assembly protein SufD
ttrA	3.430	Tetrathionate reductase subunit A
ttrC	20.860	Tetrathionate reductase subunit C
ttrB	7.791	Tetrathionate reductase complex, subunit B
nemA	3.501	N-ethylmaleimide reductase
anmK	3.602	Anhydro-N-acetylmuramic acid kinase
STM1498	10.356	Dimethyl sulfoxide reductase subunit A
STM1532	54.358	ATP/GTP-binding protein
cybB	12.600	Cytochrome B
otsB	8.188	Trehalose-phosphatase
ftn	2.973	Ferritin
pgsA	6.234	CDP-diacylglycerol--glycerol-3-phosphate 3-phosphatidyltransferase
cbiB	12.451	Cobalamin biosynthesis protein CbiB
cbiA	12.680	Cobyrinic acid a,c-diamide synthase
hisI	4.730	Bifunctional phosphoribosyl-AMP cyclohydrolase/phosphoribosyl-ATP diphosphatase
fabB	6.090	beta-ketoacyl-[acyl-carrier-protein] synthase I
ucpA	4.078	NAD(P)-dependent oxidoreductase
alkB	87.357	alpha-ketoglutarate-dependent dioxygenase AlkB
glpA	3.317	sn-glycerol-3-phosphate dehydrogenase subunit A
glpB	3.194	Anaerobic glycerol-3-phosphate dehydrogenase subunit B
nuoB	23.938	NADH dehydrogenase
ispG	5.904	4-hydroxy-3-methylbut-2-en-1-yl diphosphate synthase
cysC	65.886	Adenylyl-sulfate kinase
cysN	4.861	Sulfate adenylyltransferase subunit CysN
cysD	6.295	Sulfate adenylyltransferase small subunit
cysH	39.282	Phosphoadenosine phosphosulfate reductase
cysI	14.670	Sulfite reductase subunit beta
sdaB	14.429	L-serine ammonia-lyase
argA	4.239	Amino-acid N-acetyltransferase
lysS	2.909	Lysine--tRNA ligase
gcvP	3.499	Glycine cleavage system protein P
gcvH	10.438	Glycine cleavage system protein H
visC	7.646	FAD-dependent 2-octaprenylphenol hydroxylase
ubiH	9.324	2-octaprenyl-6-methoxyphenyl hydroxylase
STM3082	3.305	Galactonate oxidoreductase
hybB	8.723	Ni/Fe-hydrogenase cytochrome b subunit
hypO	4.543	Hydrogenase 2 small subunit
ygjR	7.229	Oxidoreductase

Other functions (continued)		
sun	26.228	16S rRNA (cytosine(967)-C(5))-methyltransferase
uspA	4.139	Universal stress global response regulator UspA
yhiR	6.985	23S rRNA (adenine(2030)-N(6))-methyltransferase RlmJ
glyS	4.981	Glycine--tRNA ligase subunit beta
spoU	6.139	tRNA (guanosine(18)-2'-O)-methyltransferase TrmH
wecE	3.829	dTDP-4-amino-4,6-dideoxygalactose transaminase
hslU	3.548	HslU--HslV peptidase ATPase subunit
miaE	4.210	tRNA 2-methylthio-N6-isopentenyl adenosine(37) hydroxylase MiaE
STM4496	5.920	DNA repair protein
mrr	9.233	Restriction endonuclease
yjjI	3.394	YjjI family glycine radical enzyme
STM0084	-22.601	AslA sulfatase
htrA	-2.996	Serine endoprotease
STM0291	-7.390	Putative RHS-family protein
STM0294	-62.345	Phosphotriesterase
yafK	-8.224	Transpeptidase
ybaW	-6.730	Thioesterase
rna	-6.667	Ribonuclease I
pagP	-3.736	PhoPQ-activated gene
ybfE	-68.865	LexA regulated protein
ycfS	-5.711	L,D-transpeptidase
pliC	-3.808	Lysozyme inhibitor
aadA	-3.677	Aminoglycoside resistance protein
STM1267	-3.983	Histidine kinase
STM1330	-3.853	Endonuclease
marC	-3.216	Stress protection protein MarC
hdeB	-5.818	Acid stress chaperone HdeB
osmC	-7.910	OsmC family peroxiredoxin
yciL	-5.813	23S rRNA pseudouridylate synthase
rrmA	-13.705	23S rRNA (guanine(745)-N(1))-methyltransferase
cspB	-36.334	Putative cold-shock protein
umuC	-280.927	DNA polymerase V subunit UmuC
rsuA	-5.824	16S rRNA pseudouridine(516) synthase
yfgE	-5.162	DnaA regulatory inactivator Hda
STM2545	-2.717	tRNA (cytosine(32)/uridine(32)-2'-O)-methyltransferase TrmJ
STM2693	-3.429	tmRNA, 10Sa RNA, ssrA
STM2762	-11.685	Putative inner membrane protein
hin	-9.754	DNA-invertase
virK	-5.370	Similar to virK in Shigella
pphB	-11.413	Serine/threonine protein phosphatase
deaD	-3.914	Cysteine sulfinate desulfinase
nlpI	-4.195	Lipoprotein NlpI
STM3388	-4.888	Histidine kinase
STM3595	-3.149	Phosphoesterase PA-phosphatase

Metabolism (continued)		
argG	4.454	Argininosuccinate synthetase
pckA	3.328	Phosphoenolpyruvate carboxykinase (ATP)
selA	3.523	L-seryl-tRNA(Sec) selenium transferase
STM3793	27.598	Ribokinase
dnaN	12.131	DNA polymerase III subunit beta
rfaP	7.428	Lipopolysaccharide core heptose(I) kinase RfaP
pheA	3.418	Chorismate mutase
katG	3.803	Catalase/peroxidase HPI
gldA	5.692	Glycerol dehydrogenase
argE	4.104	Acetylornithine deacetylase
coaA	36.804	Type I pantothenate kinase
metH	3.672	B12-dependent homocysteine-N5-methyltetrahydrofolate transmethylase
nrfA	3.747	Ammonia-forming cytochrome c nitrite reductase subunit c552
frdB	4.272	Succinate dehydrogenase/fumarate reductase iron-sulfur subunit
frdA	3.267	Fumarate reductase (quinol) flavoprotein subunit
STM4423	4.903	Putative AraC-type DNA-binding domain-containing protein, ReiD
serB	101.004	Phosphoserine phosphatase SerB
STM0018	-5.240	Chitinase
STM0019	-7.274	Chitinase
uppS	-3.586	(2E,6E)-farnesyl- diphosphate-specific ditrans,polycis-undecaprenyl-diphosphate synthase
fadE	-8.039	Putative acyl-CoA dehydrogenase
STM0360	-13.678	Cytochrome ubiquinol oxidase subunit I
tesA	-6.381	Multifunctional acyl-CoA thioesterase I/protease I/lysophospholipase L1
glxK	-6.033	Glycerate kinase
entD	-10.979	Phosphopantetheinyltransferase
entF	-4.789	Non-ribosomal peptide synthetase
entE	-11.005	2,3-dihydroxybenzoate-AMP ligase
entA	-13.884	2,3-dihydro-2,3-dihydroxybenzoate dehydrogenase
STM0857	-5.232	Putative acyl-CoA dehydrogenase
scsD	-100.766	Protein disulfide oxidoreductase
fhuE	-34.928	Ferric-rhodotorulic acid/ferric-coprogen receptor FhuE
STM1253	-4.129	Cytochrome b
argD	-8.565	Bifunctional succinylornithine transaminase/acetylornithine transaminase
astA	-4.038	Arginine N-succinyltransferase
astD	-5.181	Succinylglutamate-semialdehyde dehydrogenase
sufS	-3.991	Bifunctional cysteine desulfurase/selenocysteine lyase
STM1559	-3.396	Malto-oligosyltrehalose synthase
yncA	-17.005	GNAT family N-acetyltransferase
STM1623	-5.061	Putative carboxylesterase
yciA	-8.628	acyl-CoA esterase
STM1793	-5.679	Cytochrome d ubiquinol oxidase subunit II
udg	-5.658	UDP-glucose 6-dehydrogenase
mrp	-6.910	Fe-S-binding ATPase
yohF	-5.805	SDR family oxidoreductase
fruK	-36.671	1-phosphofructokinase
napB	-16.701	Nitrate reductase

Other functions (continued)		
yhjJ	-3.012	Putative Zn-dependent peptidase
STM4030.S	-3.245	Type II TA system; SehB antitoxin
STM4031	-3.248	Type II TA system; SehA toxin
STM4032.2N	-17.445	Type II TA system; SehC toxin
STM4078	-3.661	Autoinducer 2 aldolase; IsrF
rplL	-61.141	50S ribosomal protein L7/L12
yjfN	-3.763	Putative inner membrane protein
yjgB	-9.718	Putative alcohol dehydrogenase
**Hypothetical, unknown function and pseudogenes**		
STM0271	4.165	Hypothetical protein
hutU	2.846	Pseudogene
STM0801	7.362	Hypothetical protein
STM1666	8.503	Pseudogene
STM1755	4.904	
STM1785	5.542	Hypothetical protein
STM1804.S	4.350	
yfcC	12.258	Hypothetical protein
yfeA	26.871	Hypothetical protein
yhaK	8.908	Hypothetical protein
yhdN	5.468	Hypothetical protein
STM3785	3.335	Hypothetical protein
yiiQ	9.764	hypothetical protein
STM4312	83.809	Putative invasion gene, HilD regulated
STM4313	18.949	Putative invasion gene, HilD regulated
STM4493	4.808	putative cytoplasmic protein
STM4503	3.893	putative inner membrane protein
STM4574	77.706	hypothetical protein
STM0373	-3.259	yaiU; Similar to 3rd module of ATP-binding components of transporters
STM0383	-3.373	yaiB, putative cytoplasmic protein
STM0387	-11.990	yaiI, hypothetical protein
ybaV	-5.087	Hypothetical protein
ybfN	-9.974	Hypothetical protein
STM0759	-10.425	Hypothetical protein
ybhM	-11.362	Hypothetical protein
ybhN	-12.991	Hypothetical protein
ybhP	-8.791	Hypothetical protein
ybiJ	-4.381	Hypothetical protein
STM0860	-5.792	Hypothetical protein
STM0954	-4.912	Hypothetical protein
STM1157	-5.112	Hypothetical protein
pagC	-24.931	Hypothetical protein
STM1254	-3.648	Hypothetical protein
STM1273	-12.380	Hypothetical protein
ynfM	-5.750	Hypothetical protein
STM1586	-3.778	Hypothetical protein
ydcK	-4.234	Hypothetical protein

Metabolism (continued)		
STM2273	-95.577	MR-MLE family protein
pmrF	-4.635	undecaprenyl-phosphate 4-deoxy-4-formamido-L-arabinose transferase
yfbG	-9.193	Bifunctional UDP-glucuronic acid oxidase/UDP-4-amino-4-deoxy-L-arabinose formyltransferase
STM2300	-10.901	4-deoxy-4-formamido-L-arabinose-phosphoundecaprenol deformylase
STM2302	-66.074	4-amino-4-deoxy-L-arabinose-phospho-UDP flippase
STM2341	-4.353	Putative transketolase
ubiX	-4.603	3-octaprenyl-4-hydroxybenzoate carboxy-lyase
sixA	-3.078	Phosphohistidine phosphatase SixA
fadJ	-5.011	Fatty acid oxidation subunit alpha
ddg	-5.367	Lipid A biosynthesis palmitoleoyl acyltransferase
eutA	-77.697	Ethanolamine utilization protein EutA
eutM	-110.573	Ethanolamine utilization protein EutM
talA	-3.584	Transaldolase
STM2537	-18.059	Fe-S assembly protein IscX
fdx	-37.529	ISC system 2Fe-2S type ferredoxin
iroD	-135.681	Enterochelin esterase
nrdE	-12.417	Ribonucleotide-diphosphate reductase subunit alpha
nrdF	-15.531	Class 1b ribonucleoside-diphosphate reductase subunit beta
ygiH	-24.437	Acyl-phosphate--glycerol-3-phosphate O-acyltransferase
oat	-5.276	Putative acetylornithine aminotransferase
garD	-11.449	Galactarate dehydratase
bfd	-6.357	Bacterioferritin-associated ferredoxin
yiaK	-5.111	3-dehydro-L-gulonate 2-dehydrogenase
aldB	-12.112	Aldehyde dehydrogenase
kdtA	-9.702	3-deoxy-D-manno-octulosonic acid transferase
yigP	-5.890	UbiJ protein
fadA	-3.212	3-ketoacyl-CoA thiolase
fadB	-9.686	3-hydroxyacyl-coA dehydrogenase
mobB	-8.349	Molybdopterin-guanine dinucleotide biosynthesis protein B
mobA	-8.097	Molybdenum cofactor guanylyltransferase MobA
dsbA	-4.309	Protein disulfide oxidoreductase DsbA
STM4080	-4.328	Epimerase
argC	-8.744	N-acetyl-gamma-glutamyl-phosphate reductase
yjbA	-8.110	Phosphate-starvation-inducible protein PsiE
phoN	-7.514	Phosphatase PAP2 family protein
aidB	-4.733	Isovaleryl-CoA dehydrogenase
fhuF	-8.134	Siderophore-iron reductase FhuF

Hypothetical, unknown and pseudogenes (continued)		
STM1637	-8.381	Hypothetical protein
STM1866	-4.601	Pseudogene
STM1869	-41.487	Hypothetical protein
STM1941	-16.902	Hypothetical protein
yedD	-4.831	Hypothetical protein
STM2439	-5.381	Hypothetical protein
yfgJ	-19.467	Hypothetical protein
STM2680	-7.512	Hypothetical protein
STM2689	-4.485	Pseudogene
STM2906	-7.240	Putative cytoplasmic protein
STM3026	-3.799	Hypothetical protein
STM3031	-6.300	Hypothetical protein
STM3153	-6.161	Hypothetical protein
yhbU	-31.636	Putative protease
STM3362	-8.050	Hypothetical protein
STM3508	-4.064	Hypothetical protein
yhhL	-5.455	Putative inner membrane protein
STM3657	-4.709	Hypothetical protein
STM3906	-6.102	Hypothetical protein
STM3907	-4.075	Hypothetical protein
STM4011	-12.748	Hypothetical protein
STM05910	-879.939	
STM4111	-24.222	Pseudogene
yjbH	-3.733	Hypothetical protein
STM4575	-4.696	Hypothetical protein
creA	-2.625	Hypothetical protein
**Phage/Prophage proteins**		
STM2597	8.696	Gifsy-1 prophage protein
STM1029	-67.914	Gifsy-2 prophage protein
STM2585	-18.299	SarA/PagJ, *Salmonella*-anti-inflammatory response activator;
STM2709	-85.892	Fels-2 prophage protein
STM4213	-15.075	Phage tail protein

* Significant differences between the Δ*hfq* LSMMG and control cultures were determined according to an FDR < 0.05 and a minimum logFC of 1 or -1 (corresponding to a 2-fold increase or decrease in expression, respectively). LogFC values were converted to fold change. Red shading indicates upregulation in the LSMMG culture, blue shading downregulation in the LSMMG culture.
