# Supplementary material for: Spaceflight Analogue Culture Enhances the Host-Pathogen Interaction Between Salmonella and a 3-D Biomimetic Intestinal Co-Culture Model
Source: Front Cell Infect Microbiol. 2022 May 31;12:705647. doi: 10.3389/fcimb.2022.705647 (PMC9195300; doi:10.3389/fcimb.2022.705647)
Supplement: Supplementary file 10 [file Table_5.pdf]

Supplementary Table 5. Δ*hfq* versus wild type for LSMMG-cultured *S*. Typhimurium *

Gene	Fold Change	Description
**SPI-1 associated genes, effectors and regulators**		
sprB	5.183	Transcriptional regulator
sirC	12.568	Transcriptional regulator SirC (HilC)
orgC	3.114	Invasion protein OrgC
orgB	3.910	Invasion protein OrgB
orgA	5.289	Invasion protein OrgA
prgK	26.490	EscJ/YscJ/HrcJ family type III secretion inner membrane ring protein
prgJ	11.145	type III secretion system protein PrgJ
prgI	3.832	EscF/YscF/HrpA family type III secretion system needle major subunit
prgH	5.117	type III secretion system protein PrgH
hilD	8.009	AraC family transcriptional regulator
hilA	29.845	Transcriptional regulator
iagB	4.797	Invasion protein IagB
sicP	4.250	Chaperone protein SicP
iacP	11.026	Putative acyl carrier protein
sipA	18.606	Pathogenicity island 1 effector protein SipA
sipD	11.968	Cell invasion protein SipD
sipC	7.559	Pathogenicity island 1 effector protein SipC
sipB	5.655	Pathogenicity island 1 effector protein SipB
sicA	3.927	CesD/SycD/LcrH family type III secretion system chaperone
spaO	9.409	type III secretion system protein SpaO
spaN	8.603	Antigen presentation protein SpaN
invI	40.611	Type III secretion system protein SpaM
invC	16.568	EscN/YscN/HrcN family type III secretion system ATPase
invB	14.532	Type III secretion system chaperone SpaK
invA	3.981	EscV/YscV/HrcV family type III secretion system export apparatus protein
invG	4.979	EscC/YscC/HrcC family type III secretion system outer membrane ring protein
sopE2	15.392	Type III secretion protein SopE2
sopA	8.155	Similar to secreted effector protein of *Salmonella* dublin
STM1239	4.776	Effector protein SopF
**SPI-2 effectors**		
sifA	-6.465	Effector protein SifA
STM2139	-34.122	*Salmonella* secreted effector D, SteD

Gene	Fold Change	Description
**Motility and chemotaxis**		
flgA	7.584	Flagella basal body P-ring formation protein FlgA
flgC	4.289	Flagellar basal body rod protein FlgC
flgD	4.426	Flagellar basal body rod modification protein
flgE	3.263	Flagellar hook protein FlgE
flgF	5.151	Flagellar biosynthesis protein FlgF
flgG	4.545	Flagellar basal-body rod protein FlgG
flgH	10.359	Flagellar basal body L-ring protein
fliK	3.939	Flagellar hook-length control protein FliK
fljB	34.223	Flagellin
STM3138	18.107	Chemotaxis protein
STM3216	9.430	Putative methyl-accepting chemotaxis protein
STM3156	5.231	Hypothetical protein, putative class 2 motility gene
ymdF	-16.670	Involved in flagella-dependent motility
yciG	-43.771	Involved in flagella-dependent motility
**Transport**		
bamA	4.068	Outer membrane protein assembly factor BamA
secD	3.878	Protein-export membrane protein SecD
STM0689	7.533	Citrate-proton symporter
artJ	3.252	Arginine ABC transporter substrate-binding protein
artI	4.426	Arginine ABC transporter substrate-binding protein
ompF	4.538	Phosphoporin PhoE
ydjN	5.174	L-cystine transporter
STM1368	3.477	L-cystine transporter
tppB	3.314	Dipeptide and tripeptide permease B
ompD	21.368	Outer membrane protein porin OmpD
oppA	3.983	Oligopeptide transport protein with chaperone properties
nhaB	5.098	Na+/H+ antiporter NhaB
mglA	3.680	Galactose/methyl galactoside ABC transporter ATP-binding protein MglA
glpT	2.782	glycerol-3-phosphate transporter
hisJ	3.144	Histidine ABC transporter substrate-binding protein HisJ
yfiO	3.409	Outer membrane protein assembly factor BamD
srlE	7.528	PTS glucitol/sorbitol transporter subunit IIB

**SPI-4**		
STM4257	238.378	SiiA protein
STM4258	39.387	SiiB protein
STM4259	36.378	ABC transporter, SiiC
STM4260	91.304	Cation transporter, SiiD
STM4261	6.000	SiiE protein
STM4262	13.062	Antibiotic ABC transporter ATP-binding protein
**SPI-5**		
STM1089	8.925	Pathogenicity island encoded protein: SPI5
sigE	5.976	
sopB	10.387	inositol phosphatase
copS	15.480	two-component sensor histidine kinase
pipA	-5.266	virulence protein
**Plasmid**		
PSLT057	37.267	Putative cytoplasmic protein
traS	4.657	Conjugative transfer: surface exclusion
traT	5.005	Conjugative transfer: surface exclusion
PSLT020	-7.403	Hypothetical protein
**Fimbrial proteins/Adhesins**		
stdB	52.263	Fimbrial protein
stdA	36.011	Putative fimbrial-like protein
**Transcriptional and translational regulators**		
STM0354	16.724	Cu(I)-responsive transcriptional regulator
ybdO	4.590	Putative LysR family transcriptional regulator
narP	5.652	DNA-binding response regulator
STM2275	4.023	Putative gntR family regulatory protein
nrdA	3.520	Ribonucleoside-diphosphate reductase subunit alpha
glpQ	3.667	Glycerophosphodiester phosphodiesterase
glpA	3.243	sn-glycerol-3-phosphate dehydrogenase subunit A
glpC	3.123	sn-glycerol-3-phosphate dehydrogenase subunit C
lrhA	3.953	Transcriptional regulator LrhA
argR	7.305	Arginine repressor
STM4315	14.723	AraC family transcriptional regulator
rtsB	11.457	Regulatory protein, LuxR family, RtsB
rtsA	14.723	Regulatory protein, AraC family, RtsA
STM0347	-9.498	Transcriptional regulator
ybeF	-6.094	Putative LysR family transcriptional regulator
STM0835	-12.412	Transcriptional regulator MntR
ynfL	-28.491	Putative LysR family transcriptional regulator
yncC	-10.932	Colanic acid/biofilm transcriptional regulator
ydcI	-4.256	Putative LysR family transcriptional regulator

**Transport (continued)**		
STM3259	33.862	PTS galactitol transporter subunit IIB
yheS	27.426	ABC transporter ATP-binding protein
feoB	5.233	Ferrous iron transporter B
dppB	5.220	Dipeptide ABC transporter permease DppB
dppA	4.665	ABC transporter substrate-binding protein
yidC	6.338	Membrane protein insertase YidC
pstS	4.004	Phosphate ABC transporter substrate-binding protein PstS
trkD	5.264	Potassium transporter Kup
sbp	4.941	Sulfate transporter subunit
STM4195	5.692	Bile acid:sodium symporter
STM4351	3.755	Arginine ABC transporter substrate-binding protein
STM0042	-4.165	MFS transporter
STM0651	-8.712	2-keto-3-deoxygluconate permease
kdpB	-31.367	Potassium-transporting ATPase subunit B
yceL	-8.653	MFS transporter
chaB	-7.156	Cation transport regulator
pduF	-9.819	Aquaporin; propanediol diffusion facilitator
yehW	-3.972	ABC transporter permease
yehY	-3.630	ABC transporter permease
yehZ	-4.206	ABC transporter substrate-binding protein
yejE	-4.114	Microcin ABC transporter permease
STM2344	-4.094	PTS ascorbate transporter subunit IIA
cysZ	-3.252	Sulfate transporter CysZ
STM2551	-15.586	Nickel transporter
STM2690	-4.004	Type I secretion protein TolC, BapB
nxiA	-3.807	Putative nickel transporter
yggB	-5.171	Mechanosensitive ion channel protein MscS
STM3134	-4.549	MFS transporter
exbD	-26.434	TonB system transporter ExbD
STM3256	-10.056	PTS fructose transporter subunit IIA
nanT	-4.324	MFS transporter
dgoT	-9.562	MFS transporter
STM4065	-15.453	MFS transporter
STM4074	-3.787	Autoinducer 2 ABC transporter ATP-binding protein LsrA
STM4075	-4.337	Autoinducer 2 ABC transporter permease LsrC
malE	-3.350	Maltose transport protein
malK	-33.507	ABC transporter ATP-binding protein
**Other functions**		
STM0062	66.801	holo-ACP synthase CitX
STM0159	3.291	putative restriction endonuclease
htrA	3.653	serine endoprotease

Transcriptional and translational regulators (continued)		
STM3012	-4.346	Putative transcriptional regulator
STM3124	-3.873	Helix-turn-helix transcriptional regulator
yiaG	-7.849	Putative transcriptional regulator
xylR	-4.559	XylR family transcriptional regulator
dgoR	-6.917	Galactonate operon transcriptional repressor
**Metabolism**		
dapB	4.547	4-hydroxy-tetrahydrodipicolinate reductase
carA	3.817	Carbamoyl phosphate synthase small subunit
carB	5.974	Carbamoyl phosphate synthase large subunit
pepD	7.333	Cytosol nonspecific dipeptidase
prpC	3.386	2-methylcitrate synthase
prpD	3.141	2-methylcitrate dehydratase
allD	5.775	Ureidoglycolate dehydrogenase
STM0572	23.189	phosphosugar isomerase
ybdL	4.469	methionine aminotransferase
ybdN	4.953	phosphoadenosine phosphosulfate reductase
lnt	5.329	apolipoprotein N-acyltransferase
ybfM	95.673	chitoporin
ybfN	101.019	Chitoporin
sdhC	4.032	succinate dehydrogenase, cytochrome b556
sdhD	3.963	succinate dehydrogenase, hydrophobic membrane anchor protein
sucB	3.085	dihydrolipoamide succinyltransferase
galK	5.237	galactokinase
pyrD	4.226	dihydroorotate dehydrogenase (quinone)
agp	6.558	bifunctional glucose-1-phosphatase/inositol phosphatase
pyrC	3.126	dihydroorotase
fabG	3.471	3-oxoacyl-ACP reductase
purB	3.080	adenylosuccinate lyase
ttrA	3.175	tetrathionate reductase subunit A
ttrC	14.156	tetrathionate reductase subunit C
ttrB	10.603	tetrathionate reductase complex, subunit B
STM1498	7.645	dimethyl sulfoxide reductase subunit A
STM1532	20.512	ATP/GTP-binding protein
STM1538	6.358	putative hydrogenase-1 large subunit
STM1539	2.961	putative hydrogenase-1 small subunit
prsA	4.742	ribose-phosphate pyrophosphokinase
pgsA	5.092	CDP-diacylglycerol--glycerol-3-phosphate 3-phosphatidyltransferase
cbiB	19.970	Cobalamin biosynthesis protein CbiB
yfbQ	7.348	aminotransferase
upp	3.430	uracil phosphoribosyltransferase
ndk	3.968	nucleoside-diphosphate kinase
STM2530	3.667	putative anaerobic dimethylsulfoxide reductase
STM2754	5.595	hexulose-6-phosphate synthase
sdaB	5.414	L-serine ammonia-lyase
argA	5.597	amino-acid N-acetyltransferase
serA	3.736	D-3-phosphoglycerate dehydrogenase
hybB	5.159	Ni/Fe-hydrogenase cytochrome b subunit
hybA	9.770	hydrogenase 2 protein HybA

Other functions (continued)		
STM0225	4.385	Outer membrane protein, OmpH
mod	4.744	restriction endonuclease
STM0474	4.844	Hha toxicity attenuator
STM0724	19.363	glycosyl transferase
STM1253	4.635	cytochrome b
rfc	4.154	polymerase
ftnB	4.755	Ferritin
pheS	3.787	phenylalanine--tRNA ligase subunit alpha
rtn	3.597	phage resistance protein
STM2238	7.486	NTPase
rimM	4.646	ribosome maturation factor RimM
recN	5.420	DNA repair protein RecN
STM2767	4.122	DNA helicase
yqgB	38.157	virulence-promoting factor
yraO	14.458	putative phosphoheptose isomerase
yraP	8.872	osmotically-inducible protein OsmY
rpmA	6.932	50S ribosomal protein L27
sun	6.993	16S rRNA (cytosine(967)-C(5))-methyltransferase
rpsN	4.801	30S ribosomal protein S14
rplE	5.111	50S ribosomal protein L5
rplB	6.310	50S ribosomal protein L2
rplW	4.057	50S ribosomal protein L23
rplC	5.328	50S ribosomal protein L3
rpsJ	3.249	30S ribosomal protein S10
fusA	3.757	translation elongation factor G
rpsG	4.529	30S ribosomal protein S7
fkpA	3.516	FKBP-type peptidyl-prolyl cis-trans isomerase FkpA
yiaD	7.768	OmpA family lipoprotein
lldP	9.827	L-lactate permease
spoU	6.414	tRNA (guanosine(18)-2'-O)-methyltransferase TrmH
tufB	10.122	Translation elongation factor Tu
rplA	7.796	50S ribosomal protein L1
rplJ	4.319	50S ribosomal protein L10
rpsF	5.105	30S ribosomal protein S6
priB	3.639	primosomal replication protein N
rpsR	7.049	30S ribosomal protein S18
rplI	8.808	50S ribosomal protein L9
STM4496	6.616	DNA repair protein
hsdS	4.539	restriction endonuclease subunit S
STM0291	-5.687	putative RHS-family protein
STM0359	-21.637	Salmonella secreted substrate A, SssA
xseB	-4.395	exodeoxyribonuclease VII small subunit
dps	-3.011	DNA starvation/stationary phase protection protein Dps
STM0948	-6.300	transposase
STM1158	-4.365	cytochrome B
ydeI	-11.317	TIGR00156 family protein
osmC	-4.291	OsmC family peroxiredoxin
yncD	-3.446	TonB-dependent receptor
STM1678	-5.011	aromatic alcohol reductase

Metabolism (continued)		
hypO	4.438	hydrogenase 2 small subunit
yqhD	4.165	NADH-dependent alcohol dehydrogenase
argG	5.197	argininosuccinate synthetase
pckA	3.060	phosphoenolpyruvate carboxykinase (ATP)
STM3820	10.786	cytochrome-c peroxidase
dnaN	5.626	DNA polymerase III subunit beta
atpG	4.787	F0F1 ATP synthase subunit gamma
ilvC	4.689	ketol-acid reductoisomerase
plsB	3.051	glycerol-3-phosphate 1-O-acyltransferase
nrfA	6.055	ammonia-forming cytochrome c nitrite reductase subunit c552
frdC	5.531	fumarate reductase subunit C
frdB	3.799	succinate dehydrogenase/fumarate reductase iron-sulfur subunit
pyrB	4.879	aspartate carbamoyltransferase catalytic subunit
fadE	-3.381	putative acyl-CoA dehydrogenase
STM0332	-5.043	hydrolase
STM0360	-33.785	cytochrome ubiquinol oxidase subunit I
hutG	-3.520	formimidoylglutamase
ybhO	-4.549	cardiolipin synthase B
mdaA	-4.692	nitroreductase A
argD	-10.766	bifunctional succinylornithine transaminase/acetylornithine transaminase
astA	-9.817	arginine N-succinyltransferase
astD	-6.412	succinylglutamate-semialdehyde dehydrogenase
katE	-2.990	catalase HPII
sufA	-4.720	Fe-S cluster assembly scaffold SufA
sufB	-6.160	Fe-S cluster assembly protein SufB
sufC	-4.851	Fe-S cluster assembly ATPase SufC
sufS	-3.651	bifunctional cysteine desulfurase/selenocysteine lyase
STM1559	-6.972	malto-oligosyltrehalose synthase
yncB	-4.077	putative NADP-dependent oxidoreductase
fbaB	-4.843	class I fructose-bisphosphate aldolase
yohF	-10.694	SDR family oxidoreductase
STM2175	-7.693	salicylate hydroxylase
nuoA	-2.666	NADH-quinone oxidoreductase subunit A
STM2341	-4.680	putative transketolase
talA	-3.818	transaldolase
tktB	-3.096	transketolase
nrdE	-5.201	ribonucleotide-diphosphate reductase subunit alpha
STM2959	-3.785	glycerate kinase
STM3135	-3.442	mannonate dehydratase
STM3136	-8.347	fructuronate reductase
STM3137	-3.563	glucuronate isomerase
yghA	-8.904	NAD(P)-dependent oxidoreductase
nanK	-3.809	N-acetylmannosamine kinase
nanA	-7.579	N-acetylneuraminate lyase
bfr	-6.354	bacterioferritin
malQ	-3.592	4-alpha-glucanotransferase
STM3697	-3.845	mandelate racemase/muconate lactonizing protein
dgoA	-15.740	2-oxo-3-deoxygalactonate 6-phosphate aldolase

Other functions (continued)		
STM1731	-44.994	Mn-containing catalase
rrmA	-16.691	23S rRNA (guanine(745)-N(1))-methyltransferase
yecD	-5.018	hydrolase
cspB	-50.731	putative cold-shock protein
elaB	-5.286	Inner membrane protein ElaB
glrK	-5.107	two-component system, NtrC family, sensor histidine kinase GlrK
STM2689	-14.645	Biofilm-associated protein, Bap
hin	-8.605	DNA-invertase
ygaF	-3.925	hydroxyglutarate oxidase
ygaU	-9.655	peptidoglycan-binding protein LysM
ygaM	-4.952	putative inner membrane protein
STM3132	-5.646	xylanase deacetylase
ygiW	-4.044	TIGR00156 family protein
rnpB	-17.359	ncRNA
rrfF	-31.692	5S ribosomal RNA
hopD	-28.668	prepilin peptidase
fic	-6.768	cell filamentation protein Fic
STM3521	-6.302	RNA-binding protein
STM4078	-3.203	autoinducer 2 aldolase
ecnB	-8.893	putative entericidin B precursor
amiB	-4.808	N-acetylmuramoyl-L-alanine amidase AmiB
yjgB	-9.450	putative alcohol dehydrogenase
rimI	-6.574	ribosomal-protein-alanine N-acetyltransferase
hfq	-153.522	RNA chaperone Hfq
**Hypothetical, unknown function and pseudogenes**		
STM0726	50.772	putative glycosyl transferase
STM0763.s	5.872	
STM1131	11.714	hypothetical protein
STM1250	6.454	putative cytoplasmic protein
STM1254	70.758	hypothetical protein
STM1672	3.253	hypothetical protein
STM1785	9.836	hypothetical protein
STM2007	7.719	putative TPR repeat protein
yfaZ	7.129	putative inner membrane protein
STM2746	7.185	putative excinuclease ATPase subunit
STM2747	22.286	putative cytoplasmic protein
STM2766	57.680	hypothetical protein
STM3547.Sc	7.844	
STM3720	9.274	putative inner membrane protein
STM3845	3.574	hypothetical protein
STM3846.s	4.084	
STM4196	4.496	hypothetical protein
STM4305.S	2.713	
STM4313	32.219	putative cytoplasmic protein
STM4472	8.089	hypothetical protein
STM4574	14.911	hypothetical protein
STM0014	-14.338	hypothetical protein
STM0328.s	-29.651	

**Metabolism (continued)**		
dgoK	-13.910	2-oxo-3-deoxygalactonate kinase
rbsD	-7.223	D-ribose pyranase
fadA	-4.570	3-ketoacyl-CoA thiolase
STM4066	-13.889	aminoimidazole riboside kinase
STM4067	-20.062	putative ADP-ribosylglycohydrolase
aceK	-5.824	bifunctional isocitrate dehydrogenase kinase/phosphatase
**LPS Biosynthesis**		
STM0557	3.957	putative inner membrane protein
STM1328	9.428	Lipid A 3′-O-deacylase, LpxR
rfbP	5.679	UDP-phosphate galactose phosphotransferase
rfbN	3.676	rhamnosyltransferase
rfbU	4.549	mannosyl transferase
rfbX	5.380	putative O-antigen transferase
rfbJ	8.016	CDP-abequose synthase
yhjW	3.326	lipid A phosphoethanolamine transferase
rfaK	4.136	UDP-glucose--(glucosyl)LPS alpha-1,2-glucosyltransferase
rfaY	9.493	heptose kinase
rfaJ	7.432	lipopolysaccharide 1,2-glucosyltransferase
rfaI	2.705	lipopolysaccharide 1,3-galactosyltransferase
STM4118	3.952	Phosphoethanolamine transferase CptA
argB	3.472	acetylglutamate kinase
coaA	22.777	type I pantothenate kinase
**Phage/Prophage proteins**		
STM1054	5.354	Gifsy-2 prophage protein
STM4212	35.737	phage major tail tube protein

**Hypothetical, unknown and pseudogenes (continued)**		
STM0346	-17.420	hypothetical protein
ybaY	-6.269	hypothetical protein
STM0566	-38.639	hypothetical protein
STM0759	-13.188	hypothetical protein
STM0810	-12.644	hypothetical protein
ybhN	-22.643	hypothetical protein
ybhP	-10.987	hypothetical protein
ycaP	-6.424	hypothetical protein
STM1077	-3.928	hypothetical protein
ycdF	-11.952	pseudogene
STM1133	-2.770	putative dehydrogenases and related proteins
yceK	-3.880	hypothetical protein
STM1389	-3.317	hypothetical protein
STM1546	-4.557	hypothetical protein
STM1561	-5.871	hypothetical protein
STM1624	-3.664	DUF1338 domain-containing protein
yciF	-56.533	hypothetical protein
yciE	-86.650	hypothetical protein
STM1851	-8.090	hypothetical protein
yfdC	-3.853	hypothetical protein
STM2484	-4.307	hypothetical protein
yfgJ	-9.527	hypothetical protein
STM05520	-6.709	
yfiH	-3.052	hypothetical protein
STM2789	-8.418	putative cytoplasmic protein
ygdI	-6.271	hypothetical protein
STM3021	-4.916	putative inner membrane protein
yqjC	-4.676	hypothetical protein
yheV	-6.096	hypothetical protein
yhjG	3.724	hypothetical protein
STM3828.1N	-7.042	
STM4111	-50.939	pseudo
yjbJ	-5.723	putative cytoplasmic protein
STM4562	-6.202	putative inner membrane protein

* Significant differences between wild type and Δ*hfq* cultures grown in the LSMMG condition only were determined according to an FDR < 0.05 and a minimum logFC of 1 or -1 (corresponding to a 2-fold increase or decrease in expression, respectively). LogFC values were converted to fold change. Red shading indicates upregulation in the mutant and blue shading downregulation.
