# Supplementary material for: Spaceflight Analogue Culture Enhances the Host-Pathogen Interaction Between Salmonella and a 3-D Biomimetic Intestinal Co-Culture Model
Source: Front Cell Infect Microbiol. 2022 May 31;12:705647. doi: 10.3389/fcimb.2022.705647 (PMC9195300; doi:10.3389/fcimb.2022.705647)
Supplement: Supplementary file 11 [file Table_6.pdf]

Supplementary Table 6. Δ*hfq* versus wild type for control-cultured *S.* Typhimurium*

Gene	Fold Change	Description
**SPI-1**		
invF	9.684	Invasion protein
**SPI-2 associated genes and effectors**		
ssrB	4.452	DNA-binding response regulator
ssrA	5.425	Hybrid sensor histidine kinase/response regulator
sseB	3.417	Enhances serine sensitivity
ssaK	19.594	Type III secretion system protein SsaK
ssaO	6.522	Type III secretion system protein SsaO
sifA	-5.399	Effector protein SifA
ssaE	-13.839	EscE/YscE/SsaE family type III secretion system needle protein co-chaperone
ssaL	-4.942	Secretion system apparatus protein
ssaM	-14.390	Type III secretion system protein SsaM
ssaV	-6.476	EscV/YscV/HrcV family type III secretion system export apparatus
STM1698	-3.123	Secreted effector kinase SteC
STM2139	-61.217	*Salmonella* secreted effector D, SteD
**SPI-4**		
STM4257	12.308	SiiA protein
STM4259	5.586	ABC transporter, SiiC
STM4260	6.882	Cation transporter, SiiD
**Plasmid**		
PSLT061	6.379	Putative inner membrane protein
PSLT068	3.770	Putative ParB-like nuclease domain
traN	9.075	Conjugative transfer: aggregate stability
traQ	15.641	Conjugative transfer: fimbrial synthesis
trbB	7.199	Conjugative transfer
traT	3.807	Conjugative transfer: surface exclusion
PSLT106	3.800	Homologue of mvpA, Shigella flexneri
traI	8.965	Conjugative transfer: oriT nicking-unwinding
**Metabolism**		
acnB	4.905	Bifunctional aconitate hydratase 2/2-methylisocitrate dehydratase
speE	3.085	Putrescine aminopropyltransferase
cueO	4.204	Putative multicopper oxidase
yaeR	3.690	VOC family protein
fadE	5.864	Putative acyl-CoA dehydrogenase
hemB	4.623	delta-aminolevulinic acid dehydratase
proC	5.639	Pyrroline-5-carboxylate reductase
cyoA	4.569	Cytochrome o ubiquinol oxidase subunit II
entA	4.822	2,3-dihydro-2,3-dihydroxybenzoate dehydrogenase
cstA	3.385	Carbon starvation protein A
ybfM	457.018	Chitoporin
ybfN	601.301	Chitoporin
sdhD	5.978	Succinate dehydrogenase, hydrophobic membrane anchor protein
sdhB	3.137	Succinate dehydrogenase, Fe-S protein

Gene	Fold Change	Description
**Motility and chemotaxis**		
fliH	62.138	Flagellar assembly protein FliH
STM3138	3.964	Chemotaxis protein
fliA	-5.142	RNA polymerase sigma factor FliA
fliB	-8.226	Lysine-N-methylase
fliQ	-62.398	Flagellar export apparatus protein FliQ
cheY	-4.036	Two-component system response regulator
cheR	-5.795	Chemotaxis protein-glutamate O-methyltransferase
cheW	-75.976	Chemotaxis protein CheW
STM3152	-17.348	Putative methyl-accepting chemotaxis protein
ymdF	-38.347	Involved in flagella-dependent motility
yciG	-76.593	Involved in flagella-dependent motility
**Fimbrial proteins/Adhesins**		
stdA	18.031	Putative fimbrial-like protein
bcfA	-65.705	Fimbrial protein
safB	-13.034	Putative fimbriae assembly chaparone
stbD	-6.478	Fimbrial protein
lpfA	-7.663	Long polar fimbria
stcD	-8.495	Adhesin
stcB	-49.023	Putative fimbrial chaperone protein
stjB	-4.380	Fimbrial assembly protein
stjC	-9.457	Fimbrial chaperone protein
STM4593	-16.911	Putative fimbrial usher protein
STM4594	-35.233	Fimbrial assembly chaperone SthB
shdA	-3.649	C-terminal region of AIDA-like protein; autotransported adhesin ShdA
**Transcriptional and translational regulators**		
ybdM	6.784	Transcriptional regulator related to Sp0J
marA	4.807	AraC/XylS family transcriptional activator of defense systems
pspC	6.277	DNA-binding transcriptional activator PspC
cysB	5.060	Transcriptional regulator CysB
agaR	26.181	DeoR family transcriptional regulator
crp	4.165	Transcriptional regulator Crp
STM3794	4.600	DeoR family transcriptional regulator
STM4315	8.164	AraC family transcriptional regulator
yaiV	-8.340	Transcriptional regulator
ybeF	-5.909	Putative LysR family transcriptional regulator
yncC	-83.487	Colanic acid/biofilm transcriptional regulator
yncB	-4.255	Putative NADP-dependent oxidoreductase
STM1620	-113.350	Lactate oxidase
STM1671	-7.331	Putative bacterial regulatory helix-turn-helix protein, araC family
ygaE	-12.483	Transcriptional regulator
STM2797	-4.427	Transcriptional regulator
stpA	-5.731	DNA-binding protein

Metabolism (continued)		
sucA	3.992	2-oxoglutarate dehydrogenase E1 component
sucB	3.145	Dihydrolipoamide succinyltransferase
sucC	3.946	Succinate--CoA ligase subunit beta
cydA	14.991	Cytochrome d terminal oxidase subunit 1
nadA	10.252	Quinolinate synthase
hpaC	6.465	4-hydroxyphenylacetate 3-monooxygenase, reductase component
scsB	13.085	Protein-disulfide reductase
scsD	45.777	Protein disulfide oxidoreductase
agp	25.020	Bifunctional glucose-1-phosphatase/inositol phosphatase
fhuE	9.314	Ferric-rhodotorulic acid/ferric-coprogen receptor FhuE
icdA	2.759	NADP-dependent isocitrate dehydrogenase
pdxY	5.167	Pyridoxal kinase
fumA	3.757	Fumarate hydratase
fumC	3.279	Class II fumarate hydratase
STM1538	5.737	Putative hydrogenase-1 large subunit
yncA	20.125	GNAT family N-acetyltransferase
STM1612	7.287	Aminopeptidase
fabI	4.505	Enoyl-[acyl-carrier-protein] reductase
pduG	10.126	Diol dehydratase reactivase subunit alpha
phsA	2.578	Thiosulfate reductase PhsA
cpsG	4.858	Phosphomannomutase
mrp	10.934	Fe-S-binding ATPase
STM2178	5.591	Gentisate 1,2-dioxygenase
napB	18.441	Nitrate reductase
glpQ	16.137	Glycerophosphodiester phosphodiesterase
glpA	3.439	sn-glycerol-3-phosphate dehydrogenase subunit A
glpC	4.675	sn-glycerol-3-phosphate dehydrogenase subunit C
yfbG	5.265	Bifunctional UDP-glucuronic acid oxidase/UDP-4-amino-4-deoxy-L-arabinose formyltransferase
nuoF	3.249	NADH-quinone oxidoreductase subunit F
yfbQ	7.697	Aminotransferase
sixA	3.384	Phosphohistidine phosphatase SixA
yfgD	3.002	Putative arsenate reductase
upp	4.918	Uracil phosphoribosyltransferase
STM2537	36.102	Fe-S assembly protein IscX
fdx	44.137	ISC system 2Fe-2S type ferredoxin
hscA	3.195	Fe-S protein assembly chaperone HscA
pdxJ	4.787	Pyridoxine 5'-phosphate synthase
STM2754	4.726	Hexulose-6-phosphate synthase
eno	5.497	Phosphopyruvate hydratase
mazG	3.659	Nucleoside triphosphate pyrophosphohydrolase
gudD	5.339	Glucarate dehydratase

Transcriptional and translational regulators (continued)		
fucR	-4.828	Transcriptional regulator
yohL	-4.775	transcriptional regulator
STM3358	-4.458	GntR family transcriptional regulator
yiaG	-14.954	putative transcriptional regulator
xylR	-5.693	XylR family transcriptional regulator
mtlR	-5.193	mannitol operon repressor
rhaR	-5.546	AraC family transcriptional regulator
STM4270	-5.774	putative LysR family transcriptional regulator
**Transport**		
caiT	8.203	L-carnitine:gamma-butyrobetaine antiporter
tbpA	6.637	Thiamine ABC transporter substrate binding subunit
fhuA	6.324	Ferrichrome porin FhuA
bamA	4.574	Outer membrane protein assembly factor BamA
STM0355	11.040	Heavy metal transport/detoxification protein
fepA	5.043	Outer membrane receptor protein
fepC	16.113	Iron-enterobactin transporter ATP-binding protein
nagE	3.174	PTS N-acetyl glucosamine transporter subunit IIABC
gltI	3.122	Glutamate/aspartate transporter
STM0689	3.766	Citrate-proton symporter
glnQ	3.752	Glutamine ABC transporter ATP-binding protein
glnP	7.678	Glutamine ABC transporter permease GlnP
glnH	18.926	Glutamine ABC transporter substrate-binding protein GlnH
artQ	6.240	Arginine transporter permease subunit ArtQ
ompF	22.173	Phosphoporin PhoE
putP	4.209	Sodium/proline symporter
STM1259	13.479	Peptide ABC transporter ATP-binding protein
STM1491	3.260	Glycine/betaine ABC transporter ATP-binding protein
STM1492	9.590	Choline ABC transporter permease
STM1493	6.871	Glycine/betaine ABC transporter substrate-binding protein
ompD	30.798	Outer membrane protein porin OmpD
STM1614	26.486	PTS sugar transporter subunit IIC
STM1633	13.974	D-alanine transporter, DalS
oppF	2.877	Oligopeptide ABC transporter ATP-binding protein OppF
oppA	10.092	Oligopeptide transport protein with chaperone properties
nhaB	3.676	Na+/H+ antiporter NhaB
yegT	3.575	MFS transporter
mglC	3.909	Galactoside ABC transporter permease MglC
mglA	6.322	Galactose/methyl galactoside ABC transporter ATP-binding protein MglA
cirA	3.635	Catecholate siderophore receptor CirA
ompC	26.164	Porin OmpC
glpT	9.026	Glycerol-3-phosphate transporter

Metabolism (continued)		
gcvT	18.046	Glycine cleavage system protein T
tktA	2.847	Transketolase
ansB	15.992	L-asparaginase II
hybD	4.610	Hydrogenase expression/formation protein
ygiH	9.232	Acyl-phosphate--glycerol-3-phosphate O-acyltransferase
garD	9.578	Galactarate dehydratase
gltB	6.254	Glutamate synthase large subunit
mdh	2.957	Malate dehydrogenase
asd	2.728	Aspartate-semialdehyde dehydrogenase
yiaK	6.931	3-dehydro-L-gulonate 2-dehydrogenase
lyxK	15.860	Carbohydrate kinase
kdtA	10.434	3-deoxy-D-manno-octulosonic acid transferase
ilvB	3.507	Acetolactate synthase I large subunit
STM3820	5.381	Cytochrome-c peroxidase
atpG	4.551	F0F1 ATP synthase subunit gamma
asnA	3.975	aspartate--ammonia ligase
yigP	5.058	UbiJ protein
fadB	5.828	3-hydroxyacyl-coA dehydrogenase
mobB	9.211	Molybdopterin-guanine dinucleotide biosynthesis protein B
mobA	7.153	Molybdenum cofactor guanylyltransferase MobA
dsbA	7.376	Protein disulfide oxidoreductase DsbA
ushB	3.674	CDP-diacylglycerol diphosphatase
fpr	5.970	Ferredoxin--NADP(+) reductase
glpK	9.258	Glycerol kinase
ppc	5.210	Phosphoenolpyruvate carboxylase
argC	14.780	N-acetyl-gamma-glutamyl-phosphate reductase
aceB	4.082	Malate synthase A
aceA	4.830	Isocitrate lyase
aphA	3.138	Acid phosphatase/phosphotransferase
cpdB	5.310	2',3'-cyclic-nucleotide 2'-phosphodiesterase
fbp	3.279	Fructose-bisphosphatase
cybC	5.652	Cytochrome b562
hemL	-16.792	glutamate-1-semialdehyde-2,1-aminomutase
STM0330	-71.574	3-isopropylmalate dehydratase small subunit
STM0360	-6.261	cytochrome ubiquinol oxidase subunit I
STM0361	-8.449	cytochrome BD2 subunit II
phoR	-5.321	two-component system sensor histidine kinase PhoR
ybdR	-8.907	glutathione-dependent formaldehyde dehydrogenase
STM0855	-4.189	putative electron transfer flavoprotein beta subunit
STM0856	-8.410	electron transfer flavoprotein subunit alpha
lpxK	-68.495	tetraacyldisaccharide 4'-kinase
STM1002	-12.977	Diaminopropionate ammonia-lyase
STM1119	-5.310	NAD(P)H:quinone oxidoreductase, type IV
STM1267	-2.612	histidine kinase

Transport (continued)		
nupC	9.360	Nucleoside permease
yfiO	4.169	Outer membrane protein assembly factor BamD
emrA	7.017	Multidrug export protein EmrA
ygjU	64.514	Serine/threonine transporter SstT
STM3075	11.167	ABC transporter ATP-binding protein
STM3169	3.437	C4-dicarboxylate ABC transporter substrate-binding protein
tolC	3.729	Outer membrane channel
feoB	9.089	Ferrous iron transporter B
yhgG	70.870	Ferrous iron transporter C
livK	5.969	Leucine ABC transporter subunit substrate-binding protein LivK
dppD	3.139	Dipeptide ABC transporter ATP-binding protein
dppB	3.966	Dipeptide ABC transporter permease DppB
dppA	6.708	ABC transporter substrate-binding protein
emrD	3.122	Multidrug transporter EmrD
dgoT	5.349	MFS transporter
dcuA	17.218	C4-dicarboxylate transporter
glpF	8.505	Aquaporin
STM4351	3.584	Arginine ABC transporter substrate-binding protein
yjbB	3.471	PNaS family transporter
lamB	7.075	Maltoporin
yadQ	-4.758	chloride channel protein, CIC family
STM0356	-4.600	MFS transporter
STM0520	-9.537	MFS transporter
allP	-81.265	putative NCS1 family, allantoin transport protein
STM0718	-147.816	transport protein
STM0722	-9.695	ABC transporter permease
STM0765	-17.928	putative cation transporter
modB	-52.735	molybdate ABC transporter permease
ycaD	-5.028	MFS transporter
ycaM	-9.340	transporter
STM1128	-2.947	acetylneuraminate ABC transporter
STM1132	-13.710	MFS transporter
STM1256	-6.445	peptide ABC transporter permease
ydiM	-9.133	MFS transporter
STM1634	-61.681	amino acid ABC transporter ATP-binding protein
STM1636	-6.464	amino acid ABC transporter permease
STM1668	-7.108	ZirS protein, secreted by zirT
narK	-5.015	nitrate/nitrite transporter
chaB	-3.583	cation transport regulator
znuB	-6.184	zinc ABC transporter permease
yehY	-3.599	ABC transporter permease
yehZ	-4.151	ABC transporter substrate-binding protein
lysP	-6.347	lysine transporter
setB	-10.349	sugar efflux transporter SetB

Metabolism (continued)		
celF	-9.371	6-phospho-beta-glucosidase
ydiR	-39.449	electron transfer flavoprotein subunit alpha
sufA	-3.358	Fe-S cluster assembly scaffold SufA
rsxB	-3.588	electron transport complex subunit RsxB
STM1559	-3.580	malto-oligosyltrehalose synthase
STM1560	-6.802	malto-oligosyltrehalose trehalohydrolase
pabB	-4.833	p-aminobenzoate synthetase, component I
STM1836	-3.435	peptidoglycan synthase, PBP3SAL
pphA	-3.877	serine/threonine-protein phosphatase, PrpA
STM1939	-4.542	putative glucose-6-phosphate dehydrogenase
yedP	-16.768	mannosyl-3-phosphoglycerate phosphatase
erfK	-5.030	L,D-transpeptidase
hisI	-3.911	bifunctional phosphoribosyl-AMP cyclohydrolase/phosphoribosyl-ATP diphosphatase
wcaM	-13.877	colanic acid biosynthesis protein WcaM
wcaD	-67.650	colanic acid polymerase WcaD
fbaB	-3.692	class I fructose-bisphosphate aldolase
yohF	-3.334	SDR family oxidoreductase
yfbB	-10.194	2-succinyl-6-hydroxy-2,4-cyclohexadiene-1-carboxylate synthase
nuoA	-5.211	NADH-quinone oxidoreductase subunit A
eutT	-38.357	cobalamin adenosyltransferase
murQ	-8.815	N-acetylmuramic acid 6-phosphate etherase
luxS	-4.520	S-ribosylhomocysteine lyase
mltB	-4.461	murein transglycosylase B
kduI	-46.265	pectin-degrading enzyme
hybG	-12.986	hydrogenase 2 accessory protein HypG
yghA	-8.763	NAD(P)-dependent oxidoreductase
yqjG	-3.500	glutathione-dependent reductase
yraR	-5.112	putative nucleoside-diphosphate-sugar epimerase
gltD	-10.171	glutamate synthase small subunit
STM3598	-5.696	putative L-asparaginase
STM3927	-39.165	4-alpha-L-fucosyltransferase
yigW	-11.778	hydrolase TatD
pflC	-4.544	[formate-C-acetyltransferase]-activating enzyme
STM4205	-5.552	glycosyltransferase
yjeF	-3.768	bifunctional ADP-dependent (S)-NAD(P)H-hydrate dehydratase/NAD(P)H-hydrate epimerase
STM4433	-86.485	inositol 2-dehydrogenase
serB	-121.623	phosphoserine phosphatase SerB
**Other functions**		
surA	2.899	Peptidylprolyl isomerase SurA
STM0159	3.234	Putative restriction endonuclease
htrA	9.649	Serine endoprotease
STM0225	4.499	Outer membrane protein, OmpH

Transport (continued)		
yfaV	-7.879	MFS transporter
STM2359	-5.910	putative amino acid transporter
cysU	-4.627	sulfate ABC transporter permease subunit CysT
STM2690	-4.721	type I secretion protein TolC
nxiA	-3.767	Putative nickel transporter
araE	-6.643	arabinose:proton symporter
yggB	-5.569	Mechanosensitive ion channel protein MscS
STM3134	-3.628	MFS transporter
STM3771	-40.633	PTS sugar transporter subunit IIB
yieO	-5.031	MFS transporter
STM4206	-6.333	translocase
yjeM	-5.815	Glutamate/gamma-aminobutyrate family transporter YjeM
ulaA	-60.555	PTS system ascorbate-specific transporter subunit IIC
STM4418	-6.499	MFS transporter
yjiJ	-3.734	MFS transporter
**LPS biosynthesis**		
imp	3.475	LPS assembly protein LptD
lpxA	5.334	acyl-[acyl-carrier-protein]--UDP-N-acetylglucosamine O-acyltransferase
STM0557	3.680	putative inner membrane protein
fepE	6.497	LPS O-antigen length regulator
rfbP	3.687	UDP-phosphate galactose phosphotransferase
rfbJ	8.150	CDP-abequose synthase
ddg	5.950	Lipid A biosynthesis palmitoleoyl acyltransferase
yhjW	8.308	Lipid A phosphoethanolamine transferase
rfaJ	4.853	lipopolysaccharide 1,2-glucosyltransferase
STM4118	3.238	Phosphoethanolamine transferase CptA
wzzB	-3.771	LPS O-antigen chain length determinant protein WzzB
STM2208	-318.263	O-antigen phase variation protein, Opf B
**Hypothetical, unknown function and pseudogenes**		
ybaV	10.782	Hypothetical protein
ybbN	3.450	Co-chaperone YbbN
STM1169	5.551	Putative virulence factor MviM
STM1254	260.290	Hypothetical protein
STM1540	4.327	Putative hydrolase
slp	4.932	Hypothetical protein
mtfA	10.056	Hypothetical protein
yfaZ	10.936	Putative inner membrane protein
STM2439	3.859	Hypothetical protein
yfeZ	5.717	Hypothetical protein
STM2494	3.697	Hypothetical protein
yfgM	4.144	Hypothetical protein
STM2532	4.386	Hypothetical protein

Other functions (continued)		
yafK	7.510	Transpeptidase
mod	4.059	Restriction endonuclease
rna	4.744	Ribonuclease I
pal	4.876	Peptidoglycan-associated lipoprotein
ybgF	6.097	Cell division protein CpoB
STM1085	3.754	BAX inhibitor protein
yccD	4.652	Chaperone-modulator protein CbpM
rne	2.920	Ribonuclease E
lpoB	4.207	Penicillin-binding protein activator LpoB
ycfS	7.645	L,D-transpeptidase
STM1253	23.554	Cytochrome b
rfc	4.696	Polymerase
pdgL	4.340	D-alanyl-D-alanine dipeptidase
pspB	3.476	Phage shock protein B
pspA	3.629	Phage shock protein PspA
umuC	21.708	DNA polymerase V subunit UmuC
STM2238	8.506	NTPase
elaC	8.790	Ribonuclease Z
amiA	2.801	N-acetylmuramoyl-l-alanine amidase I
yfgB	9.137	Bifunctional tRNA (adenosine(37)-C2)-methyltransferase TrmG/ribosomal RNA large subunit methyltransferase RlmN
rseB	3.807	Sigma-E factor regulatory protein RseB
rpoE	4.348	RNA polymerase sigma factor RpoE
smpB	6.193	SsrA-binding protein
STM2693	4.174	tmRNA, 10Sa RNA, ssrA
mutS	8.879	DNA mismatch repair protein MutS
yghJ	21.837	Type I-E CRISPR-associated protein Cas7/Cse4/CasC
yraO	39.232	Putative phosphoheptose isomerase
yraP	28.121	Osmotically-inducible protein OsmY
rpsI	5.619	30S ribosomal protein S9
rpmD	3.527	50S ribosomal protein L30
rplB	5.684	50S ribosomal protein L2
rplC	3.978	50S ribosomal protein L3
prlC	3.032	Oligopeptidase A
yiaD	9.437	OmpA family lipoprotein
STM4032.2N	3.200	Type II TA system; SehC toxin
STM4077	5.671	Autoinducer 2 ABC transporter substrate-binding protein LsrB precursor
tufB	17.997	Translation elongation factor Tu
rplA	4.261	50S ribosomal protein L1
rplL	167.151	50S ribosomal protein L7/L12
yjfN	3.162	Putative inner membrane protein
STM4495	4.984	type II restriction enzyme methylase subunit
hsdM	7.422	DNA methylase M
hsdR	8.233	Type I restriction-modification system endonuclease
STM0082	-64.252	SsrB-regulated factor N; DUF1471-containing protein
rnhB	-10.719	ribonuclease HII
rnhA	-5.342	degrades RNA of DNA-RNA hybrids
STM0294	-5.438	phosphotriesterase
STM0359	-248.696	Salmonella secreted substrate A, SssA
STM0551	-77.484	diguanylate cyclase
STM0561	-63.092	ATP-binding protein
STM0721	-63.718	glycosyltransferase family 1 protein
dps	-2.895	DNA starvation/stationary phase protection protein Dps
STM0854	-12.133	CoA ester lyase
STM0947	-6.204	transposase

Hypothetical, unknown function, pseudogenes (continued)		
STM2680	7.332	Hypothetical protein
STM2746	25.634	Putative excinuclease ATPase subunit
STM2747	19.143	Putative cytoplasmic protein
STM2762	20.690	Putative inner membrane protein
STM3031	5.181	Hypothetical protein
yhbU	25.119	Putative protease
yhhL	5.476	Putative inner membrane protein
STM3845	5.078	Hypothetical protein
STM3846.s	4.302	
STM05910	44.121	
STM4305.S	3.395	
ytfJ	5.653	Hypothetical protein
creA	3.942	Hypothetical protein
STM0032	-4.205	putative arylsulfatase
STM0034	-14.224	hypothetical protein
STM0100	-4.852	DUF4751 domain-containing protein
STM0271	-4.417	hypothetical protein
STM0305	-39.953	hypothetical protein
STM0333	-5.951	putative LysR family transcriptional regulator
STM0334	-6.356	putative cytoplasmic protein
STM0437	-5.618	hypothetical protein
STM0438	-3.928	putative TPR repeat protein
ybaY	-5.437	hypothetical protein
ylaC	-3.672	hypothetical protein
STM0497	-5.115	hypothetical protein
STM0699	-10.821	5-nitroimidazole antibiotic resistance protein
STM0719	-151.463	putative UDP-galactopyranose mutase
STM0759	-5.841	Uncharacterized protein ybgS
STM0777	-7.062	hypothetical protein
STM0809	-5.376	putative inner membrane protein
STM0810	-93.068	hypothetical protein
STM0839	-7.483	putative inner membrane protein
STM0860	-3.726	hypothetical protein
STM0861	-4.887	putative dehydrogenase
STM1077	-5.215	hypothetical protein
yccJ	-3.166	hypothetical protein
ycdF	-45.790	pseudo
ycfH	-3.260	putative metal-dependent hydrolase
STM1261	-20.539	four-helix bundle copper-binding protein
STM1263	-104.998	putative periplasmic protein
STM1380	-6.746	Putative hydrolase or acyltransferase
STM1389	-3.687	hypothetical protein
ydhJ	-6.192	putative multidrug resistance efflux pump
ydhI	-4.521	hypothetical protein
ydgC	-43.639	hypothetical protein
STM1513	-31.004	stress-induced acidophilic repeat motif-containing protein
STM1549	-4.787	hypothetical protein
STM1552	-6.392	putative cytoplasmic protein
STM1553	-9.164	pseudo
STM1561	-6.769	hypothetical protein
STM1621	-37.221	putative periplasmic protein
STM1624	-5.962	DUF1338 domain-containing protein
STM1665	-9.428	hypothetical protein
STM1666	-42.458	pseudo

Other functions (continued)		
STM0948	-12.094	transposase
zapC	-4.037	cell division protein ZapC
ymfC	-4.796	23S rRNA pseudouridine synthase E
ydeI	-19.949	TIGR00156 family protein
rpsV	-100.315	30S ribosomal subunit S22
dbpA	-3.513	ATP-dependent RNA helicase DbpA
STM1678	-9.495	aromatic alcohol reductase
STM1731	-54.078	Mn-containing catalase
umuD	-22.545	DNA polymerase V subunit UmuD
elaB	-7.832	putative inner membrane protein
csiE	-5.856	stationary phase inducible protein CsiE
glrK	-4.178	two-component system, NtrC family, sensor histidine kinase GlrK
STM2689	-8.223	Biofilm-associated protein, Bap
ygaU	-9.383	peptidoglycan-binding protein LysM
csrB	-6.303	ncRNA
yhcM	-5.453	cell division protein ZapE
hopD	-106.773	prepilin peptidase
dinF	-10.757	DNA-damage-inducible protein F
STM4242	-3.706	conjugal transfer protein
hfq	-148.439	RNA chaperone Hfq

Hypothetical, unknown and pseudogenes (continued)		
yciF	-36.204	hypothetical protein
yciE	-86.602	hypothetical protein
STM1810	-60.893	hypothetical protein
STM1827.S	-4.710	
STM1851	-17.620	hypothetical protein
STM1988.S	-12.234	
STM1999	-7.567	hypothetical protein
yehR	-55.175	hypothetical protein
STM2156A	-7.535	hypothetical protein
STM2376	-45.715	hypothetical protein
yfdC	-6.820	hypothetical protein
STM2434	-48.033	hypothetical protein
STM2508	-8.893	putative cytoplasmic protein
STM05520	-5.547	
STM2789	-8.916	putative cytoplasmic protein
STM2954.1n	-5.780	
ygdI	-15.115	hypothetical protein
STM3085	-5.749	hypothetical protein
yqjC	-3.039	hypothetical protein
STM3270	-63.430	hypothetical protein
yhbE	-6.493	pseudo
yrdA	-3.789	gamma carbonic anhydrase family protein
STM0348	-6.406	hypothetical protein
STM3752	-6.971	hypothetical protein
STM3940	-58.874	hypothetical protein
STM3941	-15.483	hypothetical protein
STM3981	-7.928	putative cytoplasmic protein
STM4013.S	-28.027	
STM4015	-6.159	hypothetical protein
STM4192	-3.257	hypothetical protein
STM4197	-14.231	putative inner membrane protein
STM4219.S	-8.072	
yjbJ	-5.905	putative cytoplasmic protein
phnB	-6.549	VOC family protein
STM4309	-80.620	hypothetical protein
yjeJ	-4.639	hypothetical protein
STM4562	-14.594	putative inner membrane protein
**Phage/Prophage proteins**		
STM1054	5.407	Gifsy-2 prophage protein
STM1055	8.884	Gifsy-2 prophage protein
STM2709	21.951	Fels-2 prophage protein
mig-3	-4.871	phage tail protein
STM2235	-9.057	putative phage tail fiber protein
STM2585A	-56.599	Gifsy-1 prophage protein, PagK2
STM2593	-9.284	Gifsy-1 prophage protein
STM2694	-8.697	Fels-2 prophage protein

* Significant differences between the wild type and Δ*hfq* cultures grown in the control condition only were determined according to an FDR < 0.05 and a minimum logFC of 1 or -1 (2-fold increase or decrease in expression, respectively). LogFC values were converted to fold change. Red shading indicates upregulation in the mutant and blue shading downregulation.
