# Supplementary material for: Spaceflight Analogue Culture Enhances the Host-Pathogen Interaction Between Salmonella and a 3-D Biomimetic Intestinal Co-Culture Model
Source: Front Cell Infect Microbiol. 2022 May 31;12:705647. doi: 10.3389/fcimb.2022.705647 (PMC9195300; doi:10.3389/fcimb.2022.705647)
Supplement: Supplementary file 12 [file Table_7.pdf]

## Supplementary Table 7. Host GO Biological Process and KEGG pathway enrichment analysis during invasion*

| Category | Term | Count | Percent | P-value | Fold Enrichment | Benjamini |
|---|---|---|---|---|---|---|
| **Wild Type: LSMMG-infected vs. uninfected host cells** | | | | | | |
| ***GO Biological Process*** | | | | | | |
| GOTERM_BP_DIRECT | SRP-dependent cotranslational protein targeting to membrane | 32 | 6.2 | 1.00E-26 | 13.6 | 1.90E-23 |
| GOTERM_BP_DIRECT | nuclear-transcribed mRNA catabolic process, nonsense-mediated decay | 33 | 6.4 | 2.00E-24 | 11.1 | 1.90E-21 |
| GOTERM_BP_DIRECT | viral transcription | 32 | 6.2 | 4.20E-24 | 11.4 | 2.60E-21 |
| GOTERM_BP_DIRECT | translational initiation | 33 | 6.4 | 2.30E-22 | 9.6 | 1.10E-19 |
| GOTERM_BP_DIRECT | rRNA processing | 38 | 7.4 | 7.40E-21 | 7.1 | 2.80E-18 |
| GOTERM_BP_DIRECT | translation | 36 | 7 | 1.20E-16 | 5.7 | 3.50E-14 |
| GOTERM_BP_DIRECT | nucleosome assembly | 15 | 2.9 | 1.60E-06 | 5 | 4.30E-04 |
| GOTERM_BP_DIRECT | chromatin silencing | 9 | 1.7 | 1.30E-05 | 8 | 3.10E-03 |
| GOTERM_BP_DIRECT | positive regulation of gene expression, epigenetic | 10 | 1.9 | 2.20E-05 | 6.4 | 4.50E-03 |
| GOTERM_BP_DIRECT | mitochondrial respiratory chain complex I assembly | 10 | 1.9 | 2.50E-05 | 6.3 | 4.60E-03 |
| GOTERM_BP_DIRECT | mitochondrial electron transport, NADH to ubiquinone | 9 | 1.7 | 2.60E-05 | 7.3 | 4.40E-03 |
| GOTERM_BP_DIRECT | cytoplasmic translation | 7 | 1.4 | 2.80E-05 | 11.2 | 4.30E-03 |
| GOTERM_BP_DIRECT | inflammatory response | 25 | 4.8 | 3.10E-05 | 2.6 | 4.40E-03 |
| GOTERM_BP_DIRECT | antibacterial humoral response | 8 | 1.5 | 9.90E-05 | 7.3 | 1.30E-02 |
| GOTERM_BP_DIRECT | mitochondrial electron transport, cytochrome c to oxygen | 6 | 1.2 | 1.10E-04 | 12 | 1.30E-02 |
| GOTERM_BP_DIRECT | hydrogen ion transmembrane transport | 9 | 1.7 | 1.30E-04 | 5.9 | 1.50E-02 |
| GOTERM_BP_DIRECT | cell-cell adhesion | 19 | 3.7 | 1.70E-04 | 2.8 | 1.80E-02 |
| GOTERM_BP_DIRECT | chromatin silencing at rDNA | 7 | 1.4 | 2.80E-04 | 7.6 | 2.90E-02 |
| GOTERM_BP_DIRECT | innate immune response in mucosa | 6 | 1.2 | 3.30E-04 | 9.6 | 3.20E-02 |
| GOTERM_BP_DIRECT | telomere organization | 6 | 1.2 | 4.80E-04 | 8.9 | 4.40E-02 |
| GOTERM_BP_DIRECT | cellular response to lipopolysaccharide | 11 | 2.1 | 5.30E-04 | 3.9 | 4.60E-02 |
| GOTERM_BP_DIRECT | ribosomal small subunit biogenesis | 5 | 1 | 5.50E-04 | 12.5 | 4.50E-02 |
| ***KEGG Pathway*** | | | | | | |
| KEGG_PATHWAY | Ribosome | 35 | 6.8 | 3.60E-21 | 7.7 | 8.00E-19 |
| KEGG_PATHWAY | Oxidative phosphorylation | 24 | 4.6 | 5.40E-11 | 5.4 | 5.90E-09 |
| KEGG_PATHWAY | Non-alcoholic fatty liver disease (NAFLD) | 24 | 4.6 | 7.50E-10 | 4.7 | 5.50E-08 |
| KEGG_PATHWAY | Alzheimer's disease | 24 | 4.6 | 6.40E-09 | 4.3 | 3.50E-07 |
| KEGG_PATHWAY | Systemic lupus erythematosus | 21 | 4.1 | 1.50E-08 | 4.7 | 6.50E-07 |
| KEGG_PATHWAY | Alcoholism | 24 | 4.6 | 1.80E-08 | 4 | 6.50E-07 |
| KEGG_PATHWAY | Huntington's disease | 23 | 4.4 | 3.50E-07 | 3.6 | 1.10E-05 |
| KEGG_PATHWAY | Parkinson's disease | 19 | 3.7 | 9.80E-07 | 4 | 2.70E-05 |
| KEGG_PATHWAY | TNF signaling pathway | 16 | 3.1 | 2.30E-06 | 4.5 | 5.50E-05 |
| KEGG_PATHWAY | Legionellosis | 10 | 1.9 | 6.40E-05 | 5.5 | 1.40E-03 |
| KEGG_PATHWAY | Viral carcinogenesis | 18 | 3.5 | 4.70E-04 | 2.6 | 9.30E-03 |
| KEGG_PATHWAY | Cardiac muscle contraction | 10 | 1.9 | 8.20E-04 | 4 | 1.50E-02 |
| KEGG_PATHWAY | NF-kappa B signaling pathway | 10 | 1.9 | 2.40E-03 | 3.4 | 3.90E-02 |
| KEGG_PATHWAY | NOD-like receptor signaling pathway | 8 | 1.5 | 2.40E-03 | 4.3 | 3.80E-02 |
| KEGG_PATHWAY | Epstein-Barr virus infection | 12 | 2.3 | 2.50E-03 | 2.9 | 3.60E-02 |
| **Wild Type: Control-infected vs. uninfected host cells** | | | | | | |
| ***GO Biological Process*** | | | | | | |
| GOTERM_BP_DIRECT | SRP-dependent cotranslational protein targeting to membrane | 28 | 8.4 | 8.30E-27 | 18.6 | 1.20E-23 |
| GOTERM_BP_DIRECT | viral transcription | 27 | 8.1 | 3.20E-23 | 15 | 2.40E-20 |
| GOTERM_BP_DIRECT | nuclear-transcribed mRNA catabolic process, nonsense-mediated decay | 27 | 8.1 | 1.70E-22 | 14.2 | 8.40E-20 |
| GOTERM_BP_DIRECT | translational initiation | 27 | 8.1 | 7.70E-21 | 12.3 | 2.90E-18 |
| GOTERM_BP_DIRECT | rRNA processing | 28 | 8.4 | 7.30E-17 | 8.2 | 3.30E-14 |
| GOTERM_BP_DIRECT | translation | 29 | 8.7 | 7.00E-16 | 7.2 | 1.60E-13 |
| GOTERM_BP_DIRECT | chromatin silencing | 10 | 3 | 3.20E-08 | 13.9 | 6.70E-06 |
| GOTERM_BP_DIRECT | inflammatory response | 23 | 6.9 | 2.00E-07 | 3.8 | 3.60E-05 |
| GOTERM_BP_DIRECT | cell chemotaxis | 9 | 2.7 | 8.70E-06 | 8.6 | 1.40E-03 |
| GOTERM_BP_DIRECT | cytoplasmic translation | 6 | 1.8 | 4.10E-05 | 15 | 6.00E-03 |

| Category | Term | Count | Percent | P-value | Fold Enrichment | Benjamini |
|---|---|---|---|---|---|---|
| ***GO Biological Process (continued)*** | | | | | | |
| GOTERM_BP_DIRECT | cellular response to lipopolysaccharide | 10 | 3 | 8.30E-05 | 5.5 | 1.10E-02 |
| GOTERM_BP_DIRECT | chemokine-mediated signaling pathway | 8 | 2.4 | 1.40E-04 | 7 | 1.70E-02 |
| GOTERM_BP_DIRECT | chemotaxis | 10 | 3 | 1.50E-04 | 5.1 | 1.70E-02 |
| GOTERM_BP_DIRECT | cell-cell adhesion | 14 | 4.2 | 4.30E-04 | 3.2 | 4.40E-02 |
| GOTERM_BP_DIRECT | immune response | 18 | 5.4 | 4.50E-04 | 2.7 | 4.30E-02 |
| ***KEGG Pathway*** | | | | | | |
| KEGG_PATHWAY | Ribosome | 29 | 8.7 | 1.60E-20 | 9.9 | 3.00E-18 |
| KEGG_PATHWAY | Alcoholism | 19 | 5.7 | 3.10E-08 | 5 | 3.00E-06 |
| KEGG_PATHWAY | Systemic lupus erythematosus | 16 | 4.8 | 1.30E-07 | 5.5 | 8.70E-06 |
| KEGG_PATHWAY | Oxidative phosphorylation | 15 | 4.5 | 7.70E-07 | 5.2 | 3.70E-05 |
| KEGG_PATHWAY | TNF signaling pathway | 13 | 3.9 | 2.50E-06 | 5.6 | 9.80E-05 |
| KEGG_PATHWAY | Alzheimer's disease | 15 | 4.5 | 1.20E-05 | 4.1 | 4.00E-04 |
| KEGG_PATHWAY | Legionellosis | 9 | 2.7 | 1.60E-05 | 7.7 | 4.50E-04 |
| KEGG_PATHWAY | NOD-like receptor signaling pathway | 9 | 2.7 | 2.10E-05 | 7.5 | 5.20E-04 |
| KEGG_PATHWAY | Non-alcoholic fatty liver disease (NAFLD) | 13 | 3.9 | 8.50E-05 | 4 | 1.80E-03 |
| KEGG_PATHWAY | Huntington's disease | 14 | 4.2 | 2.20E-04 | 3.4 | 4.20E-03 |
| KEGG_PATHWAY | Cytokine-cytokine receptor interaction | 15 | 4.5 | 6.50E-04 | 2.9 | 1.20E-02 |
| KEGG_PATHWAY | Parkinson's disease | 11 | 3.3 | 8.70E-04 | 3.6 | 1.40E-02 |
| KEGG_PATHWAY | Pertussis | 8 | 2.4 | 1.00E-03 | 5 | 1.50E-02 |
| KEGG_PATHWAY | Viral carcinogenesis | 13 | 3.9 | 1.40E-03 | 2.9 | 1.90E-02 |
| KEGG_PATHWAY | Salmonella infection | 8 | 2.4 | 1.90E-03 | 4.5 | 2.40E-02 |
| **Δ*hfq* mutant: LSMMG-infected vs. uninfected host cells** | | | | | | |
| ***GO Biological Process*** | | | | | | |
| GOTERM_BP_DIRECT | inflammatory response | 42 | 19.5 | 1.40E-32 | 12 | 1.70E-29 |
| GOTERM_BP_DIRECT | immune response | 26 | 12.1 | 9.70E-14 | 6.7 | 5.70E-11 |
| GOTERM_BP_DIRECT | chemotaxis | 13 | 6 | 1.30E-09 | 11.5 | 4.90E-07 |
| GOTERM_BP_DIRECT | response to lipopolysaccharide | 14 | 6.5 | 3.60E-09 | 9.2 | 1.10E-06 |
| GOTERM_BP_DIRECT | cellular response to lipopolysaccharide | 12 | 5.6 | 7.00E-09 | 11.5 | 1.70E-06 |
| GOTERM_BP_DIRECT | chemokine-mediated signaling pathway | 10 | 4.7 | 1.70E-08 | 15.3 | 3.30E-06 |
| GOTERM_BP_DIRECT | I-kappaB kinase/NF-kappaB signaling | 9 | 4.2 | 7.10E-08 | 16.3 | 1.20E-05 |
| GOTERM_BP_DIRECT | cell chemotaxis | 9 | 4.2 | 1.40E-07 | 15 | 2.00E-05 |
| GOTERM_BP_DIRECT | positive regulation of transcription from RNA polymerase II promoter | 27 | 12.6 | 9.20E-07 | 3 | 1.20E-04 |
| GOTERM_BP_DIRECT | epidermis development | 9 | 4.2 | 1.10E-06 | 11.5 | 1.30E-04 |
| GOTERM_BP_DIRECT | keratinocyte differentiation | 8 | 3.7 | 6.10E-06 | 11.4 | 6.60E-04 |
| GOTERM_BP_DIRECT | neutrophil chemotaxis | 7 | 3.3 | 3.10E-05 | 11.5 | 3.10E-03 |
| GOTERM_BP_DIRECT | cellular response to interleukin-1 | 7 | 3.3 | 4.70E-05 | 10.7 | 4.30E-03 |
| GOTERM_BP_DIRECT | regulation of cell proliferation | 10 | 4.7 | 5.30E-05 | 5.9 | 4.50E-03 |
| GOTERM_BP_DIRECT | keratinization | 6 | 2.8 | 7.60E-05 | 13.5 | 5.90E-03 |
| GOTERM_BP_DIRECT | positive regulation of lipid storage | 4 | 1.9 | 8.70E-05 | 43.3 | 6.40E-03 |
| GOTERM_BP_DIRECT | peptide cross-linking | 6 | 2.8 | 9.20E-05 | 13 | 6.40E-03 |
| GOTERM_BP_DIRECT | response to cytokine | 6 | 2.8 | 1.10E-04 | 12.5 | 7.30E-03 |
| GOTERM_BP_DIRECT | cell-cell signaling | 11 | 5.1 | 1.20E-04 | 4.7 | 7.40E-03 |
| GOTERM_BP_DIRECT | signal transduction | 25 | 11.6 | 1.50E-04 | 2.3 | 8.60E-03 |
| GOTERM_BP_DIRECT | cytokine-mediated signaling pathway | 8 | 3.7 | 2.10E-04 | 6.6 | 1.10E-02 |
| GOTERM_BP_DIRECT | positive regulation of NF-kappaB transcription factor activity | 8 | 3.7 | 2.30E-04 | 6.5 | 1.20E-02 |
| GOTERM_BP_DIRECT | negative regulation of extrinsic apoptotic signaling pathway via death domain receptors | 5 | 2.3 | 2.30E-04 | 16.4 | 1.20E-02 |
| GOTERM_BP_DIRECT | regulation of inflammatory response | 6 | 2.8 | 2.80E-04 | 10.3 | 1.40E-02 |
| GOTERM_BP_DIRECT | negative regulation of cell proliferation | 13 | 6 | 3.00E-04 | 3.6 | 1.40E-02 |
| GOTERM_BP_DIRECT | NIK/NF-kappaB signaling | 6 | 2.8 | 3.50E-04 | 9.8 | 1.60E-02 |
| GOTERM_BP_DIRECT | negative regulation of I-kappaB kinase/NF-kappaB signaling | 5 | 2.3 | 4.80E-04 | 13.5 | 2.10E-02 |
| GOTERM_BP_DIRECT | toll-like receptor 4 signaling pathway | 4 | 1.9 | 5.60E-04 | 24.1 | 2.30E-02 |
| GOTERM_BP_DIRECT | positive regulation of angiogenesis | 7 | 3.3 | 6.70E-04 | 6.6 | 2.70E-02 |
| GOTERM_BP_DIRECT | positive regulation of I-kappaB kinase/NF-kappaB signaling | 8 | 3.7 | 7.20E-04 | 5.4 | 2.80E-02 |
| GOTERM_BP_DIRECT | negative regulation of inflammatory response | 6 | 2.8 | 7.90E-04 | 8.2 | 3.00E-02 |
| GOTERM_BP_DIRECT | negative regulation of apoptotic process | 13 | 6 | 1.00E-03 | 3.1 | 3.60E-02 |

| Category | Term | Count | Percent | P-value | Fold Enrichment | Benjamini |
|---|---|---|---|---|---|---|
| ***GO Biological Process (continued)*** | | | | | | |
| GOTERM_BP_DIRECT | positive regulation of neutrophil chemotaxis | 4 | 1.9 | 1.00E-03 | 19.7 | 3.60E-02 |
| ***KEGG pathway*** | | | | | | |
| KEGG_PATHWAY | TNF signaling pathway | 21 | 9.8 | 2.00E-17 | 13.4 | 3.00E-15 |
| KEGG_PATHWAY | Cytokine-cytokine receptor interaction | 26 | 12.1 | 3.40E-15 | 7.3 | 2.50E-13 |
| KEGG_PATHWAY | NF-kappa B signaling pathway | 12 | 5.6 | 3.90E-08 | 9.4 | 1.90E-06 |
| KEGG_PATHWAY | Legionellosis | 10 | 4.7 | 6.20E-08 | 12.6 | 2.30E-06 |
| KEGG_PATHWAY | NOD-like receptor signaling pathway | 10 | 4.7 | 8.70E-08 | 12.2 | 2.60E-06 |
| KEGG_PATHWAY | Pertussis | 9 | 4.2 | 1.10E-05 | 8.2 | 2.80E-04 |
| KEGG_PATHWAY | Chemokine signaling pathway | 13 | 6 | 1.40E-05 | 4.8 | 3.00E-04 |
| KEGG_PATHWAY | Osteoclast differentiation | 11 | 5.1 | 1.80E-05 | 5.7 | 3.30E-04 |
| KEGG_PATHWAY | Rheumatoid arthritis | 9 | 4.2 | 3.70E-05 | 7 | 6.10E-04 |
| KEGG_PATHWAY | Measles | 10 | 4.7 | 1.20E-04 | 5.1 | 1.80E-03 |
| KEGG_PATHWAY | Salmonella infection | 8 | 3.7 | 1.90E-04 | 6.6 | 2.50E-03 |
| KEGG_PATHWAY | Influenza A | 11 | 5.1 | 2.00E-04 | 4.3 | 2.50E-03 |
| KEGG_PATHWAY | Hepatitis C | 9 | 4.2 | 6.60E-04 | 4.6 | 7.50E-03 |
| KEGG_PATHWAY | Amoebiasis | 8 | 3.7 | 8.40E-04 | 5.1 | 8.80E-03 |
| KEGG_PATHWAY | Jak-STAT signaling pathway | 9 | 4.2 | 1.20E-03 | 4.2 | 1.10E-02 |
| KEGG_PATHWAY | Hematopoietic cell lineage | 7 | 3.3 | 1.60E-03 | 5.5 | 1.50E-02 |
| KEGG_PATHWAY | Epstein-Barr virus infection | 8 | 3.7 | 1.90E-03 | 4.5 | 1.60E-02 |
| KEGG_PATHWAY | Transcriptional misregulation in cancer | 9 | 4.2 | 2.90E-03 | 3.7 | 2.30E-02 |
| KEGG_PATHWAY | Leishmaniasis | 6 | 2.8 | 3.60E-03 | 5.8 | 2.80E-02 |
| KEGG_PATHWAY | HTLV-I infection | 11 | 5.1 | 3.70E-03 | 2.9 | 2.70E-02 |
| KEGG_PATHWAY | Chagas disease (American trypanosomiasis) | 7 | 3.3 | 3.90E-03 | 4.6 | 2.70E-02 |
| KEGG_PATHWAY | Toll-like receptor signaling pathway | 7 | 3.3 | 4.30E-03 | 4.5 | 2.90E-02 |
| KEGG_PATHWAY | Malaria | 5 | 2.3 | 5.40E-03 | 6.9 | 3.40E-02 |
| **Δ*hfq* mutant: Control-infected vs. uninfected host cells** | | | | | | |
| ***GO Biological Process*** | | | | | | |
| GOTERM_BP_DIRECT | inflammatory response | 42 | 19.6 | 2.50E-32 | 11.9 | 3.20E-29 |
| GOTERM_BP_DIRECT | immune response | 25 | 11.7 | 1.00E-12 | 6.4 | 6.40E-10 |
| GOTERM_BP_DIRECT | response to lipopolysaccharide | 15 | 7 | 3.90E-10 | 9.8 | 1.70E-07 |
| GOTERM_BP_DIRECT | cell chemotaxis | 10 | 4.7 | 8.40E-09 | 16.5 | 2.60E-06 |
| GOTERM_BP_DIRECT | chemokine-mediated signaling pathway | 10 | 4.7 | 1.90E-08 | 15.1 | 4.70E-06 |
| GOTERM_BP_DIRECT | cellular response to lipopolysaccharide | 11 | 5.1 | 9.80E-08 | 10.4 | 2.10E-05 |
| GOTERM_BP_DIRECT | chemotaxis | 11 | 5.1 | 2.00E-07 | 9.6 | 3.60E-05 |
| GOTERM_BP_DIRECT | positive regulation of transcription from RNA polymerase II promoter | 27 | 12.6 | 1.20E-06 | 2.9 | 1.90E-04 |
| GOTERM_BP_DIRECT | I-kappaB kinase/NF-kappaB signaling | 8 | 3.7 | 1.30E-06 | 14.3 | 1.90E-04 |
| GOTERM_BP_DIRECT | cell-cell signaling | 13 | 6.1 | 4.50E-06 | 5.5 | 5.70E-04 |
| GOTERM_BP_DIRECT | regulation of cell proliferation | 11 | 5.1 | 9.10E-06 | 6.4 | 1.00E-03 |
| GOTERM_BP_DIRECT | regulation of inflammatory response | 7 | 3.3 | 2.60E-05 | 11.9 | 2.70E-03 |
| GOTERM_BP_DIRECT | cytokine-mediated signaling pathway | 9 | 4.2 | 3.10E-05 | 7.3 | 2.90E-03 |
| GOTERM_BP_DIRECT | neutrophil chemotaxis | 7 | 3.3 | 3.30E-05 | 11.3 | 3.00E-03 |
| GOTERM_BP_DIRECT | cellular response to interleukin-1 | 7 | 3.3 | 5.10E-05 | 10.5 | 4.20E-03 |
| GOTERM_BP_DIRECT | signal transduction | 26 | 12.1 | 6.80E-05 | 2.4 | 5.30E-03 |
| GOTERM_BP_DIRECT | keratinocyte differentiation | 7 | 3.3 | 7.50E-05 | 9.9 | 5.50E-03 |
| GOTERM_BP_DIRECT | negative regulation of cell proliferation | 14 | 6.5 | 8.50E-05 | 3.8 | 5.90E-03 |
| GOTERM_BP_DIRECT | positive regulation of lipid storage | 4 | 1.9 | 9.00E-05 | 42.8 | 5.90E-03 |
| GOTERM_BP_DIRECT | negative regulation of inflammatory response | 7 | 3.3 | 9.30E-05 | 9.5 | 5.80E-03 |
| GOTERM_BP_DIRECT | positive regulation of angiogenesis | 8 | 3.7 | 9.90E-05 | 7.4 | 5.90E-03 |
| GOTERM_BP_DIRECT | epidermis development | 7 | 3.3 | 1.40E-04 | 8.8 | 7.90E-03 |
| GOTERM_BP_DIRECT | NIK/NF-kappaB signaling | 6 | 2.8 | 3.70E-04 | 9.7 | 2.00E-02 |
| GOTERM_BP_DIRECT | negative regulation of I-kappaB kinase/NF-kappaB signaling | 5 | 2.3 | 5.00E-04 | 13.4 | 2.60E-02 |
| GOTERM_BP_DIRECT | cellular response to mechanical stimulus | 6 | 2.8 | 5.20E-04 | 9 | 2.60E-02 |
| GOTERM_BP_DIRECT | positive regulation of nitric oxide biosynthetic process | 5 | 2.3 | 6.70E-04 | 12.4 | 3.20E-02 |
| GOTERM_BP_DIRECT | positive regulation of gene expression | 10 | 4.7 | 7.80E-04 | 4.1 | 3.50E-02 |
| GOTERM_BP_DIRECT | regulation of transcription from RNA polymerase II promoter | 13 | 6.1 | 8.60E-04 | 3.2 | 3.80E-02 |

| Category | Term | Count | Percent | P-value | Fold Enrichment | Benjamini |
|---|---|---|---|---|---|---|
| ***GO Biological Process (continued)*** | | | | | | |
| GOTERM_BP_DIRECT | positive regulation of MAPK cascade | 6 | 2.8 | 9.40E-04 | 7.9 | 4.00E-02 |
| GOTERM_BP_DIRECT | keratinization | 5 | 2.3 | 1.00E-03 | 11.1 | 4.20E-02 |
| GOTERM_BP_DIRECT | positive regulation of neutrophil chemotaxis | 4 | 1.9 | 1.10E-03 | 19.4 | 4.20E-02 |
| GOTERM_BP_DIRECT | response to organic cyclic compound | 5 | 2.3 | 1.10E-03 | 10.9 | 4.20E-02 |
| GOTERM_BP_DIRECT | peptide cross-linking | 5 | 2.3 | 1.20E-03 | 10.7 | 4.40E-02 |
| GOTERM_BP_DIRECT | response to cytokine | 5 | 2.3 | 1.40E-03 | 10.3 | 4.90E-02 |
| ***KEGG Pathway*** | | | | | | |
| KEGG_PATHWAY | TNF signaling pathway | 22 | 10.3 | 1.90E-18 | 13.6 | 2.90E-16 |
| KEGG_PATHWAY | Cytokine-cytokine receptor interaction | 24 | 11.2 | 6.20E-13 | 6.5 | 4.70E-11 |
| KEGG_PATHWAY | NF-kappa B signaling pathway | 12 | 5.6 | 5.40E-08 | 9.1 | 2.70E-06 |
| KEGG_PATHWAY | Legionellosis | 10 | 4.7 | 8.10E-08 | 12.2 | 3.10E-06 |
| KEGG_PATHWAY | NOD-like receptor signaling pathway | 9 | 4.2 | 1.50E-06 | 10.6 | 4.60E-05 |
| KEGG_PATHWAY | Osteoclast differentiation | 12 | 5.6 | 3.50E-06 | 6.1 | 8.90E-05 |
| KEGG_PATHWAY | Pertussis | 9 | 4.2 | 1.40E-05 | 7.9 | 3.10E-04 |
| KEGG_PATHWAY | Measles | 11 | 5.1 | 2.70E-05 | 5.5 | 5.10E-04 |
| KEGG_PATHWAY | Salmonella infection | 9 | 4.2 | 3.00E-05 | 7.2 | 5.10E-04 |
| KEGG_PATHWAY | Rheumatoid arthritis | 9 | 4.2 | 4.60E-05 | 6.8 | 7.00E-04 |
| KEGG_PATHWAY | Influenza A | 12 | 5.6 | 5.20E-05 | 4.6 | 7.20E-04 |
| KEGG_PATHWAY | Leishmaniasis | 8 | 3.7 | 8.30E-05 | 7.5 | 1.10E-03 |
| KEGG_PATHWAY | Chemokine signaling pathway | 12 | 5.6 | 9.60E-05 | 4.3 | 1.10E-03 |
| KEGG_PATHWAY | Amoebiasis | 9 | 4.2 | 1.70E-04 | 5.6 | 1.90E-03 |
| KEGG_PATHWAY | Hematopoietic cell lineage | 8 | 3.7 | 3.00E-04 | 6.1 | 3.00E-03 |
| KEGG_PATHWAY | Epstein-Barr virus infection | 9 | 4.2 | 4.50E-04 | 4.9 | 4.30E-03 |
| KEGG_PATHWAY | Hepatitis C | 9 | 4.2 | 8.00E-04 | 4.5 | 7.20E-03 |
| KEGG_PATHWAY | Transcriptional misregulation in cancer | 10 | 4.7 | 8.40E-04 | 4 | 7.10E-03 |
| KEGG_PATHWAY | Toll-like receptor signaling pathway | 8 | 3.7 | 1.00E-03 | 5 | 7.90E-03 |
| KEGG_PATHWAY | Jak-STAT signaling pathway | 9 | 4.2 | 1.40E-03 | 4.1 | 1.10E-02 |
| KEGG_PATHWAY | Chagas disease (American trypanosomiasis) | 7 | 3.3 | 4.50E-03 | 4.5 | 3.20E-02 |
| KEGG_PATHWAY | Malaria | 5 | 2.3 | 6.00E-03 | 6.7 | 4.10E-02 |

[*] GO Biological process and KEGG pathway enrichment analyses were performed using DAVID 6.8 using a threshold count of 2 and an EASE score of 0.05. Only terms and pathways with Benjamini-Hochberg values that fall below 0.05 are shown.
