# Supplementary material for: Spaceflight Analogue Culture Enhances the Host-Pathogen Interaction Between Salmonella and a 3-D Biomimetic Intestinal Co-Culture Model
Source: Front Cell Infect Microbiol. 2022 May 31;12:705647. doi: 10.3389/fcimb.2022.705647 (PMC9195300; doi:10.3389/fcimb.2022.705647)
Supplement: Supplementary file 13 [file Table_8.pdf]

Supplementary Table 8. Host GO Biological Process and KEGG pathway analysis for wild type-infected cells during survival*

Category	Term	Count	Percent	P-value	Fold Enrichment	Benjamini
**Wild Type: LSMMG-infected vs. control-infected, upregulated genes only**						
GO Biological Process						
GOTERM_BP_DIRECT	cell division	35	3.5	6.50E-07	2.6	1.50E-03
GOTERM_BP_DIRECT	sister chromatid cohesion	16	1.6	8.50E-06	4.1	9.60E-03
GOTERM_BP_DIRECT	DNA synthesis involved in DNA repair	9	0.9	4.20E-05	6.7	3.10E-02
KEGG Pathway						
KEGG_PATHWAY	Spliceosome	17	1.7	5.10E-05	3.3	1.20E-02
KEGG_PATHWAY	RNA transport	18	1.8	3.40E-04	2.7	4.00E-02
KEGG_PATHWAY	Ribosome biogenesis in eukaryotes	12	1.2	5.00E-04	3.5	3.80E-02
**Wild Type: LSMMG-infected vs. control-infected, downregulated genes only**						
GO Biological Process						
GOTERM_BP_DIRECT	SRP-dependent cotranslational protein targeting to membrane	24	1.9	3.50E-09	4.3	1.30E-05
GOTERM_BP_DIRECT	nuclear-transcribed mRNA catabolic process, nonsense-mediated decay	26	2	2.10E-08	3.7	3.70E-05
GOTERM_BP_DIRECT	viral transcription	25	2	2.70E-08	3.8	3.20E-05
GOTERM_BP_DIRECT	translation	37	2.9	7.70E-07	2.5	6.90E-04
GOTERM_BP_DIRECT	cell-cell adhesion	38	3	1.50E-06	2.4	1.10E-03
GOTERM_BP_DIRECT	translational initiation	24	1.9	4.80E-06	3	2.90E-03
GOTERM_BP_DIRECT	chromatin silencing	13	1	7.60E-06	4.9	3.90E-03
KEGG Pathway						
KEGG_PATHWAY	Ribosome	29	2.3	1.80E-08	3.4	4.70E-06
KEGG_PATHWAY	Axon guidance	20	1.6	3.60E-04	2.5	4.60E-02
KEGG_PATHWAY	Huntington's disease	26	2	4.10E-04	2.1	3.60E-02
KEGG_PATHWAY	Oxidative phosphorylation	20	1.6	6.50E-04	2.4	4.20E-02
KEGG_PATHWAY	Alcoholism	24	1.9	7.20E-04	2.1	3.80E-02

* GO Biological process and KEGG pathway enrichment analyses were performed using DAVID 6.8 using a threshold count of 2 and an EASE score of 0.05. Only terms and pathways with Benjamini-Hochberg values less than 0.05 are shown.
