# Supplementary material for: Spaceflight Analogue Culture Enhances the Host-Pathogen Interaction Between Salmonella and a 3-D Biomimetic Intestinal Co-Culture Model
Source: Front Cell Infect Microbiol. 2022 May 31;12:705647. doi: 10.3389/fcimb.2022.705647 (PMC9195300; doi:10.3389/fcimb.2022.705647)
Supplement: Supplementary file 14 [file Table_9.pdf]

**Supplementary Table 9. GO Biological Process and KEGG pathway analysis for Δ*hfq*-infected cells during survival***

| Category | Term | Count | Percent | P-value | Fold Enrichment | Benjamini |
|---|---|---|---|---|---|---|
| **LSMMG-infected vs. uninfected** | | | | | | |
| ***GO Biological Process*** | | | | | | |
| GOTERM_BP_DIRECT | inflammatory response | 25 | 29.4 | 3.70E-21 | 14 | 2.70E-18 |
| GOTERM_BP_DIRECT | response to lipopolysaccharide | 12 | 14.1 | 2.40E-10 | 15.6 | 9.00E-08 |
| GOTERM_BP_DIRECT | immune response | 16 | 18.8 | 7.80E-10 | 8.1 | 1.90E-07 |
| GOTERM_BP_DIRECT | chemokine-mediated signaling pathway | 8 | 9.4 | 3.70E-08 | 24 | 6.90E-06 |
| GOTERM_BP_DIRECT | cellular response to lipopolysaccharide | 9 | 10.6 | 5.20E-08 | 16.9 | 7.70E-06 |
| GOTERM_BP_DIRECT | cell chemotaxis | 7 | 8.2 | 5.50E-07 | 22.9 | 6.80E-05 |
| GOTERM_BP_DIRECT | neutrophil chemotaxis | 7 | 8.2 | 6.00E-07 | 22.5 | 6.40E-05 |
| GOTERM_BP_DIRECT | keratinization | 6 | 7.1 | 2.80E-06 | 26.6 | 2.60E-04 |
| GOTERM_BP_DIRECT | innate immune response | 12 | 14.1 | 4.50E-06 | 5.9 | 3.70E-04 |
| GOTERM_BP_DIRECT | plasminogen activation | 4 | 4.7 | 7.90E-06 | 94.5 | 5.90E-04 |
| GOTERM_BP_DIRECT | negative regulation of endothelial cell apoptotic process | 5 | 5.9 | 8.10E-06 | 38 | 5.50E-04 |
| GOTERM_BP_DIRECT | regulation of inflammatory response | 6 | 7.1 | 1.10E-05 | 20.2 | 6.70E-04 |
| GOTERM_BP_DIRECT | positive regulation of vasoconstriction | 5 | 5.9 | 1.40E-05 | 33.2 | 8.00E-04 |
| GOTERM_BP_DIRECT | negative regulation of extrinsic apoptotic signaling pathway via death domain receptors | 5 | 5.9 | 1.60E-05 | 32.2 | 8.40E-04 |
| GOTERM_BP_DIRECT | cellular response to interleukin-1 | 6 | 7.1 | 1.90E-05 | 18 | 9.60E-04 |
| GOTERM_BP_DIRECT | chemotaxis | 7 | 8.2 | 2.20E-05 | 12.2 | 1.00E-03 |
| GOTERM_BP_DIRECT | wound healing | 6 | 7.1 | 3.50E-05 | 15.9 | 1.50E-03 |
| GOTERM_BP_DIRECT | extracellular matrix organization | 8 | 9.4 | 3.50E-05 | 8.7 | 1.40E-03 |
| GOTERM_BP_DIRECT | peptide cross-linking | 5 | 5.9 | 8.40E-05 | 21.3 | 3.30E-03 |
| GOTERM_BP_DIRECT | fibrinolysis | 4 | 4.7 | 1.20E-04 | 40.5 | 4.50E-03 |
| GOTERM_BP_DIRECT | induction of bacterial agglutination | 3 | 3.5 | 1.30E-04 | 159.4 | 4.50E-03 |
| GOTERM_BP_DIRECT | blood coagulation, fibrin clot formation | 3 | 3.5 | 1.30E-04 | 159.4 | 4.50E-03 |
| GOTERM_BP_DIRECT | positive regulation of neutrophil chemotaxis | 4 | 4.7 | 1.40E-04 | 38.6 | 4.70E-03 |
| GOTERM_BP_DIRECT | cellular response to tumor necrosis factor | 6 | 7.1 | 1.60E-04 | 11.6 | 5.10E-03 |
| GOTERM_BP_DIRECT | regulation of cell proliferation | 7 | 8.2 | 2.20E-04 | 8 | 6.80E-03 |
| GOTERM_BP_DIRECT | negative regulation of endopeptidase activity | 6 | 7.1 | 2.50E-04 | 10.5 | 7.30E-03 |
| GOTERM_BP_DIRECT | keratinocyte differentiation | 5 | 5.9 | 4.30E-04 | 14 | 1.20E-02 |
| GOTERM_BP_DIRECT | positive regulation of peptide hormone secretion | 3 | 3.5 | 5.90E-04 | 79.7 | 1.60E-02 |
| GOTERM_BP_DIRECT | positive regulation of protein secretion | 4 | 4.7 | 6.20E-04 | 23.6 | 1.60E-02 |
| GOTERM_BP_DIRECT | epidermis development | 5 | 5.9 | 6.60E-04 | 12.5 | 1.70E-02 |
| GOTERM_BP_DIRECT | response to molecule of bacterial origin | 3 | 3.5 | 7.50E-04 | 70.9 | 1.80E-02 |
| GOTERM_BP_DIRECT | acute-phase response | 4 | 4.7 | 7.80E-04 | 21.8 | 1.90E-02 |
| GOTERM_BP_DIRECT | signal transduction | 15 | 17.6 | 8.30E-04 | 2.7 | 1.90E-02 |
| GOTERM_BP_DIRECT | positive regulation of lipid storage | 3 | 3.5 | 9.40E-04 | 63.8 | 2.10E-02 |
| GOTERM_BP_DIRECT | positive regulation of nitric oxide biosynthetic process | 4 | 4.7 | 1.00E-03 | 19.8 | 2.20E-02 |
| GOTERM_BP_DIRECT | positive regulation of heterotypic cell-cell adhesion | 3 | 3.5 | 1.10E-03 | 58 | 2.40E-02 |
| GOTERM_BP_DIRECT | positive regulation of ERK1 and ERK2 cascade | 6 | 7.1 | 1.30E-03 | 7.3 | 2.70E-02 |
| GOTERM_BP_DIRECT | platelet degranulation | 5 | 5.9 | 1.30E-03 | 10.3 | 2.70E-02 |
| GOTERM_BP_DIRECT | protein polymerization | 3 | 3.5 | 1.60E-03 | 49.1 | 3.10E-02 |
| ***KEGG Pathway*** | | | | | | |
| KEGG_PATHWAY | TNF signaling pathway | 12 | 14.1 | 1.70E-10 | 15.1 | 2.00E-08 |
| KEGG_PATHWAY | Legionellosis | 9 | 10.6 | 3.50E-09 | 22.5 | 2.00E-07 |
| KEGG_PATHWAY | Cytokine-cytokine receptor interaction | 13 | 15.3 | 1.10E-07 | 7.2 | 4.00E-06 |
| KEGG_PATHWAY | NF-kappa B signaling pathway | 9 | 10.6 | 1.60E-07 | 14 | 4.70E-06 |
| KEGG_PATHWAY | NOD-like receptor signaling pathway | 7 | 8.2 | 2.70E-06 | 16.9 | 6.10E-05 |
| KEGG_PATHWAY | Rheumatoid arthritis | 8 | 9.4 | 2.80E-06 | 12.3 | 5.40E-05 |
| KEGG_PATHWAY | Pertussis | 7 | 8.2 | 1.50E-05 | 12.6 | 2.40E-04 |
| KEGG_PATHWAY | Chemokine signaling pathway | 9 | 10.6 | 5.00E-05 | 6.5 | 7.10E-04 |
| KEGG_PATHWAY | Complement and coagulation cascades | 6 | 7.1 | 1.30E-04 | 11.7 | 1.70E-03 |

| | | | | | | |
|---|---|---|---|---|---|---|
| KEGG_PATHWAY | Amoebiasis | 6 | 7.1 | 9.70E-04 | 7.6 | 1.10E-02 |
| KEGG_PATHWAY | Cytosolic DNA-sensing pathway | 5 | 5.9 | 1.10E-03 | 10.5 | 1.20E-02 |
| KEGG_PATHWAY | Influenza A | 7 | 8.2 | 1.50E-03 | 5.4 | 1.40E-02 |
| KEGG_PATHWAY | Leishmaniasis | 5 | 5.9 | 1.70E-03 | 9.5 | 1.50E-02 |
| KEGG_PATHWAY | Epstein-Barr virus infection | 6 | 7.1 | 1.80E-03 | 6.6 | 1.50E-02 |
| KEGG_PATHWAY | Osteoclast differentiation | 6 | 7.1 | 2.50E-03 | 6.2 | 1.90E-02 |
| KEGG_PATHWAY | Salmonella infection | 5 | 5.9 | 3.00E-03 | 8.1 | 2.10E-02 |
| KEGG_PATHWAY | Staphylococcus aureus infection | 4 | 4.7 | 6.90E-03 | 10 | 4.50E-02 |
| KEGG_PATHWAY | Toll-like receptor signaling pathway | 5 | 5.9 | 7.10E-03 | 6.4 | 4.40E-02 |
| **Control-infected vs. uninfected** | | | | | | |
| ***GO Biological Process*** | | | | | | |
| GOTERM_BP_DIRECT | inflammatory response | 12 | 42.9 | 3.80E-13 | 23.1 | 1.00E-10 |
| GOTERM_BP_DIRECT | chemokine-mediated signaling pathway | 7 | 25 | 3.30E-10 | 72 | 4.30E-08 |
| GOTERM_BP_DIRECT | cell chemotaxis | 6 | 21.4 | 1.90E-08 | 67.4 | 1.60E-06 |
| GOTERM_BP_DIRECT | immune response | 9 | 32.1 | 3.40E-08 | 15.6 | 2.30E-06 |
| GOTERM_BP_DIRECT | cellular response to lipopolysaccharide | 6 | 21.4 | 3.00E-07 | 38.8 | 1.60E-05 |
| GOTERM_BP_DIRECT | chemotaxis | 6 | 21.4 | 4.40E-07 | 35.9 | 2.00E-05 |
| GOTERM_BP_DIRECT | positive regulation of neutrophil chemotaxis | 4 | 14.3 | 3.00E-06 | 132.7 | 1.10E-04 |
| GOTERM_BP_DIRECT | response to lipopolysaccharide | 5 | 17.9 | 5.60E-05 | 22.3 | 1.90E-03 |
| GOTERM_BP_DIRECT | response to molecule of bacterial origin | 3 | 10.7 | 5.90E-05 | 243.4 | 1.70E-03 |
| GOTERM_BP_DIRECT | neutrophil chemotaxis | 4 | 14.3 | 8.50E-05 | 44.2 | 2.20E-03 |
| GOTERM_BP_DIRECT | G-protein coupled receptor signaling pathway | 8 | 28.6 | 1.00E-04 | 6.5 | 2.50E-03 |
| GOTERM_BP_DIRECT | cellular response to interleukin-1 | 4 | 14.3 | 1.10E-04 | 41.1 | 2.30E-03 |
| GOTERM_BP_DIRECT | cellular response to tumor necrosis factor | 4 | 14.3 | 3.80E-04 | 26.5 | 7.80E-03 |
| GOTERM_BP_DIRECT | regulation of cell proliferation | 4 | 14.3 | 1.70E-03 | 15.8 | 3.20E-02 |
| ***KEGG Pathway*** | | | | | | |
| KEGG_PATHWAY | Chemokine signaling pathway | 7 | 25 | 5.30E-07 | 18.5 | 2.50E-05 |
| KEGG_PATHWAY | TNF signaling pathway | 6 | 21.4 | 9.70E-07 | 27.6 | 2.30E-05 |
| KEGG_PATHWAY | Legionellosis | 5 | 17.9 | 2.30E-06 | 45.5 | 3.70E-05 |
| KEGG_PATHWAY | Cytokine-cytokine receptor interaction | 7 | 25 | 2.50E-06 | 14.2 | 3.10E-05 |
| KEGG_PATHWAY | NOD-like receptor signaling pathway | 4 | 14.3 | 1.40E-04 | 35.1 | 1.30E-03 |
| KEGG_PATHWAY | Salmonella infection | 4 | 14.3 | 4.40E-04 | 23.7 | 3.50E-03 |
| **LSMMG-infected vs. control-infected** | | | | | | |
| ***GO Biological Process*** | | | | | | |
| GOTERM_BP_DIRECT | inflammatory response | 8 | 26.7 | 1.30E-06 | 13.1 | 3.60E-04 |
| GOTERM_BP_DIRECT | neutrophil chemotaxis | 4 | 13.3 | 1.40E-04 | 37.7 | 2.00E-02 |
| GOTERM_BP_DIRECT | chemokine-mediated signaling pathway | 4 | 13.3 | 1.80E-04 | 35 | 1.60E-02 |
| GOTERM_BP_DIRECT | wound healing | 4 | 13.3 | 2.50E-04 | 31.1 | 1.70E-02 |
| GOTERM_BP_DIRECT | immune response | 6 | 20 | 4.10E-04 | 8.9 | 2.30E-02 |
| GOTERM_BP_DIRECT | positive regulation of neutrophil chemotaxis | 3 | 10 | 5.20E-04 | 84.8 | 2.40E-02 |
| ***KEGG Pathway*** | | | | | | |
| KEGG_PATHWAY | Cytokine-cytokine receptor interaction | 7 | 23.3 | 3.40E-05 | 9.9 | 2.30E-03 |
| KEGG_PATHWAY | Amoebiasis | 5 | 16.7 | 1.70E-04 | 16.2 | 5.80E-03 |
| KEGG_PATHWAY | Legionellosis | 4 | 13.3 | 4.10E-04 | 25.5 | 9.00E-03 |
| KEGG_PATHWAY | Salmonella infection | 4 | 13.3 | 1.40E-03 | 16.6 | 2.40E-02 |
| KEGG_PATHWAY | Rheumatoid arthritis | 4 | 13.3 | 1.70E-03 | 15.6 | 2.20E-02 |
| KEGG_PATHWAY | TNF signaling pathway | 4 | 13.3 | 3.00E-03 | 12.9 | 3.30E-02 |

[*] GO Biological process and KEGG pathway enrichment analyses were performed using DAVID 6.8 using a threshold count of 2 and an EASE score of 0.05. Only terms and pathways with Benjamini-Hochberg values less than 0.05 are shown.
